# Deep-tissue transcriptomics and subcellular imaging at high spatial resolution

**DOI:** 10.1126/science.adq2084

**Published:** 2025-04-18

**Authors:** Valentina Gandin, Jun Kim, Liang-Zhong Yang, Yumin Lian, Takashi Kawase, Amy Hu, Konrad Rokicki, Greg Fleishman, Paul Tillberg, Alejandro Aguilera Castrejon, Carsen Stringer, Stephan Preibisch, Zhe J. Liu

**Affiliations:** 1 Janelia Research Campus, Howard Hughes Medical Institute, Ashburn, VA 20147, USA

## Abstract

Limited color channels in fluorescence microscopy have long constrained spatial analysis in biological specimens. Here, we introduce cycleHCR, a method that integrates multicycle DNA barcoding with Hybridization Chain Reaction (HCR) to overcome this limitation. cycleHCR enables highly multiplexed imaging of RNA and proteins using a unified barcode system. Whole-embryo transcriptomics imaging achieved precise three-dimensional gene expression and cell fate mapping across a specimen depth of ~310 μm. When combined with expansion microscopy, cycleHCR revealed an intricate network of 10 subcellular structures in mouse embryonic fibroblasts. In mouse hippocampal slices, multiplex RNA and protein imaging uncovered complex gene expression gradients and cell-type-specific nuclear structural variations. cycleHCR provides a quantitative framework for elucidating spatial regulation in deep tissue contexts for research and potentially diagnostic applications.

## Main text:

Understanding the spatial organization of molecular components within complex tissue samples is crucial for deciphering the biological regulations that underpin animal development and disease states. Marked advancements in microscopy, tissue clearing and expansion techniques over the past decades have enabled deeper imaging with enhanced clarity and resolution (1–3). However, a fundamental limitation of fluorescent imaging remains - it cannot simultaneously image multiple molecular components or color channels due to the restricted availability of labeling dyes and their spectrum overlaps.

Recent advances in high-throughput spatial omics, notably single-molecule in situ hybridization (ISH) techniques like MERFISH and seqFISH (4, 5), as well as in situ sequencing methods such as FISSEQ and STARmap (6, 7) have improved our capacity to spatially decode molecular identities, particularly for RNA species. These methods are further optimized for thick tissue applications ranging from 90 to 200 μm (8, 9). Despite notable improvements, several technical limitations remain. First, they rely on cross-round barcoding, which requires precise single-molecule localization of multiple RNA species in the same image, cross-round spot registration, and de-mixing of spots to assign them to specific genes. This approach is particularly sensitive to molecular crowding, where dense targets obscure the detection of others, limiting these techniques to sparse targets that can still be resolved when compressed in the same image (fig. S1). As a result, cross-round barcoding is not well-suited for multiplex imaging of high-abundance targets like proteins or densely packed RNA structures. Another limitation of these strategies is the lack of empirical ground-truth images for visualization and validation of each target, which is crucial for applications requiring direct image verification (10). Moreover, precise cross-round spot registration and de-mixing become increasingly challenging in thick tissue datasets especially when image tiling and stitching are required.

The development of split Hybridization Chain Reaction (HCR) techniques has begun to address several critical challenges in deep tissue imaging: First, the proximity binding requirement of split probes to initiate HCR allows for high specificity in the probe hybridization process. This feature enables single-shot imaging of RNA and protein molecules without the need for cross-round decoding and proofing (11–13). Second, HCR based signal amplification permits the use of objectives with low numerical apertures and long working distances, enabling fast and reliable deep-tissue detection, as recently demonstrated in whole-brain imaging (14). Third, the inherent single-shot nature of HCR imaging obviates the need for cross-round decoding, making this technique impervious to molecular crowding and suitable for imaging both sparse and dense targets. Achieving multi-round HCR imaging has been demonstrated through the time-consuming process of primary probe removal by DNase treatment and rehybridization (15). Currently, this method allows for only one round of imaging every 3 – 5 days, creating a bottleneck in the workflow and limiting the number of targets that can be effectively examined.

## cycleHCR: concept and implementation

**Fig. 1.**
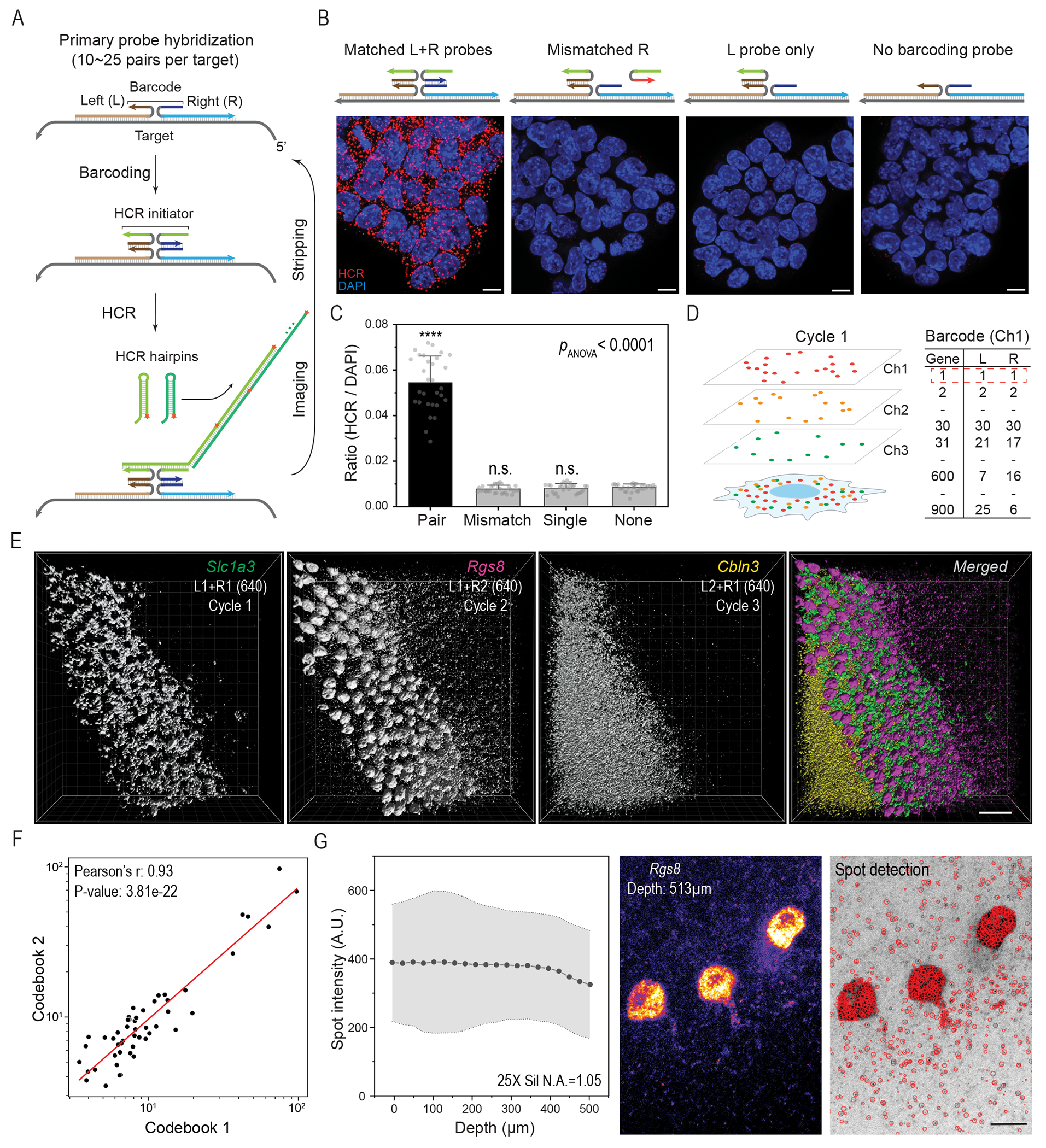
cycleHCR RNA imaging in thick tissues. **(A)** To target each RNA sequence with high specificity, we engineered DNA probe libraries with 10 to 25 pairs of 45-bp probes. Each pair consists of left and right probes with unique 14-bp barcode sequences for binding left (L) and right (R) readout probes with split HCR initiators, triggering the hybridization chain reaction (HCR). The arrowhead indicates the 3’ end of RNA or DNA. **(B)** The HCR amplification occurs only with perfectly matched L and R readout probe pairs. Mismatched probes, single probes, or absence of probes prevent HCR initiation, minimizing false positives. Target: *Trim6*; L2 + R2 (561). Scale bars: 10 μm. **(C)** Quantification of images shown in (B) is performed by comparing the HCR signal to the nuclear DAPI signal for normalizing HCR signals to the number of cells per region of interest (n = 30). The error bars represent standard deviations. A one-way ANOVA was conducted to assess significant differences across all groups, followed by pairwise *p*-values calculated using Bonferroni tests, with the no-barcode group serving as the reference. n.s., non-significant (*p* > 0.05); ****, *p* < 0.0001. **(D)** The mixing of 30 left and 30 right probes, each with unique divergent sequences, allows for the creation of up to 900 distinct barcodes for each fluorescence channel. Images are acquired using three separate fluorescence channels (488 nm, 561 nm, 640 nm), each channel harboring orthogonal B4, B2, and B3 HCR initiators. **(E)** This panel demonstrates cycleHCR labeling specificity in thick specimens for three distinct cell-type marker genes. Through sequential three-round cycleHCR imaging, we show specific labeling changes for *Slc1a3* (L1 + R1 at 640 nm, marking Bergmann glia cells), *Rgs8* (L1 + R2 at 640 nm, marking Purkinje cells), and *Cbln3* (L2 + R1 at 640 nm, marking the granule layer) in cerebellum specimens approximately 200 μm thick. 3D images were rendered using the normal shading mode in Imaris. Scale bar: 50 μm. **(F)** Two cycleHCR codebooks were used to measure the expression of the same set of 50 genes in mouse embryonic stem cells. Spots per cell (black dots) are plotted with linear regression in the red line. **(G)** Evaluation of spot detection over axial depth using a 25X silicone oil immersion objective. The left panel shows the relationship between spot intensity and depth, indicating relatively stable spot intensity up to 400 μm, with less than 10% reduction beyond 400 μm. The shaded region reflects standard deviation. The middle panel presents raw data acquired at a 513 μm depth in both sparsely and densely labeled regions, while the right panel displays spot detection marked with red circles overlaid on the raw image. Smaller red circles indicate the centers of these molecules are in other *z* slices. Scale bar: 20 μm

Here, we introduce cycleHCR technology that enables high-throughput, single-shot imaging of RNA and protein species within thick tissue specimens. At the core of cycleHCR RNA imaging lies the optimization and selection of 45-bp split primary probes (Fig. 1A and fig. S2). Distinguished by their high melting temperatures (> 90°C) for probe-RNA interactions, these probes ensure robust and stable binding under stringent conditions efficiently clearing away other components such as HCR chains and barcoding probes across multiple imaging cycles (fig. S3–S4). Another distinction of cycleHCR RNA imaging is the introduction of a barcoding phase, which uses 14-bp split Left (L) and Right (R) DNA barcodes (Fig. 1A). The specificity for each RNA species is determined by the split barcode sequence on the primary probe. The downstream HCR reaction is only triggered when both specific L and R readout probes with split initiators are present (Fig. 1B), achieving specific target recognition both in cultured cells and thick tissue specimens (Fig. 1B–E). Through the combination of 30 unique L and 30 unique R probes per color channel (Table S1), cycleHCR facilitates the generation of up to 900 distinct barcodes, thereby enabling the potential encoding of 2,700 targets across three channels (Fig. 1D).

The use of highly stable primary probes and precise barcoding negates the need for primary probe removal and rehybridization during multicycle HCR imaging. Through optimization, a consistent temperature of 32°C allows for signal removal in 20 minutes (fig. S4) and HCR amplification in 1.5 hours, achieving about half of the signal intensity of overnight amplification (fig. S5). Efficient barcoding is accomplished in under 30 minutes using high concentrations (150 ~ 200 nM) of L + R probes at this temperature. These optimizations collectively reduce the duration of one detection cycle to within 4 hours. Additionally, using an oxygen scavenger imaging buffer prevents photo-crosslinking during imaging (fig. S6), maintaining detection fidelity across multiple imaging cycles. Consistent with previous studies showing that longer probes with higher melting temperatures improve probe hybridization efficiency (16–18), we found that the 45-bp split probes produced substantially brighter signals than the traditional 25-bp split HCR probes under our stringent hybridization condition. Although adding the barcoding stage with L + R probes reduced fluorescent signals intensity by about 20%, the signals still exceeded the intensity observed with 25-bp probes (fig. S7). Systematic validation showed negligible cross-talks between L + R barcodes at 32°C (fig. S8 and S9A). Nuclear volumes remained consistent over 40 imaging cycles, indicating minimal sample deformation (fig. S9B). Further analysis revealed that HCR amplification at this temperature with 1.5 hours achieved 103.7-, 88.3-, and 121.3-fold amplification for the 488, 561, and 640 channels, respectively (fig. S10). Gene expression measurements using two distinct codebooks showed high correlation (*r* = 0.93) (Fig. 1F), validating the linearity and reliability of cycleHCR detection.

Transitioning towards full automation, our setup incorporates a programmable robotic arm for precise preparation of L + R readout mixes, and an automated imaging and fluidic system capable of executing cycleHCR protocols and image acquisition without the need of human supervision (fig. S3). This setup supports up to five imaging rounds analyzing 15 RNA species daily. Consistency and reproducibility in data analysis are facilitated by a scalable and portable Nextflow (19) image processing workflow that manages essential tasks such as image stitching, cross-cycle registration, spot detection, cell segmentation, and spot-to-cell assignment (fig. S11).

For high-resolution imaging of thick specimens, our setup utilizes spinning disk confocal microscopy equipped with silicone oil immersion objectives that offer long working distances up to 550 μm. A laser illumination uniformizer ensures homogeneous illumination (fig. S12), facilitating robust image stitching with BigStitcher (20) (fig. S13–S14). By integrating hydrogel-embedding and enzymatic digestion-based tissue clearing with cycleHCR, we achieved three-dimensional (3D) visualization of subcellular RNA patterns with reliable spot detection in thick tissues, spanning both sparsely and densely labeled regions over 500 μm, using RS-FISH (21) (Fig. 1G). For example, within a ~200 μm section of mouse cerebellum (fig. S14 and S15A; Movie 1), we observed dense *Slc1a3* mRNA clusters in Bergmann glia (fig. S15B), *Rgs8* transcriptional bursting sites in Purkinje nuclei (fig. S15C), and *Rgs8* transcripts along dendrites (fig. S15D). Approximately 50% of Purkinje cells displayed two *Rgs8* bursting sites, with no cell exceeding this number (fig. S15E), suggesting a diploid genome in Purkinje cells. RNA spot intensities remained consistent across the entire imaging depth (fig. S16).

## Whole-embryo transcriptomics imaging

**Fig. 2.**
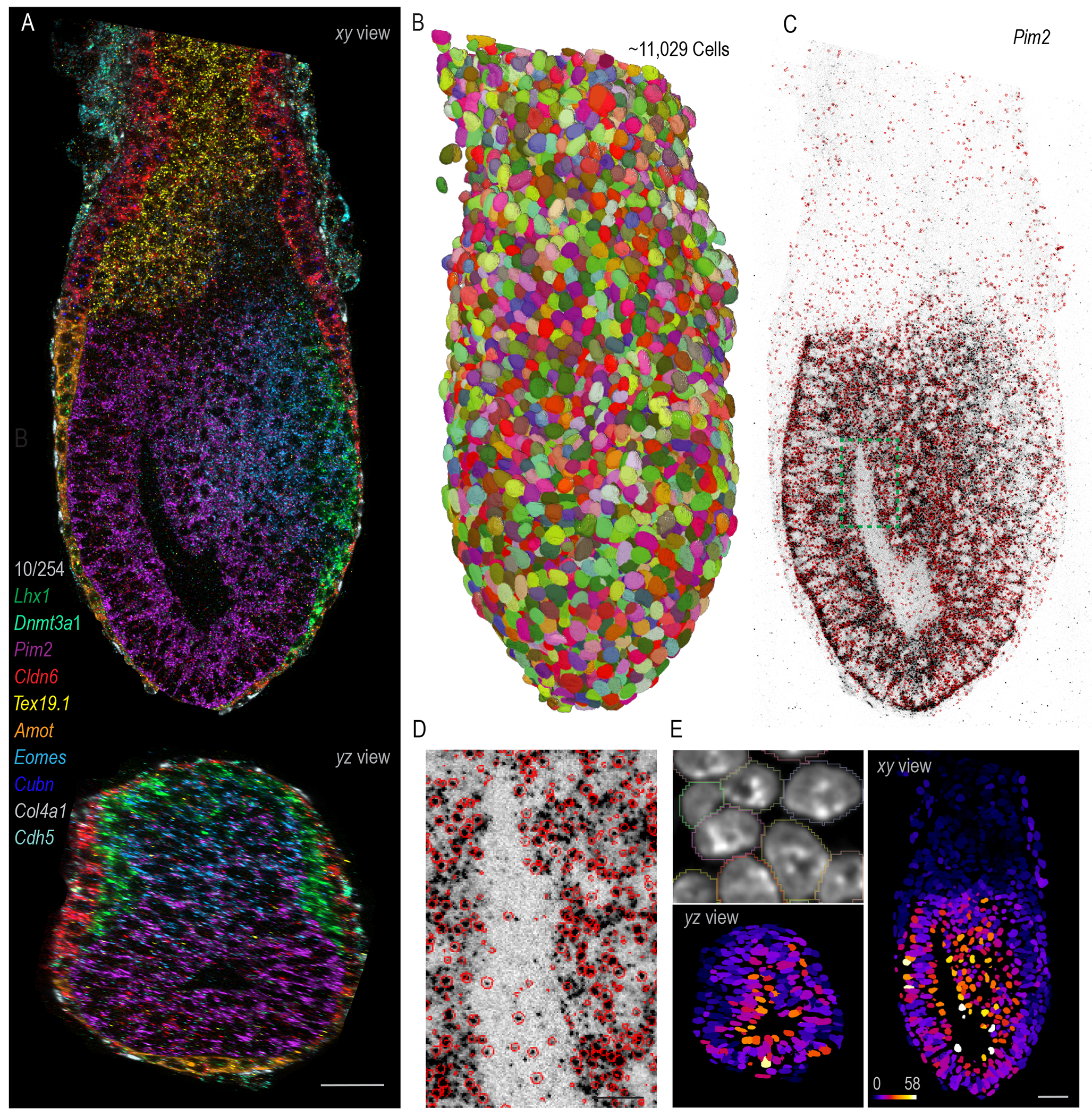
Whole-embryo transcriptomics imaging across a depth of ~310 μm. **(A)** Orthogonal *xy* and *yz* views of a 10-color composite image displaying the expression patterns of 10 genes (*Lhx1*, *Dhmt3a1*, *Pim2*, *Cldn6*, *Tex9.1*, *Amot*, *Eomes*, *Cubn*, *Col4a1*, and *Cdh5*) out of 254 genes imaged by cycleHCR in an E6.5-7.0 mouse embryo. The slice views were rendered by the maximal intensity projection (MIP) view using Imaris software. Scale bar: 50 μm. **(B)** Random colored masks for 11,029 cells segmented by Cellpose and rendered by ORS Dragonfly software. **(C)** An inverted raw image showing the detection of *Pim2* mRNA transcripts at the single-molecule level (black spots), with single-molecule localizations encircled in red. **(D)** A zoomed-in view of the region marked in (C), providing enhanced detail on the accuracy of single-molecule localization. Smaller red circles indicate the centers of these molecules are in other *z* slices. Scale bar: 10 μm. **(E)** 3D spatial gene expression maps for *Pim2* mRNA, with spots assigned to individual cells based on Cellpose masks that are slightly dilated compared to DAPI labeled nucleus. Cells in the resulting *xy* and *yz* views are color-coded based on transcript counts indicated by the provided color map. Scale bar: 50 μm.

To explore gene regulation and cell-fate determination in early development, we focused on an intact E6.5-7.0 mouse embryo, requiring ~310 μm axial coverage. Leveraging cycleHCR, we performed whole-mount transcriptomics imaging of 254 lineage-specific genes and 12 non-targeting barcodes through 89 imaging cycles over 20 days, achieving edge-to-edge imaging clarity and robust RNA spot detection (Fig. 2A–D and fig. S17; Movie 2; Table S2). High system stability and lack of substantial sample deformation enabled accurate cross-cycle image registration (fig. S18). The resulting single-shot images revealed distinct mRNA localization patterns (fig. S19), highlighting complex transcriptional programs during early embryogenesis.

For single-cell gene expression analysis, we utilized Cellpose (22) for 3D nucleus segmentation, applying a specialist model trained on both *xy* and *yz* planes and refined through size filtering, reaching ~87.6% initial accuracy. Manual curation further refined segmentation, identifying ~11,029 nuclei (fig. S20). The set of 254 lineage-specific genes was selected to cover diverse cell types from E5.5 to E12.5 during early development (23, 24). We applied stringent localization parameters, with expected silent genes at this developmental stage and non-targeting barcode combinations typically having fewer than 200 spot counts across the embryo (Table S3), indicating a conservative false positive detection rate of ~0.018 spots per cell. After evaluating various spot-to-cell assignment methods (fig. S21–S22), we adopted the most conservative approach, assigning spots based on Cellpose masks which are slightly dilated compared to nuclei (Fig. 2E). This strategy only captures spots in proximity to or within the nucleus and minimizes contamination for accurate 3D gene expression mapping at the single-cell level (Table S4). Furthermore, for densely expressed targets such as *Col4a1*, the spot counts per cell show a strong correlation with overall fluorescence intensity at the single-cell level (fig. S1), indicating reliable and unsaturated gene expression estimation for high-abundance transcripts. Our data showed significant correlation (P-value: 5.74e-13) and spatial alignment with previously published scRNA-seq, in situ hybridization (ISH) and immunohistochemistry (IHC) data (23, 25–36) (fig. S23–S24), validating the labeling specificity and detection sensitivity of cycleHCR.

## 3D cell-fate mapping

**Fig. 3.**
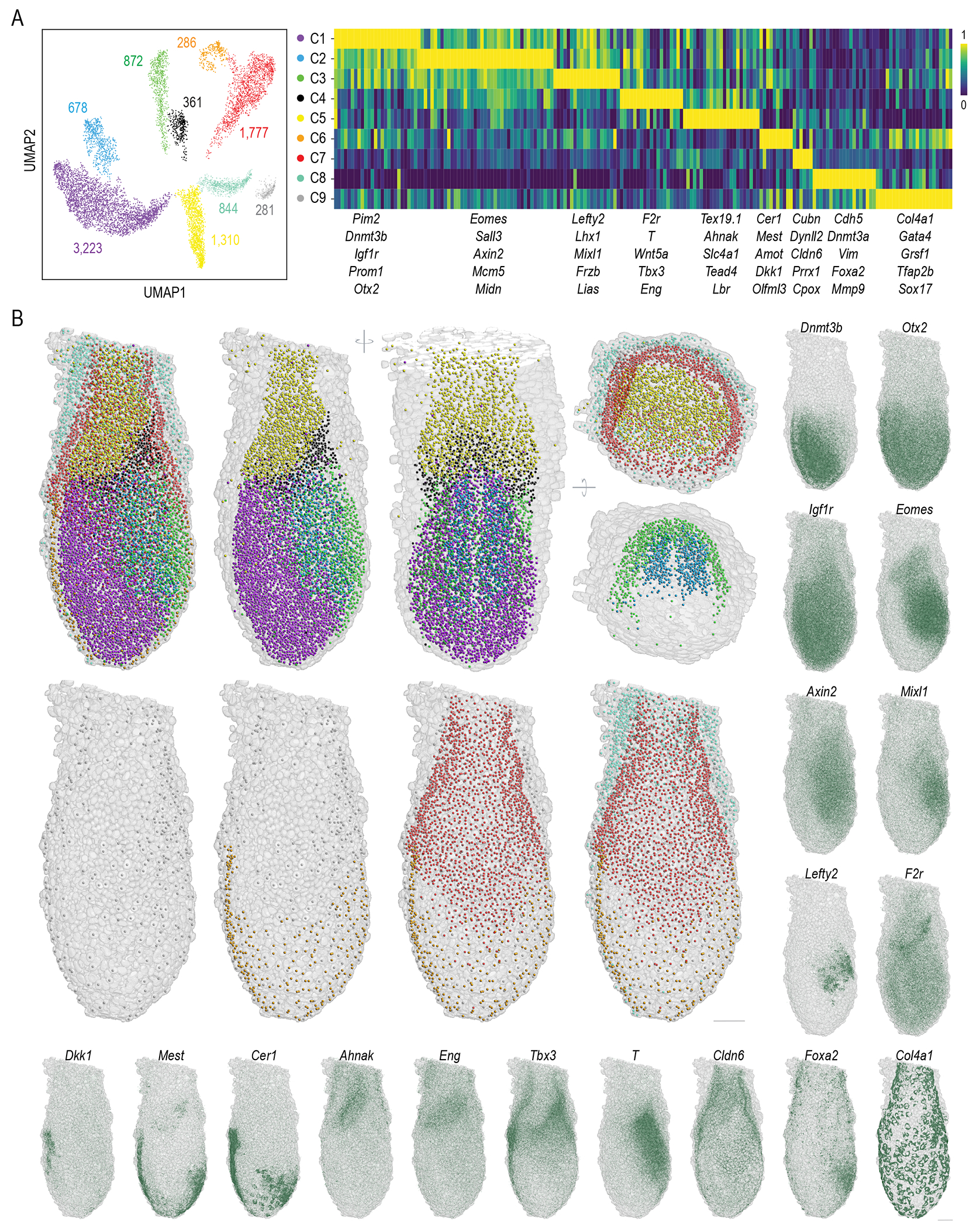
3D cell-fate map reconstruction. **(A)** UMAP analysis and gene clustering on single-cell transcript counts for 186 expressed genes detected by cycleHCR identify 9 distinct cell clusters. The number of cells within each cluster is indicated in the left panel. The right panel shows the clustering of the 186 genes, with 5 representative genes for individual clusters highlighted below. The color map represents the min-max scaled expression for each gene across clusters. **(B)** A 3D cell-map, color-coded per (A), displays the center of mass for each cell with a surrounding mesh for nuclear outlines. The left panel shows all 9 clusters. Upper middle panels provide views of clusters C1-C5 from the same and a ~90-degree rotated orientation, and the right panel displays the extra-embryonic layers (C5, C7, C8, and C9) alongside two closely situated intra-embryo clusters (C2 and C3). Lower panels detail the spatial distribution of outer layer clusters (C6-C9). Expression patterns for 18 selected genes are shown in the right and bottom zoom-out images by overlapping raw RNA localization patterns (dark green) onto the embryo mask. Images were rendered using the Napari Python package. Scale bars: 50 μm.

After initial filtering, we identified 186 actively transcribed genes and excluded 484 cells with low gene expression levels (fig. S25A–B). The detection rate of actively transcribed genes remained stable throughout imaging cycles, suggesting primary probes and RNA were well preserved through the imaging process (fig. S25C). Barcode swapping between active and silent genes revealed swapped expression patterns, confirming that low spot counts were attributed to low gene expression rather than barcode identity (fig. S26). Uniform Manifold Approximation and Projection (UMAP) (37) analysis on these genes revealed 9 distinct cell clusters (C1-C9) (Fig. 3A), expanding from 5 ~ 7 clusters previously recognized through single-cell sequencing at this developmental stage (23, 38, 39). cycleHCR identified clusters span a wide range of cell counts, from 286 in the smallest cluster (C6) to 3,223 in the largest (C1). Only 876 cells were left unallocated, as they lacked clear UMAP cluster association and were spatially scattered throughout the embryo without defined patterns (fig. S27). Gene clustering, UMAP imputation, and 3D spatial gene expression visualization showed extensive gene expression overlaps among clusters (Fig 3A; fig. S28), underscoring the intricate genetic interactions shaping early development.

Preliminary lineage assignment, based on marker gene analysis with comparison to previous studies (40, 41), suggests C1 as the epiblast (*Pim2*, *Dnmt3b* and *Prom1*), C2 as the primitive streak (*Eomes, Axin2 and Mcm5*), C3 as nascent mesoderm (*Lefty2*, *Mixl1* and *Frzb*), C4 as extra-embryonic mesoderm lineages (*T* (primitive streak and allantois progenitors (42)), *Tbx3* (allantois progenitors) and *F2r* (blood progenitors)), C5 as extraembryonic ectoderm (*Tex19.1, Ahnak and Tead4*), C6 as visceral endoderm (*Cer1* and *Dkk1*), C7, C9 as diverse endoderm lineages (*Col4a1*, *Sox17*, *Cubn*, *Gata4* and *Cldn6*) and C8 as putative endothelial cells (*Cdh5* and *Vim*).

To gain insights into the spatial organization and developmental context of these transcriptionally defined clusters, we proceeded to map them back to their 3D spatial locations within the embryo, revealing well-organized structures (Fig. 3B; Movie 3). Specifically, the 3D map shows the anatomical segregation of interior clusters - C1 (epiblast), C2 (primitive streak), C3 (nascent mesoderm), C4, and C5 (extraembryonic ectoderm) - into embryonic (C1, C2, C3) and extraembryonic (C5) domains, with C4 serving as a demarcation between the two domains. Within the embryo, the epiblast (C1), primitive streak (C2), and nascent mesoderm (C3), arrange into distinct, symmetrical layers across the midline. The endoderm clusters, C6, C7 and C9, also demonstrate spatial segregation, with C6 encasing the embryonic clusters (C1, C2, C3), C7 encircling the extraembryonic ectoderm (C5), and cells from C9 sparsely surrounding C6 and C7. Adding to this complex structure, the C8 covers endoderm layers, forming concentric rings.

The anatomical arrangement of identified clusters further refines the interpretation of potential cell fates. For example, the spatial positioning of C1 (Epiblast), C2 (Primitive Streak) and C3 (Nascent mesoderm) aligns with established developmental processes at this stage, including gastrulation and mesoderm migration (43, 44). Notably, C4, positioned between embryonic and extraembryonic domains, likely represents extra-embryonic mesoderm (allantois and blood progenitors (*F2r*, *Tbx3* and *Wnt5a*)) destined to form the umbilical cord connecting the fetus to the placenta (45, 46). The extraembryonic ectoderm (C5) is set to become the placental cone (47). Gene expression and spatial positioning identify C6 (*Cer1* and *Dkk1*) and C7 (*Cubn*) as visceral and extraembryonic endoderm cells directly interacting with the embryo (Fig. 3B) (48), with C6 more concentrated near the epiblast and C7 laterally positioned and enriched in the extraembryonic region. C9, lacking direct embryo contact and with highly specific expression of the gene encoding extracellular matrix protein - *Col4a1* (Fig. 3B), represents the parietal endoderm, eventually contributing to the parietal yolk sac (49). The putative endothelial cells (C8) mark emerging blood vasculature (50), crucial for supporting both embryonic and extraembryonic structures. Detailed cross-comparison with scRNA-seq data from the same developmental stage (23) reveals an under-representation of C3 (nascent mesoderm) and C9 (parietal endoderm), along with the absence of C4 (allantois and blood progenitors) and C8 (endothelial cells) from previous scRNA-seq UMAP clusters (fig. S24). These differences could be due to the low cell recovery rate (~39.5 cells per embryo) and potential selection biases during single-cell isolation (fig. S23). The precise enumeration and localization of minor or nascent cell groups, such as C4, C8, and C9, underscore the utility of cycleHCR for in situ spatial analysis of cell types in intact specimens.

## 3D spatial analysis of gene expression gradients and heterogeneity

**Fig. 4.**
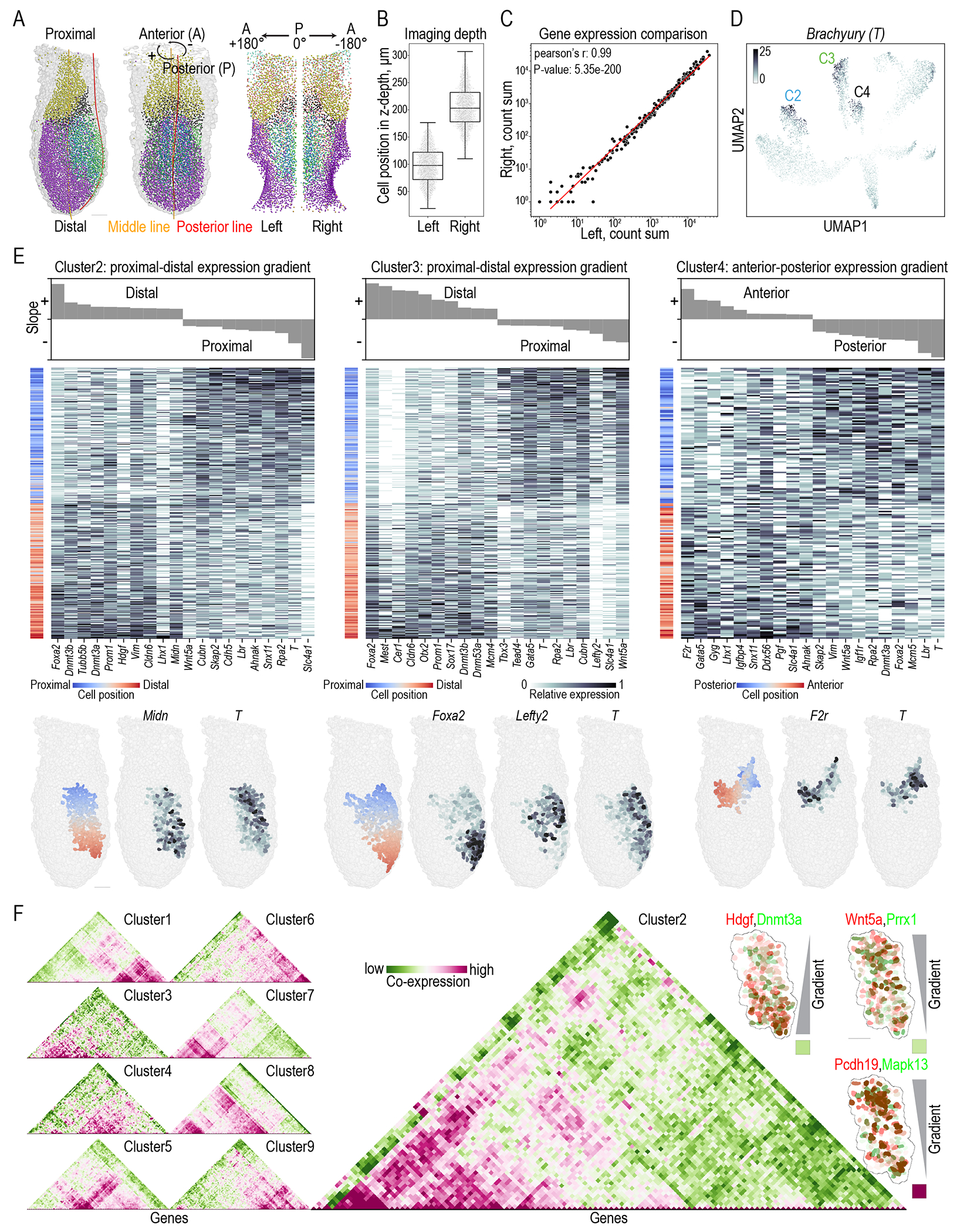
3D geometrical mapping of gradients and heterogeneity in gene expression. **(A)** Cell cartesian coordinates (*xyz*) were transformed along three biologically relevant axes in relation to the embryo structure: the proximal-distal axis (defined by distance along the midline), the anterior-posterior axis (defined by the angle between each cell, the midline, and the posterior line), and the radial axis (defined by the distance of each cell from the midline). Cells were divided into left and right sides based on the positive and negative values along the anterior-posterior axis. Cells are color-coded per Fig. 3A. **(B)** Cells on the left and right symmetric sides of the embryo occupy distinct *z*-positions during imaging. **(C)** Overall gene expression is highly correlated between the left and right sides of the embryo, suggesting that imaging depth does not substantially affect cycleHCR gene expression measurements. **(D)** UMAP imputation revealed that Brachyury (*T*) is over-represented in clusters C2, C3, and C4. **(E)** Top: Genes are ranked by their slopes across three independent biological axes for each cluster, from positive-ascending to negative-descending, with three selected panels displayed for clusters C2 (distal-proximal axis), C3 (distal-proximal axis), and C4 (anterior-posterior axis). Bottom: Color-coded cell positions and 3D gene expression maps for selected genes are displayed in the lower panel. Single-cell gene expression heatmaps for selected genes show gradients and substantial heterogeneity even among cells with similar gradient trends. The slope data for all 9 clusters along the 3 axes are in Table S5. **(F)** Gene co-expression analysis revealed distinct co-expression patterns for each cluster, with a zoomed-in view of the co-expression heatmap for cluster C2. This heatmap distinguishes gene pairs with the same expression gradient along the given axis but with varying correlations in single-cell expression, as illustrated by the three insets on the right with colored squares indicating co-expression levels. Gene co-expression matrices for all 9 clusters are in Table S6. Scale bars: 50 μm.

Extensive studies showed that cell fate and lineage commitment during early development begin with a heterogeneous “salt and pepper” pattern, which gradually forms specific expression gradients over time (51, 52). The 3D spatial information embedded within our cycleHCR data enables us to dissect these gradients and expression heterogeneity within each UMAP cluster. Establishing methods to analyze these patterns should advance our understanding of how new cell fates and structures emerge spatially. To analyze these patterns, we first transformed Cartesian *xyz* cell positions into three independent axes relevant to embryonic structure: distal-proximal, anterior-posterior, and radial (Fig. 4A). We found that gene expression on the left and right sides of the embryo is highly correlated, despite cells on each side being positioned at different axial locations during imaging (Fig. 4B–C). This further validates our ability to reliably detect gene expression across 3 color channels at different imaging depths.

The well-known Brachyury (*T*) was found to be over-represented in C2, C3, and C4 in the imputed UMAP map (Fig. 4D). We then analyzed gene expression gradients within these clusters using linear regression along the three defined axes, observing extensive gradients and heterogeneity in single-cell gene expression (Fig. 4E, Table S5). For example, in C2, *T* expression is higher in the proximal region, while *Midn* expression is higher in the distal region. In C3, *T*, the mesoderm marker *Lefty2*, and the definitive endoderm marker *Foxa2* occupy distinct spatial regions, with *Lefty2* and *Foxa2* exhibiting opposite gradients. In C4, *T* is concentrated in the posterior side, whereas the blood progenitor marker *F2r* is more highly expressed in the anterior side of the cluster. These findings illustrate how our dataset can help elucidate the emerging spatial expression patterns that underlie cell-fate determination during gastrulation.

Recent studies have demonstrated the necessity to investigate gene co-expression at the single cell level (53, 54). Next, we conducted a co-expression analysis revealing distinct cluster-specific gene co-expression programs (Fig. 4F; Table S6). By combining gradient and co-expression maps, we identified gene pairs that share similar gradients but exhibit varying co-expression levels within single cells, suggesting that genes with similar gradients do not necessarily mark the same cell population. Together, these resources and insights will aid developmental biologists in understanding complex genetic interplays during early development with high spatial precision.

## cycleHCR protein imaging

**Fig. 5.**
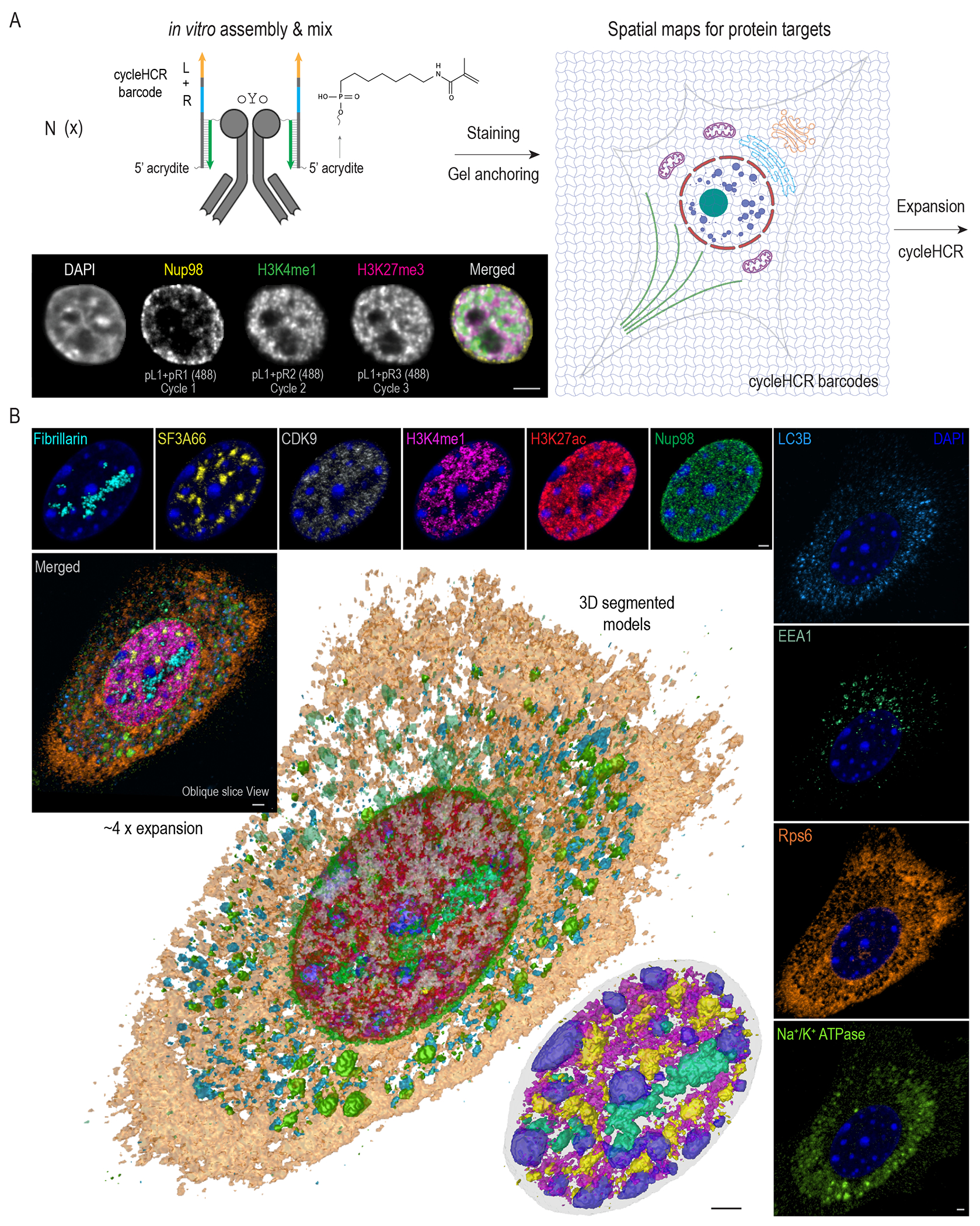
cycleHCR protein imaging with expansion microscopy. **(A**) cycleHCR protein labeling involves in vitro tri-functional antibody complex assembly. This method covalently attaches a docking sequence (green) to the antibody using the oYo linker. The sequence is then hybridized with a gel anchoring probe that contains a L + R cycleHCR barcode (light orange and blue) and a 5’ acrydite group for attachment to the polyacrylamide gel. Following the in vitro assembly, the sample is incubated with the antibody pool. After gel embedding, the cycleHCR barcodes are covalently incorporated into the gel matrix, allowing for subsequent tissue expansion and protein cycleHCR imaging. Arrowheads indicate the 3’ end of DNA. The specificity of this protein labeling strategy is demonstrated through sequential three-cycle imaging in unexpanded mouse embryonic stem cells by merely adjusting the right readout probes with the left readout probe fixed. The labeling shifts from the nuclear pore marker Nup98 (pL1 + pR1 at 488 nm) to the euchromatin marker H3K4me1 (pL1 + pR2 at 488 nm), and eventually to the heterochromatin marker H3K27me3 (pL1 + pR3 at 488 nm). **(B)** The integration of protein cycleHCR with expansion microscopy, illustrated by 3D surpass and oblique slice composite views (rendered by Imaris) of a primary mouse embryonic fibroblast after approximately 4X expansion. cycleHCR targeted 10 proteins, including Fibrillarin (nucleolar fibrillar regions), SF3A66 (nuclear speckles), CDK9 (RNA Polymerase II elongation), H3K27ac (active chromatin), H3K4me1 (enhancer regions), Nup98 (nuclear pores), LC3B (autophagosomes), EEA1 (early endosomes), Rps6 (ribosome), and Na^+^/K^+^ ATPase. Central to the panel are the 3D segmented models rendered by ORS Dragonfly software with a detailed zoom-in on the nucleus (bottom left) showing the spatial relationships among heterochromatin, Fibrillarin, H3K4me1, and SF3A66. Scale bars: 5 μm.

To harness the high specificity of cycleHCR for multiplex protein imaging, we next developed an antibody complex that simultaneously recognizes the target and anchors a cycleHCR barcode within the polyacrylamide gel matrix for imaging readout (Fig. 5A). The in vitro complex assembly begins by attaching a docking sequence to the antibody using a light-activated oYo linker (55), followed by annealing a gel anchoring probe that binds through DNA complementarity. This gel-anchoring probe carries a L + R barcode and a 5’ acrydite group for covalent gel incorporation. Once embedded, the barcode is permanently retained in the gel matrix, eliminating the need for the antibody to remain present and ensuring signal stability across imaging cycles. We have developed a distinct set of 25 Left (pL) and 25 (pR) Right protein barcodes, orthogonal to our RNA-focused set, enabling potential joint imaging of RNA and proteins. This method allowed precise, antibody-based protein detection over multiple cycleHCR rounds in cultured cells and mouse hippocampal slices (Fig. 5A; fig. S29–S30; Table S7).

Embedding barcoding oligos into the gel preserves spatial information during later processing steps, making cycleHCR ideal for coupling with tissue clearing and expansion microscopy. To integrate protein cycleHCR imaging with expansion microscopy (56), we stabilized the expansion gel with a second, non-expandable gel overlay. This ensured stable imaging and robust image alignment through multiple cycles (fig. S31; Table S7), allowing us to capture high-resolution images of 10 subcellular structures in primary mouse embryonic fibroblasts (Fig. 5B; Movie 4). 3D segmentation revealed the complex spatial organization of these structures (Fig. 5B; fig. S32; Movie 5), including distinct nuclear regions enriched for heterochromatin, nucleolar fibrillar centers, nuclear speckles, and active enhancers. These results highlight the potential utility of cycleHCR protein imaging in characterizing subcellular structures and their interrelations.

## Multiplex RNA and protein imaging via cycleHCR

**Fig. 6.**
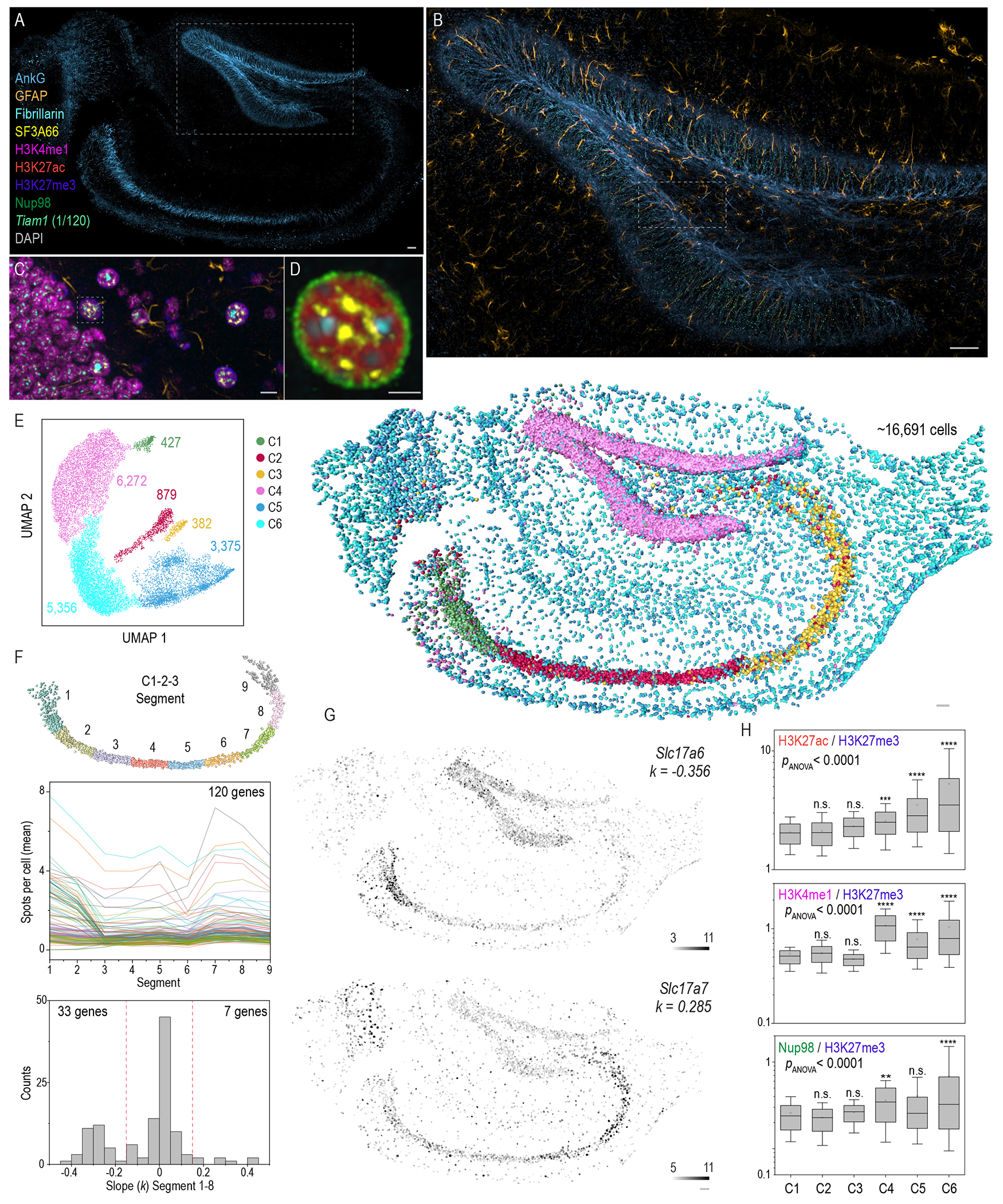
Multiplex protein and RNA imaging via cycleHCR in hippocampal slices. **(A-D)** Hierarchical images displaying structures at various length scales, with the axonal initial segment marker AnkG staining featured in (A). A closer examination of AnkG, astrocyte marker GFAP, and *Tiam1* cycleHCR RNA within the boxed region is shown in (B). Further zooming reveals Fibrillarin, SF3A66, H3K4me1, H3K27me3, and GFAP in the boxed region within (B), as depicted in (C). Zoomed-in views of Fibrillarin, SF3A66, H3K27ac, Nup98, and DAPI within the boxed region in (C) are presented in (D). Scale bars: (A-B) 50 μm; (C) 10 μm; (D) 5 μm. **(E)** UMAP analysis of single-cell transcript counts identifies 6 distinct cell clusters in the hippocampus. The number of cells within each cluster is indicated in the panel, with spatial distributions of approximately 16,691 assigned cells shown in the right panel, matching cluster colors in the left panel. **(F)** Top: cells in clusters 1-3 are divided into 9 distinct segments along their well layout middle line from left to right side. Middle: The average transcript counts per cell for 120 genes along Segments 1 to 9 are plotted using random colors, with multiple genes exhibiting peak gene expression at Segment 1, 7 or 8. Bottom: The distribution of linear slopes for all genes was calculated using the first 8 segments. 33 genes display decreased gene expression along segments with *k* <= −0.15, while 7 genes show increased gene expression along segments with *k* >= 0.15. **(G)** The solute carrier family protein *Slc17a6* showed descending gene expression levels along the C1-C3 axis, while the opposite is observed for *Slc17a7*. **(H)** Boxplots of nuclear antigen amounts (H3K27ac - active chromatin; H3K4me1 – active enhancers; nuclear pore complex subunit – Nup98) normalized to inactive chromatin (H3K27me3) intensity per nuclear mask to eliminate potential local staining imbalances. The lower and upper whiskers represent 10% and 90% values; the box represents the range from 25% to 75% percentile; the center line represents the median; the dotted line indicates the mean. The number of cells for each cluster is annotated in (E). A one-way ANOVA was conducted to assess significant differences across all groups, followed by pairwise *p*-values calculated using Bonferroni tests, using C1 as the reference. n.s., non-significant (*p* > 0.05); **, *p* < 0.01; ****, *p* < 0.0001. Scale bars (E-G): 50 μm.

Next, we investigated the potential of cycleHCR technology for unified multiplex RNA and protein imaging within hippocampal slices. We constructed a library targeting 120 genes that label diverse cell types in the hippocampus (Table S8). A 40 - 60 μm thick hippocampal slice was first stained with 8 cycleHCR barcoded antibodies (Table S7). After gel embedding, tissue clearing, and hybridization of the cycleHCR RNA library, 44 readout cycles were conducted over 11 days, with 40 cycles targeting RNA and 4 cycles targeting proteins. High system stability and precise cross-cycle image registration (fig. S33) ensured high-quality visualization of RNA and protein signals through cycleHCR rounds (Fig. 6A–D; fig. S34; Movie 6). Accurate 3D cell segmentation and spot identification facilitated single-cell gene expression analysis (fig. S35–S36; Table S9). Consistent with the mouse embryo data, gene expression maps generated by cycleHCR are well aligned with previously published ISH and RNA-seq data (57, 58) (fig. S37). Utilizing UMAP-based clustering, we uncovered 6 transcriptionally and spatially segregated clusters (denoted as C1-C6), corresponding to distinct neuronal and glial cell types (Fig. 6E; fig. S38–S39). We then repeated the experiment by imaging the transcriptome of the same 120 genes with 15 antibodies (fig. S40; Table S7; Movie 7), observing similar UMAP clustering (fig. S41). Further analysis revealed complex gene expression gradients along the middle line of C1-C2-C3, with 33 genes displaying descending expression and 7 genes showing ascending expression (Fig. 6F–G; fig. S42). The unified cycleHCR readout of RNA and protein compositions enabled cell-type characterization and sub-cellular structural investigation within the same specimen, unveiling cluster-specific nuclear structural variations (Fig. 6H and fig. S43).

## Discussion

Biological systems are complex networks of interconnected components, making it crucial to understand their interrelationships within intact tissue environments. Conventional fluorescence microscopy, constrained by fluorescent dye limitations, only allows for the observation of a limited number of components, limiting our comprehension of complex spatial regulations. Deep tissue imaging further faces challenges such as high autofluorescence and the necessity for objectives with low numerical apertures and long working distances, which are not well-suited for detecting dim single molecules. As a result, in situ hybridization based spatial omics imaging techniques are largely limited to thin tissue sections (4, 5, 10), leading to a loss of three-dimensional context. Recently, cross-round barcoding techniques have been extended to image thicker samples (90 – 200 μm) by deep-learning-based image processing (59), DNA branching amplification (8), rolling circle amplification (RCA) (9) and RCA combined with in *situ* hybridization (60).

The comparison of cycleHCR to these methods highlights both its advantages and its primary limitation which is lower throughput. This limitation stems from the Hybridization Chain Reaction (HCR) amplification process and its single-shot-per-target approach. However, cycleHCR effectively mitigates issues related to molecular crowding by not requiring spot-level registration and de-mixing across multiple rounds, enabling straightforward and robust spatial analysis of sparse and dense targets in deep tissues. The amplification process also enhances signal strength and contrast against the background, enabling imaging of intact tissues without the need for thin sectioning while providing empirical images for direct visualization and validation of each target.

Unlike other cross-round barcoding systems limited to RNA, cycleHCR offers the ability to image both RNA and protein using the same barcoding system. The integration of RNA and protein imaging bridges the gap between identifying cell types and understanding their subcellular protein arrangements, providing a comprehensive view of complex biological systems. Particularly, a promising direction is to use RNA information to create cell type labels, while applying artificial intelligence-based methods to automatically discover cell-type-specific protein structural features. 

Additionally, cycleHCR uses unlabeled barcoding probes along with only six common fluorescently labeled HCR hairpin probes for both RNA and protein imaging, greatly reducing probe synthesis costs compared to cross-round barcoding strategies as well as other amplification techniques like clamped or branched oligo structures (61, 62) and multiplex HCR (63), which require fluorescently labeled probes for individual barcodes or targets. Furthermore, cycleHCR is fully automated, requiring minimal human input, making it feasible for broad adoption. Coupling cycleHCR with advanced tissue-clearing techniques (14, 64) could improve reagent penetration and further enhance its deep-tissue performance. While we have shown that increasing the temperature to 32°C accelerates HCR signal amplification, the readout probe binding efficiency and required amplification levels may depend on factors such as gel composition, sample thickness, and target abundance, necessitating application-specific optimization. Further improvements in imaging depth and throughput are expected by using longer-working-distance water immersion objectives and combined imaging of multiple samples during each cycle. Additionally, light-sheet microscopy (65), known for its efficient illumination and reduced photobleaching, could complement cycleHCR, enabling deeper imaging while preserving high resolution, further enhancing its utility.

## Materials and methods summary:

### Cell culture and animal models

Mouse embryonic stem cells (ESCs) were cultured in Knockout DMEM supplemented with fetal bovine serum, LIF, and small-molecule inhibitors. Primary mouse embryonic fibroblasts and NIH3T3 cells were maintained in DMEM with 10% FBS. Cells were incubated at 37°C with 5% CO_2_. C57BL/6J mice were housed under standard conditions, and tissue collection followed Janelia Research Campus IACUC guidelines. Mouse brains were fixed in 4% PFA, cryosectioned, or flash-frozen for further analysis. Mouse embryos were fixed in 4% PFA and stored in 100% methanol at −20°C until further processing.

### Sample preparation

Coverslips were cleaned and chemically treated for optimal tissue adhesion. Tissue embedding in OCT and processing for sectioning followed standard histological methods. Cryosections and mouse embryos were mounted on silanized poly-D-lysine-coated coverslips.

### cycleHCR probe design and assembly

Primary probes for cycleHCR were designed using a systematic selection process to ensure specificity and stability. A split barcoding system was implemented for multiplexed detection, with HCR amplifiers assigned to distinct fluorescence channels.

### cycleHCR imaging workflow

Fixed tissues and cells were permeabilized, optionally stained with barcoded antibodies and embedded in hydrogels before hybridization with primary probes. A fluidic system automated L + R readout probe hybridization, HCR amplification, signal removal and imaging cycles. Imaging was conducted using a Nikon CSU-W1 spinning disk microscope with a laser uniformizer, a high-sensitivity camera, six laser lines (405 nm; 488 nm; 514 nm; 561 nm; 594 nm; 640 nm), and perfect focusing.

### Image processing and analysis

Large multi-tile images were stitched using BigStitcher (20) and aligned with bigstream-based image registration (15). Nucleus segmentation was performed with Cellpose 2 (22), and single-molecule spot detection used RS-FISH (21). Unsupervised UMAP clustering was applied for single-cell analysis, while spatial gradients and gene co-expression patterns were analyzed via linear regression and Spearman correlation.

### Visualization and data presentation

3D rendering and segmentation analysis were performed using Napari, Imaris, and ORS Dragonfly, selected based on visualization needs. Segmentation and spot detection accuracy were manually validated.

Full details on the materials and methods are provided in the supplemental materials.

## Supplementary Material

Supplementary Material

Table S3

Table S1

Table S2

Table S5

Table S6

Table S7

Table S8

Table S4

Table S9

## Figures and Tables

**Movie 1.** cycleHCR RNA imaging in a ~200 μm thick cerebellar brain slice. 3D representation of RNA transcripts from three genes imaged using a single-round, three-color cycleHCR method in a ~200 μm thick cerebellar brain slice. The genes visualized include *Slc1a3* (L5 + R5, 488 nm; green), marking Bergmann glia cells; *Stx1b* (L7 + R7, 561 nm; yellow), labeling the granule layer; and *Rgs8* (L6 + R6, 640 nm; magenta), identifying Purkinje cells. The movie was rendered using Imaris.

**Movie 2.** Whole-embryo transcriptomics imaging across ~310 μm depth. Slice-by-slice visualization of RNA transcripts from ten genes in an E6.5- E7.0 mouse embryo, captured using cycleHCR over an axial depth of ~310 μm. No depth-dependent intensity correction was applied. The movie was rendered using the ortho slice view in Imaris. Scale bar: 30 μm.

**Movie 3.** 3D cell-fate reconstruction. 3D reconstruction of nine UMAP clusters within a mouse embryo, with cells color-coded according to the palette used in Fig. 3A. The movie was rendered using Napari with the Napari-animation plugin.

**Movie 4.** 10-color cycleHCR protein imaging with expansion microscopy. cycleHCR imaging of 10 protein targets in an expanded mouse embryonic fibroblast. The movie was rendered using Imaris software.

**Movie 5.** 3D segmented models for 10 protein targets shown in Movie 4. Intensity-based segmentation and mesh rendering were performed using ORS Dragonfly software.

**Movie 6.** cycleHCR imaging of 8 protein targets in a hippocampal slice. cycleHCR images for 8 protein targets in a hippocampal slice, as part of the joint RNA and protein imaging shown in Fig. 6A–D. The movie was rendered using Imaris software.

**Movie 7.** cycleHCR imaging of 15 protein targets in a hippocampal slice. cycleHCR images for 15 protein targets in a hippocampal slice, as part of the joint RNA and protein imaging shown in fig. S40 and S41. The movie was rendered using Imaris software.

## Data Availability

All data are available in the manuscript or the supplementary materials. Analysis codes described in the Materials and Methods section have been published (66) and publicly available on GitHub (https://github.com/liulabspatial/cycleHCR, https://github.com/liulabspatial/CycleHCR-Pipeline).
