# Supplementary material for: Deep-tissue transcriptomics and subcellular imaging at high spatial resolution
Source: Science. Author manuscript; Available in PMC 2025 Apr 18. (PMC12005972; doi:10.1126/science.adq2084)
Supplement: Supplementary Material [file NIHMS2066229-supplement-Supplementary_Material.docx]

Supplementary Materials for

**Deep-tissue transcriptomics and subcellular imaging at high spatial resolution**

Valentina Gandin, Jun Kim, *et al.*

*Corresponding author: Zhe J. Liu, [liuz11@hhmi.org](mailto:liuz11@hhmi.org)

**The PDF file includes:**

Materials and Methods

Figs. S1 to S43

References (67-81)

**Other Supplementary Materials for this manuscript include the following:**

Tables S1 to S9

**Materials and Methods:**

**Cell lines**

mouse ESCs were maintained in Knockout DMEM (GIBCO 10829-018) supplemented with 10% fetal bovine serum ES cell qualified (ATCC® SCRR-30-2020™), 1X GlutaMAX™ Supplement 100X (GIBCO, 35050-061), 1X MEM non-essential amino acids solution, 100X (GIBCO, 11140050), 1X Antibiotic-Antimycotic (GIBCO, #15240062), 0.1 mM 2-mercaptoethanol (GIBCO, 21985-023), 1000 U/mL LIF (EMD Millipore, ESG1106), 1 μM PD03259010 (Millipore Sigma, PZ0162), 3 μM CHIR99021 (StemCell Technologies, 72052). Primary mouse embryonic fibroblasts (pMEFs) and NIH3T3 (ATCC, CRL-1658) were maintained in DMEM (GIBCO, 12320032) and DMEM (ATCC, 30-2002) respectively, supplemented with 10% fetal bovine serum, 1X GlutaMAX™ Supplement and 1X Antibiotic-Antimycotic. Cell lines were maintained at 5% CO_2_ at 37°C.

**Mice and tissue sections**

Male and female C57BL/6J mice (Jackson Laboratory, Strain #000664) (3 months old) were used in our study. Primary rodent work was performed in accordance with protocols approved by the Janelia Research Campus Institutional Animal Care and Use Committee (IACUC) guidelines. Mice were housed in a 12 h light/dark cycle. Animals were anesthetized with isoflurane and perfused with RNase-free PBS (15 mL) followed by 50 mL 4% paraformaldehyde (PFA) buffered to 0.1 M Phosphate Buffer (pH 7.4). After dissection, brains were fixed in 4% PFA overnight, rinsed into 1X PBS, immersed in 30% (w/v) RNase-free Sucrose (Sigma-Aldrich, S7903) in 1X PBS to shake overnight, until the brain sank. Cerebellum was dissected out and embedded in OCT (Fisher Scientific, 23-730-571). 200 μm cryosections of the cerebellum were cut around Bregma -5.80 mm. Cryosections were directly mounted onto PDL-coated silanized 40 mm coverslips.

For flash frozen tissue, animals were anesthetized with isoflurane, checked for lack of response after to toe pinch, and decapitated before the hippocampus was dissected out. The hippocampus was oriented in OCT within a Peel-A-Way™ embedding molds (Millipore Sigma, E6032), and snap frozen by placing the mold into a dry ice/ethanol mixture (an approximate ratio of 5:1) until the OCT had frozen solid (about 5 min). OCT blocks were acclimated to -14°C in Leica CM 3050S Cryostat for 1 hour before 40 μm cryosections were collected coronally around Bregma -1.46 mm. Cryosections were directly mounted onto PDL-coated silanized 40 mm coverslips.

Mouse embryos were obtained from non-hormone primed Hsd:ICR(CD-1) females (Envigo) mated in house with B6D2F1 (C57BL/6xDBA) males. Insemination was verified the next morning by the presence of a copulatory plug, and this day was defined as E0.5 days post fertilization (d.p.f). Timed pregnant females were euthanized by cervical dislocation, and the embryos were recovered in 4% PFA (Electron microscopy sciences) diluted in RNase-free PBS at 4°C. Embryos were removed from the uterus and dissected from the decidua and parietal yolk sac using fine forceps, leaving the ectoplacental cone attached to the egg cylinder, as described previously (*67*). Subsequently, the embryos were fixed in fresh 4% PFA (RNase-free) overnight at 4°C, washed twice for 5 minutes with RNase-free PBS-T (1% PBS; Tween) on ice, and dehydrated in a series of methanol/PBS-T dilutions (10 minutes each) on ice as follows: 1) 25% MeOH/75% PBS-T; 2) 50% MeOH/50% PBS-T; 3) 75% MeOH/25% PBS-T; 4) 100% MeOH. Lastly, embryos were incubated at -20°C overnight (>16 hours).

**Coverslip cleaning and coating**

40 mm coverslips (Bioptechs, 40-1313-0319) were cleaned as described previously (*68*) with the following modifications. For the silanized coating, coverslips were immersed in a solution of 0.2% Triethylamine (Sigma Aldrich, 471283) and 0.3% allyltrichlorosilane (Sigma Aldrich, 107778) in Chloroform for 30 minutes. For tissue adhesion, silanized coverslips were coated with 100 μg/ml poly-D-lysine (Sigma P7280) dissolved in 1X PBS overnight. For cell adhesion, silanized coverslips were coated with 1 μg/ml Laminin (Gibco, A29248) for 2 hours at 37°C.

**cycleHCR probe design and assembly**

*cycleHCR primary probe selection*

For precise single-shot RNA imaging using cycleHCR, a comprehensive multi-step probe selection process is applied to each target transcript, ensuring high specificity and robust signal amplification. This process encompasses: 1) A sliding window approach is used to identify an optimal 92-bp sequence within the target transcript for probe design. This sequence is divided into two segments of 45 bp each for the left and right probe pairs, with a 2-bp gap between them. The selection criteria for these probe halves include: a maximum secondary structure melting temperature (Tm) of less than 76°C; a maximum cross-hybridization Tm of less than 72°C; a GC content ranging from 30% to 90% and exclusion of sequences with six consecutive identical nucleotides (GGGGGG, CCCCCC, TTTTTT, AAAAAA). 2) The DNA-RNA probe binding Tm is calculated for each probe half, ensuring a Tm above 90°C. The Tm for DNA:RNA duplexes is typically 10-15°C higher than that for DNA:DNA interactions (*69*). Thus, Tm(probe:RNA) is conservatively estimated using the formula: Tm(probe:RNA) = Tm(probe:DNA) + 10°C, where Tm(probe:DNA) is calculated using the nearest-neighbor method (*70*) with a salt concentration of 1 Mol. Additionally, compared to DNA:DNA duplexes, RNA:DNA duplexes are less sensitive to formamide denaturation, particularly at high formamide concentrations (*71*). These selection criteria ensure probe-target interaction stability under stringent stripping conditions. 3) The final step in probe selection involves a thorough specificity check against the entire genome. This involves screening for 26-bp junction sequences, representing the unique overlapping region between left and right probe halves. Any probe pair matching more than once in the genome is discarded to eliminate the risk of non-specific targeting.

*L and R barcoding probe sequence selection*

For the selection and assembly of L + R barcoding probes, 14-bp RNA and protein left and right readout probes was initially generated by using the randseq() function in Matlab 2022. The probe sequences underwent rigorous screening to adhere to specific criteria, aiming to enhance their specificity and minimize non-specific interactions. These criteria included: 1) Exclusion of sequences containing runs of four or more identical nucleotides (GGGG, CCCC, TTTT, AAAA) to prevent formation of secondary structures that could impair hybridization efficiency. 2) Elimination of sequences with the potential to form dimers or hairpin structures, which could interfere with probe-target interactions. 3) Ensuring a GC content between 30% and 60%, balancing hybridization strength and specificity. 4) A melting temperature constraint of less than 45°C to facilitate selective binding under the assay conditions. 5) Screening each new sequence against all existing sequences and their complementary sequences in the library to ensure a maximum cross-hybridization melting temperature (Tm) lower than 32°C, thereby reducing the likelihood of non-specific cross reaction. Through this method, a total of 230 probes were successfully generated, with 180 designated for barcoding cycleHCR RNA targets and the remaining 50 for barcoding cycleHCR protein targets.

*L and R barcoding probe assembly*

The assembly of L and R barcoding probes for cycleHCR involves a division and pairing process to enable specific and efficient Hybridization Chain Reaction (HCR) for each color channel. The process is outlined as follows: 1) The 180 RNA barcoding probes are divided into two sets of 30 Left (L) and 30 Right (R) probes for each color channel. This division ensures a wide range of unique barcode combinations, facilitating the multiplexing capability of the cycleHCR system. 2) To prepare the L and R probes for HCR initiation, we link a specific 18-bp split HCR initiator sequence to the 5’ end of each left barcode, separated by a short spacer (AA). Similarly, the right barcode is linked to the 5’ end of the other 18-bp split HCR initiator separated by a short spacer (AA), specific to the same color channel. This initiator sequence was designed to trigger the HCR reaction in a color channel specific manner. We used B4, B1 and B2 split initiator and hairpin sequences for 488, 561 and 640 channels respectively and their sequences were disclosed in the previous report of the HCR v3.0 method (*12*). 2) Following the assembly, the L and R barcoding probes, now equipped with their respective HCR initiator sequences, were synthesized using Integrated DNA Technology.

*Primary probe library assembly*

For each RNA target, we chose between 10 to 25 probe pairs. The construction of these probe pairs involves the assembly of left and right primary probes as follows: 1) Left Primary Probe Assembly: A forward PCR sequence containing a T7 promoter (TAATACGACTCACTATAGCGTCATC) initiates the assembly. This is followed by the first 45-bp sequence of the selected 92-bp segment. A spacer sequence 'TT' is inserted next. The 14-bp left barcode sequence comes after the spacer. The assembly concludes with a reverse oligo sequence (CGACACCGAACGTGCGACAA). 2) Right Primary Probe Assembly: Similar to the left probe, it starts with the same forward PCR sequence with the T7 promoter. The 14-bp right barcode sequence is placed immediately after the promoter. A spacer 'TT' follows the barcode. The last 45 bp of the 92-bp primary probe sequence is then added. The assembly is completed with the same reverse oligo sequence (CGACACCGAACGTGCGACAA). Each assembled probe, whether left or right, ultimately spans a total length of 92 bp. The primary probe sequences used for the 254 gene and 120 gene libraries for the mouse embryo and hippocampal experiments are in Tables S2 and S8.

*Protein cycleHCR probe design and synthesis*

The 20-bp oYo docking sequences were generated using the randseq() function in Matlab 2022 and screened for cross-hybridization with a melting temperature (Tm) below 40°C. DNA sequences linked to oYo (5’ linked) were manufactured by AlphaThera, Inc. The gel anchoring sequence was constructed by concatenating the reverse complementary sequence of the oYo docking (18-20 bp), a 5-bp random sequence, the reverse complementary sequence of the right barcode, 2 bp of AA, and the reverse complementary sequence of the left barcode. The 5’ Acrydite modification (/5Acryd/) was added during oligo synthesis by Integrated DNA Technology (IDT). The sequences for oYo docking, gel anchoring, and L + R barcodes used in this study are listed in Table S7.

**DNA synthesis and purification**

ssDNA libraries were synthesized by Twist Bioscience. dsDNA was amplified using KAPA HiFi HotStart Polymerase (Roche, KK2502) using the following two primer sequences (T7 Forward: 5’-TAATACGACTCACTATAGCGTCATC-3’; Reverse: 5’-TTGTCGCACGTTCGGTGTCG-3’) and purified with DNA Clean and Concentrator-5 Kit (Zymo Research, 11-302). dsDNA was converted to RNA with HiScribe™ T7 High Yield RNA Synthesis Kit (NEB, E2040S) and purified using Monarch® RNA Cleanup Kit (NEB, T2040). An RNA/DNA mix was obtained by reverse-transcribing 200 pmol of RNA using 200 pmol of the Reserve primer (5’-TTGTCGCACGTTCGGTGTCG-3’) with Maxima H Minus Reverse Transcriptase (Thermo Scientific, EP0753) as per manufacturer instructions. The RNA was digested with Thermolabile USER (Uracil-Specific Excision Reagent) II (NEB, M5508) overnight before alkaline hydrolysis in 1 M NaOH at 65°C for 15 minutes. Hydrolysis was neutralized with 1 M acetic acid before purifying the libraries using ssDNA/RNA Clean & Concentrator (Zymo Research, 50-444-498).

**RNA cycleHCR in tissues and cells**

*Fixation and permeabilization*

Fixed tissues were washed 3 times in 1X PBS (for 5 minutes each, then incubated in ice-cold methanol (Fisher Chemical., A454-1), on ice, for 20 minutes, 5 minutes in ice-cold [50% methanol, 50% 2X SSC - 0.1% Tween] and 5 minutes in cold - [25% Methanol, 75% 2X SSC - 0.1% Tween]. 25% methanol was then removed by rinsing three times with 1X PBS and washing once in 1X PBS for 5 minutes (tissue ≤ 40 μm) or 10 minutes (tissue ≥ 100 μm). Tissue was permeabilized in [0.5% Triton, 1X PBS] for 30 minutes at room temperature, followed by 3 washes in 1X PBS for 5 minutes each.

Cells were fixed in 4% paraformaldehyde (PFA) in 1X PBS (Invitrogen, AM9625) for 10 minutes and permeabilized in 0.5% Triton for 15 minutes.

*Gel embedding and Proteinase K digestion*

After permeabilization, samples were incubated in 0.1 mg/ml Acryloyl-X SE (AcX) for 1 hour at room temperature. AcX (Invitrogen, A20770) was prepared as described (*72*). Samples were then washed twice in 1X PBS, 10 minutes each time. Before proceeding with the gelation step, samples were incubated for 5 minutes (tissue ≤ 40 μm) or 10 minutes (tissue ≥ 100 μm) in polyacrylamide (PA) solution [4% acrylamide/bis acrylamide (BioRad., 1610154), 60 mM Tris HCl pH 8 (Corning, 460131), 0.3 M NaCl (Corning, 46032]. For gelation, APS (Sigma, A3678) and TEMED (Sigma, T7024) were added to the PA solution with a final concentration of 0.03% and 0.15% respectively. Gelation chamber was assembled as described (*68*). Briefly, a pre-cleaned slide 75 x 50 mm, thickness 0.96 to 1.6 mm (Corning 2947-75X50) was coated with Gel Slick (Lonza, 182369) for 15 minutes and let air dry for at least 30 minutes. 100 μL of PA solution with APS and TEMED were added to the dry-glass plate and sample was slowly overlaid to avoid air bubbles. After 1.5 hour at room temperature, the gel fully polymerized and sample was gently detached from the glass plate. The silanized coating allows covalent binding of the hydrogel to the 40 mm coverslips, while the gel slick coating prevents the gel from sticking to the glass plate during polymerization. As a result, when the 40 mm coverslip is gently detached from the glass plate, the gel-embedded sample will remain stably bound to the coverslips during further processing.

Tissue samples were then digested with Proteinase K (NEB, P8107S) as described (*73*) with some modifications. Tissue was incubated in [2X SSC, 2% SDS, 0.5% Triton, 1:100 dilution of Proteinase K (800 units/ml; NEB P8107S)] for 16 hours in a humid chamber at 37°C.

Cells were digested as described (*68*).

*RNA quality control and hybridization with ssDNA libraries*

Steps that required small volumes (100 - 200 μL) were performed in custom-made hybridization chambers of the following dimensions: [0.12 mm chamber depth /20 mm diameter] for samples ≤ 40 µm; [0.5 mm chamber depth /19 mm diameter] for tissue 100 - 200 µm; [1 mm chamber depth/ 10 mm diameter] for embryos. To assemble the hybridization chambers, a 13-in-1 heavy duty hollow punch sets was used to cut the internal diameters indicated above from a round-silicon sheet (BIOPTECHS, 1907-1422-500 and 1907-1422-500). The prepared gasket was then securely affixed to a pre-cleaned slide 75 x 50 mm, thickness 0.96 to 1.6 mm (Corning 2947-75X50) using 100% silicone sealant (GORILLA). For the 0.12 mm x 20 mm chamber, an imaging spacer (Grace Bio-Labs Cat.# 654006) was attached to the pre-cleaned slide as described above.

Before incubating the sample with primary probes, RNA quality was assessed using our Alexa Fluor 488-conjugated-readout probe (RO22: 5’-GCCAAGATGGAGTTA-3’) targeting ribosomal RNAs. After proteinase K digestion, samples were washed twice in 5X SSC-T [5X SSC, 0.1% Tween] for 15 minutes. Ribosome probes were diluted in 10% EC Buffer [10% ethylene carbonate (Sigma, E26258), 10% dextran sulfate (Sigma, D4911), 2X SSC) at the following concentrations: 100 nM RO22 probes (samples ≤ 40 µm) or 200 nM (tissue ≥ 100 μm). Samples were incubated in the appropriate concentration of RO22 for 1 hour at room temperature and washed once (samples ≤ 40 µm) or twice (tissue ≥ 100 μm) in 10% formamide solution [10% deionized formamide (Ambion, AM9342), 0.1% triton, 2X SSC) for 10 minutes. Before imaging, samples were then washed in 5X SSC-T before staining nuclei with 5 μg/ml DAPI.

After assessing RNA quality, samples were incubated with primary probe libraries. Primary probes were added to a total volume of 100 µL (for 0.12 mm x 20 mm or 1 mm x 10 mm diameter chambers) or 250 µL (for 0.5 mm / 19 mm chamber) hybridization solution [50% deionized formamide, 10% Dextran Sulfate, 2X SCC] in the following amount: ~2 µg of ssDNA libraries targeting 10 RNA species or less, ~25 µg of ssDNA libraries targeting RNA transcripts from 120 genes, ~50 µg of ssDNA libraries targeting RNA transcripts from 279 genes. Samples were hybridized with primary probes at 37°C for about 20 hours in RapidFISH Slide Hybridizer Oven (Boekel Scientific, Cat. #13-245-230). High stringent wash was carried out in 80% formamide solution [80% formamide, 4X SSC, 0.1% Triton) at 32°C for 20 minutes before readout probe hybridization.

**Readout probe hybridization and hybridization chain reaction (HCR)**

Samples were incubated with 200 nM readout probes in 10% EC Buffer for an hour at room temperature, rinsed three times in 5X SSC-T and subsequently washed once (samples ≤ 40 µm) or twice (tissue ≥ 100 μm) in 10% formamide solution [10% deionized formamide, 0.1% triton, 2X SSC) for 10 minutes. Samples were then washed three times in 5X SSC-T before HCR. Expanded samples were incubated with 200 nM readout probes for 3 hours and washes performed as described above.

For HCR, H1 and H2 amplifiers (HCR^TM^ Amplifiers: B4 fluorophore 488, B3 fluorophore 647, B2 fluorophore 560, Molecular Instruments, Inc.) were activated according to the manufacturer's instructions. After activation, H1 and H2 were added to the Amplification buffer [10% dextran sulfate, 0.1% Tween, 5X SSC) in a 1:100 ratio. In non-expanded samples, HCR was carried out for 1.5 hour at 32°C and washed twice in 5X SSC-T for 10 minutes before imaging. In expanded samples, HCR was carried out for 3 hours at 32°C.

**Stripping and reprobing**

Readout probes and HCR chains were removed by incubating the sample in 80% formamide solution (80% deionized formamide, 0.1% triton, 4X SSC) for 20 minutes at 32°C. Samples were then rinsed three times in 5X SSC-T and washed once in 5X SSC-T for 10 minutes before reprobing with readout probes for the consecutive round.

**Sample preparation for cycleHCR protein imaging**

*Fixation and permeabilization*

40 μm cryosections were removed from -80°C and immediately fixed. Tissues and cells were both fixed in 4% PFA for 10 minutes and permeabilized in 0.5% Triton in 1X PBS for 15 minutes at room temperature. After permeabilization, samples were incubated for 1 hour in blocking solution [0.25% Triton, 0.5 mg/ml salmon sperm DNA (Fisher Scientific AM9680), 10 mg/ml nucleases- and proteinases-free BSA (Sigma, 126609), 1X PBS]. During blocking, the antibodies conjugated with a unique docking oligo were assembled with the acrydite^TM^ 5’-gel anchoring oligos (IDT).

*In vitro assembly of antibodies-oYo-oligos with acrydite^TM^ 5’-gel anchoring oligos*

oYo Link® conjugated with each unique docking oligos were purchased from AlphaThera and crosslinked to the antibodies of interest according to the manufacturer’s instructions without any modification. For in vitro assembly of gel anchoring complex, a mix of 1 μl oYo-crosslinked antibodies, 7 μM acrydite^TM^ 5’-gel anchoring oligos and 10% dextran sulfate in 1X PBS was incubated for 1 hour at room temperature on an orbital shaker at 100rpm.

*Antibody staining*

Pre-assembled gel anchoring complexes were combined in 100 μl blocking solution and incubated with the sample overnight in a humid chamber at 4°C. To preserve RNA during incubation with primary antibodies, 6 units of SUPERase•In™ RNase Inhibitor (Invitrogen, AM2694) were added to the antibody mix. The next day, samples were washed three times in 1X PBS, before post-fixation in 4% PFA for 10 minutes. Samples were washed in 1X PBS and then incubated with 0.1 mg/ml Acryloyl-X SE (AcX) for 1 hour at room temperature. After AcX modification, non-expanded samples were embedded in a thin-hydrogel layer, digested with proteinase K and incubate with readout probes as described in RNA cycleHCR section. For simultaneous detection of protein and RNA, samples were incubated with primary probes after proteinase K digestion as described in RNA cycleHCR section.

**Protein cycleHCR with expansion**

Protein cycleHCR was combined with expansion microscopy using a protocol modified from a previous report (*74*). Samples were prepared as described in Protein cycleHCR. After overnight incubation with the mixture containing the pre-assembled gel anchoring complexes, samples were treated with 200 μg/mL acryloyl-X SE in 1X PBS for 1 hour at room temperature and rinsed twice with 1X PBS for 15 minutes each. Samples were then embedded in TREx1000 gelation solution [TREx1000: 1 M sodium acrylate (Sigma, 408220), 14% acrylamide (Bio-Rad, 1610140), 1000 ppm N,N’-methylenebisacrylamide (bis, Sigma, M7279), 1X PBS, 2000 ppm APS (Sigma, A3678), 2000 ppm TEMED (Sigma, T7024), and 100 ppm 4-hydroxy TEMPO (4HT, Sigma, 176141)] on a chamber composed of a Gel Slick-treated glass slide and 1-layer Scotch Magic Tape spacers (~56 µm, 3 M, #810) (see details in (*74*)). Gel was polymerized for 3 hours at room temperature to prevent denaturing the gel anchoring strand from the antibody docking strand. The cell-embedding gel was then detached from the coverslip, cut into smaller pieces, and digested in proteinase K (NEB, P8107S) diluted 1:100 in proteinase K digestion buffer (50 mM Tris-HCl pH 8, 500 mM NaCl, 1 mM EDTA, 0.5% Triton X-100, and 1% SDS) overnight at 37°C. The gel was washed 3 times in 1X PBS and three times in nuclease-free water for 30 minutes each time to fully expand. The expanded gel was then incubated in re-embedding gelation solution for 30 minutes on ice. The re-embedding gelation solution contains 4% acrylamide, 0.1% N,N’-methylenebisacrylamide (bis), 0.2% TEMED, 0.01% 4HT, and 0.2% APS. The re-embedding gel was then allowed to polymerize on a cleaned, silanized, and poly-D-lysine coated 40 mm coverslip in a chamber with 4-layer-Scotch Magic Tape spacers for 1 hour at 37°C. After re-embedding, the samples were loaded on our automated microfluidics imaging system to acquire protein cycleHCR data.

**Antibodies**

The following rabbit antibodies were used at 1:100 dilution unless otherwise indicated. Anti-Fibrillarin (Abcam, ab5821), Anti-Histone H3 (mono methyl K4) (Abcam, ab8895), Anti-Histone H3 (acetyl K27) (Abcam, ab177178), anti-HP1 alpha (Abcam, ab109028), anti-Histone macro-H2A1 (Abcam, ab232602), anti-Tri-Methyl-Histone H3 (Lys27) (Cell Signaling, 9733), anti-Matrin 3 (Abcam, ab151714), anti-GFAP (Abcam, ab278054) used at 1:200, anti-Nup98 (Cell Signaling, 2598) used at 1:30 dilution, anti-RPS6 (Abcam, ab225676), anti-RAB7 (Abcam, ab126712), anti-EEA1 (Abcam, ab2900), anti-LC3B (Abcam, ab192890), anti-Na/K ATPase (Abcam, ab76020), anti-CDK9 (Abcam, ab239364), anti-Pericentrin (Abcam, ab4448), and anti-PMP70 (Invitrogen, PA1-650).

The following mouse antibodies were used at 1:100 dilution: anti-SF3a66 (Abcam, ab77800), and anti-Ankyrin-G (Antibodies Inc., 75-146).

Human IgA and IgM, rat IgG2a and chicken IgG were not used due to incompatibility with the oYo-Link technology.

**cycleHCR fluidics and imaging system**

The fluidics system is designed for precision in flow control and mixing, essential for cycleHCR imaging processes. It incorporates an OB1 flow controller for managing the flow rate through the system, two MUX distribution valves for directing flow to specific channels and a BFS Coriolis flow sensor to provide feedback for accurate flow control. This setup accommodates 10 probe tubes for L + R readout probes, 4 solution types including buffers and washing solutions and 2 hairpin mix tubes - One for H1 hairpin mix and another for H2 hairpin mix. The real-time mixing of H1 and H2 hairpin mixes is regulated by alternating the flow between them using a 2 to 1 MUX valve, eliminating potential background amplification. Flow Rate: Optimized at 150 µl/min to balance efficiency and sample integrity. After primary probe hybridization, samples on a 40 mm coverslip are placed in a closed-top FCS2 chamber for imaging. This chamber is then positioned on a customized stage adapter on the microscope. All buffers used in the following fluidics steps were filtered through 0.22 μm vacuum filter (Corning, 431098).

*Fluidics Steps:*

1. Stripping Buffer (80% formamide, 4X SSC, 0.1% Triton) Incubation (20 min): Removes bound probes and prepares the sample for the next cycle.
2. Washing (20 min): Uses 5X SSC and 0.1% Tween buffer with 5µg/ml DAPI to clean the sample.
3. Readout Probe Incubation (30 min): Applies mixed Left and Right probes for a new cycle of target visualization. 200 nM of each readout probes were added to 1.8 mL of hybridization solution (10% Ethylene Carbonate, 5X SSC, 10% Dextran Sulfate)
4. Washing (20 min): Another round with 5X SSC with DAPI.
5. Hairpin Mix Injection (5 min): Introduces H1 and H2 mixes for signal amplification. H1 and H2 (stocked at 3 µM; from Molecular Instruments) were added to the amplification buffer (5X SSC, 0.1% Tween, 10% Dextran Sulfate) at 1:50 ratio. When injection is triggered, H1/H2 are mixed at 1:1 ratio to a working concentration of 1:100 (30 nM).
6. HCR Amplification (90 min): Enables the fluorescent signal to build.
7. Washing (10 min) as in step 4: Cleans excess reagents post-amplification.
8. Imaging Buffer (50 mM Tris HCl pH8, 2 mM Trolox (Sigma, 238813), 1mg/ml Glucose Oxidase (Sigma, G2133), 1:100 Catalase (Sigma, C100), 0.8% D-Glucose, 5X SSC) Injection (20 min): Prepares the sample with an oxygen scavenger to prevent photo-crosslinking and enhance signal strength.
9. TTL Signal Activation: Triggers the microscope for imaging after a final 10-minute incubation.

*Microscopy:*

The Nikon CSU-W1 spinning disk microscope, equipped with advanced features, is utilized for high-resolution imaging in cycleHCR technology. This setup includes:

1. A 25X CFI PLAN Apochromat Lambda S silicone oil immersion objective with a numerical aperture (N.A.) of 1.05 and a working distance of 0.55 mm, ideal for imaging intact mouse embryos and protein cycleHCR imaging in expanded primary mouse fibroblasts.
2. A 40X CFI PLAN Apochromat Lambda S silicone oil immersion objective with an N.A. of 1.25 and a working distance of 0.3 mm, suited for imaging brain tissue slices.
3. An uniformizer: Ensures even illumination across the field of view.
4. 6 Laser Lines (405 nm, 514 nm, 561 nm, 594 nm, 640 nm): Provide a range of excitation wavelengths for versatile fluorophore excitation.
5. A Hamamatsu BT Fusion Camera: Captures high-quality images with efficient signal detection.

For the 25X objective, the microscope operated in ultraquiet mode with a fixed framerate of 5.1 Hz, optimizing conditions for sensitive samples like intact mouse embryos and expanded primary mouse fibroblasts. For the 40X objective, imaging of brain tissue slices was performed with a 100 ms exposure in the standard camera readout mode. The Nikon’s Perfect Focusing System (PFS) maintained the *Z* position of the objective between imaging rounds, critical for long-term imaging stability. TTL signals automated the transition between imaging rounds and fluidic cycles, streamlining the cycleHCR process. Temperature control during imaging was ensured by heating the objective with a Tokai Hit Lens Heater controlled by a TPi controller (TPiE-LH), while the imaging chamber temperature was regulated using a Bioptechs FCS controller.

**Image stitching**

Large images composed of multiple 3D tiles were aligned using BigStitcher (*20*) and the corresponding BigStitcher-Spark framework for distributed execution. After re-saving image data into the multi-resolution N5 format, alignment was performed independently for each imaging round, initialized using the 20% overlap (in *xy*) between neighboring tiles. The calculation of the final 3D affine transformation for each tile consists of several steps: pairwise shift calculation using phase correlation, pre-viewing and filtering pairwise shifts, global optimization of pairwise shifts, and affine refinement of the alignment using the Iterative Closest Point (ICP) algorithm (*75*). The pairwise shifts were calculated using 8x8x4 (*xyz*) downsampling and averaging intensity from all channels, ICP refinement was also computed using 8x8x4 downsampling. The tiles were fused either by weighted average fusion or the one-tile-wins strategy. The weighted average fusion strategy computes the values of pixels in the overlapping regions using a distance-weighted (from the tile boundaries) average, which was used for visualizing protein labeling. The one-tile-wins fusion strategy does not perform averaging in overlapping areas, but instead copies (and interpolates) pixel values from the input tile that was imaged first (out of all overlapping tiles at any given pixel). The one-tile-wins strategy was used for calling spots from RNA labeling. This strategy solves decreased spot-detection frequency in the overlapping regions due to averaging pixel values when the same single-molecule spot is slightly misaligned in the different overlapping tiles.

**Image registration**

To adjust for shifts in the sample or field of view during multiple cycles of imaging, the images of different rounds were registered to the image from a reference round using the Python package bigstream (version 1.2.9) (*15*). The reference round was chosen based on manual inspection, and all other rounds were transformed such that the DAPI channels across different rounds align in 3D space.

For single-tile images, the global affine transformation was sufficient. The affine transformation matrix was obtained using the alignment_pipeline function with a downsampling factor of two and a subsampling factor of two. The obtained transformation matrix was then used in the apply_transform function to perform uniform translation, scale, sheer, and rotation on all pixels.

For stitched multi-tile images, the global affine and local deform transformations were sequentially performed. The affine matrix was calculated as above, and then the deform matrix was calculated using the distributed_piecewise_alignment_pipeline function initialized with the affine matrix. The two matrices were provided as sequential steps, affine and then deform, in the distributed_apply_transform function to first perform the global affine transformation to the whole image, followed by local deformable transformation uniquely defined for each pixel.

**Cell segmentation**

For 3D nucleus segmentation of the mouse embryo, a unified custom model was trained using the human-in-the-loop feature in Cellpose 2 (*22*) on *xy* and *yz* slices in which nuclei were manually labeled. Subsequently, 3D segmentation function (*76*) was utilized to perform the segmentation across all dimensions. Masks less than 1000 voxels were filtered out.

In contrast, for 3D nucleus segmentation of the hippocampal slice, two distinct custom models were trained separately for the *xy* and *yz* orthogonal views, again utilizing the human-in-the-loop feature in Cellpose 2. A custom 3D segmentation procedure was then implemented by computing the *xy* flows using the *xy* model, and computing the *yz* and *xz* flows using the *yz* model. Next, the consensus cell flow was calculated by averaging across *xy*, *yz* and *xz* flows and then the dynamics were run on the flows to compute the masks. Masks less than 1000 voxels were filtered out.

The segmentation accuracy was estimated by human inspection of raw DAPI images and corresponding masks. Further manual curation and size filtering to eliminate potential oversized doublets were performed using ORS Dragonfly software.

**Spot detection and spot-to-cell assignment**

The spot detection process involved three-dimensional single molecule localization using RS-FISH (*21*). Specific parameters for each experiment were initially selected using the interactive feature provided by RS-FISH, as detailed in fig. S17 and fig. S36. These parameters were carefully chosen to minimize false positive detections. Importantly, the same parameters were consistently maintained across all images within each series.

Spot-to-cell assignment was achieved by rounding the *x*, *y*, *z* coordinates of each spot. Subsequently, cell assignment was determined by matching the rounded *x*, *y*, *z* voxel value of the labeled cell mask image.

**Mask-based image quantification**

We utilized the *regionprops3()* function in Matlab 2023b, to compute center-of-mass and intensity measurements per mask on labeled and grayscale image volumes.

**UMAP analysis**

The cell-by-gene matrix was filtered based on the total number of spots per gene and the total number of spots per cell. The total counts per gene were plotted as a histogram on a logarithmic scale. For the embryo data, Otsu’s method (*77*) of thresholding was used to remove genes with low total counts using the Python package scikit-image (version 0.23.1) (*78*). Cells with less than 40 total counts were removed, resulting in the removal of approximately 5% of cells. For hippocampus data, Li’s method (*79*) of threshold was used to remove genes with low total counts. Cells with less than 10 total counts across the retained genes were removed, resulting in the removal of approximately 7.5% of cells.

The filtered count matrix was used as input for generating UMAP using the Python package Uniform Manifold Approximation and Projection (UMAP) (version 0.5.5) (*37*). The cells were clustered in an unsupervised manner using Python package HDBSCAN (version 0.8.33) (*80*).

**Gene-to-cluster assignment**

We assigned each gene to one of the identified clusters based on average counts per cell in each cluster. The filtered count matrix was normalized across each cell by dividing each element of the gene vector by the total count per cell, thereby normalizing for the potential overrepresentation of certain cells in gene vectors. For each gene, average count per cell for each cluster was calculated. The gene was assigned to the cluster with the highest average count. The unnormalized raw counts per cell were plotted in both UMAP and anatomical space to validate gene-to-cluster assignment.

**Subcluster gene expression gradient and co-expression analysis**

Cells belonging to a cluster of interest were subset to obtain a cluster-specific counts matrix. Only the genes expressed in at least 25% of the cells and having at least one cell with a spot count greater than 5 within the cluster were retained. To study the spatial gradient within each cluster, the log-transformed gene expression along the biological axis (proximal-distal axis, anterior-posterior axis, or radial axis) was fitted to a straight line. The slope of the linear fit indicates the spatial gene expression gradient (Table S5). To study the co-expression patterns of pairs of genes, the log-transformed gene expression matrix was used to compute a Spearman correlation matrix (Table S6).

For visualization of multiple genes exhibiting spatial gradients, the top and bottom 10 genes were selected based on the computed slope, and an equal number of top and bottom cells were chosen based on their enrichment for the selected genes. The normalized expression was plotted as a heatmap. To visualize multiple genes exhibiting co-expression patterns, hierarchical clustering was performed on the Spearman correlation matrix using Python package SciPy (version 1.13.0) (*81*).

**Image rendering**

To visualize and analyze 3D imaging data, we used three software platforms: Napari, Imaris, and ORS Dragonfly.

**Napari** (Python-based, open-source) was utilized for the generation of 3D renderings and movie creation. This software was specifically chosen due to its flexibility and tunability, allowing for high-quality visualizations of point clouds, masks, and single-channel raw images. We used Napari for handling and overlaying point clouds with masks and raw images, leveraging its command-line support for extensive customization.

**Imaris** (Bitplane, licensed software) was used for the 3D rendering and video creation of multichannel raw images. This software excels in rendering high-quality images using shading, maximum intensity projection, blending and oblique slicing techniques. We used Imaris for creating detailed 3D visualizations.

**ORS Dragonfly** (Free for academic use) was utilized for segmentation, multi-ROI manipulation, and 3D visualization. This platform was particularly effective for displaying segmented masks of mouse embryos, hippocampus regions, and subcellular structures. Additionally, we used ORS Dragonfly to generate movies of segmented 3D structures to further analyze spatial relationships.

The selection and rending of software selection for each panel is noted in the figure legends.

**figs. S1 to S43**


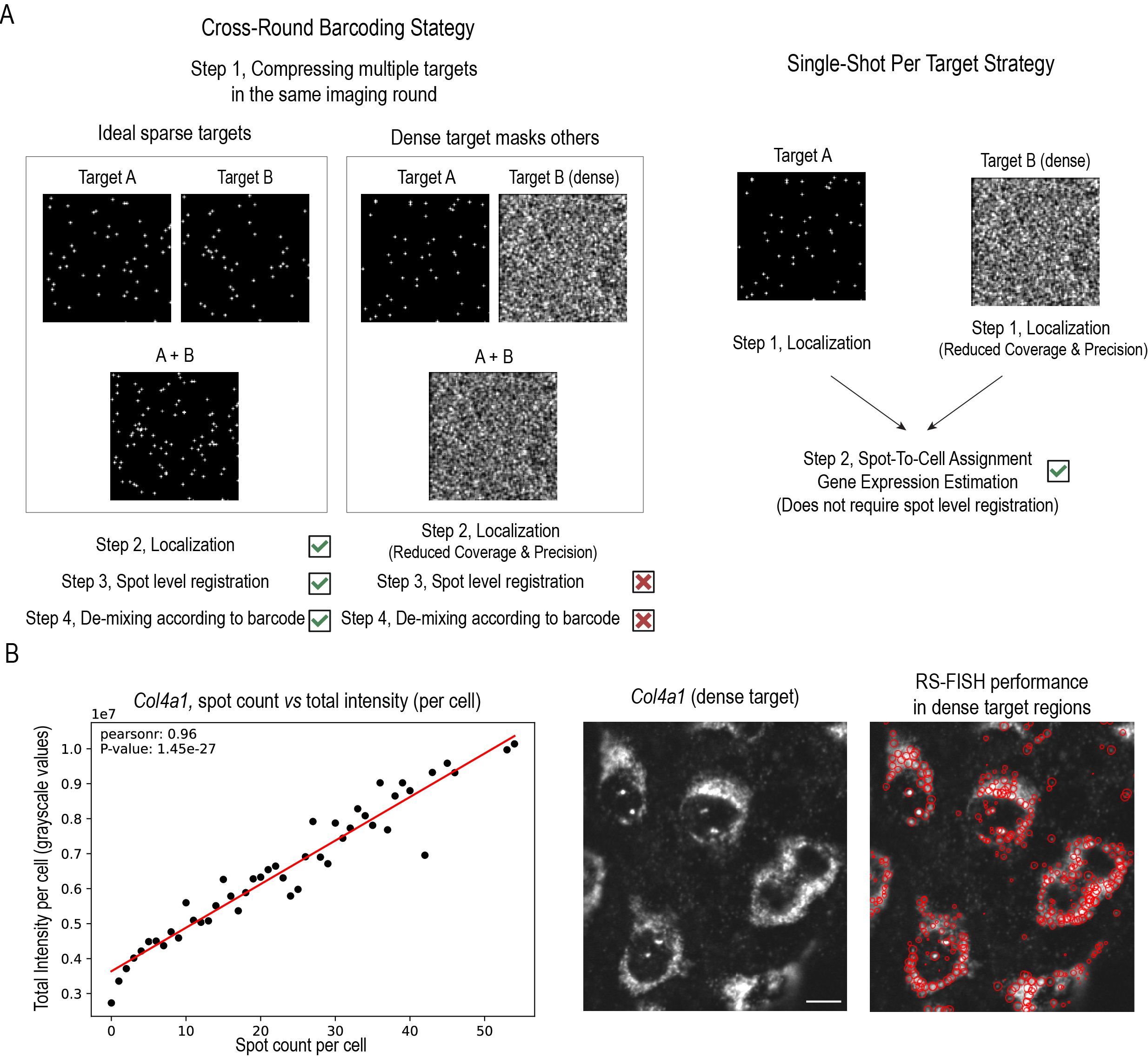


**fig. S1. Effect of molecular density on different barcoding systems.**

**(A)** Left: In the cross-round barcoding system, single-molecule signals from multiple genes are compressed into the same image, relying on precision spot-level localization and registration to de-mix spots across rounds and assign them to different genes. Dense targets compromise both precision and coverage in the localization step, affecting later de-mixing steps. Right: In the single-shot per-target strategy, determining molecule identity is unnecessary, and spot-to-cell assignment requires less precise localization. This strategy allows gene expression estimation even for dense targets, mitigating molecular crowding.

**(B)** RS-FISH localization for dense *Col4a1* targets in the mouse embryo. **Lef**t: Plot showing that localization counts are highly correlated with *Col4a1* RNA cycleHCR fluorescence total intensity per cell. Right: Images demonstrating RS-FISH performance in *Col4a1* dense regions, where single molecules are not clearly visible. Smaller red circles indicate the centers of these molecules are in other *z* slices. Scale bar: 10 µm.


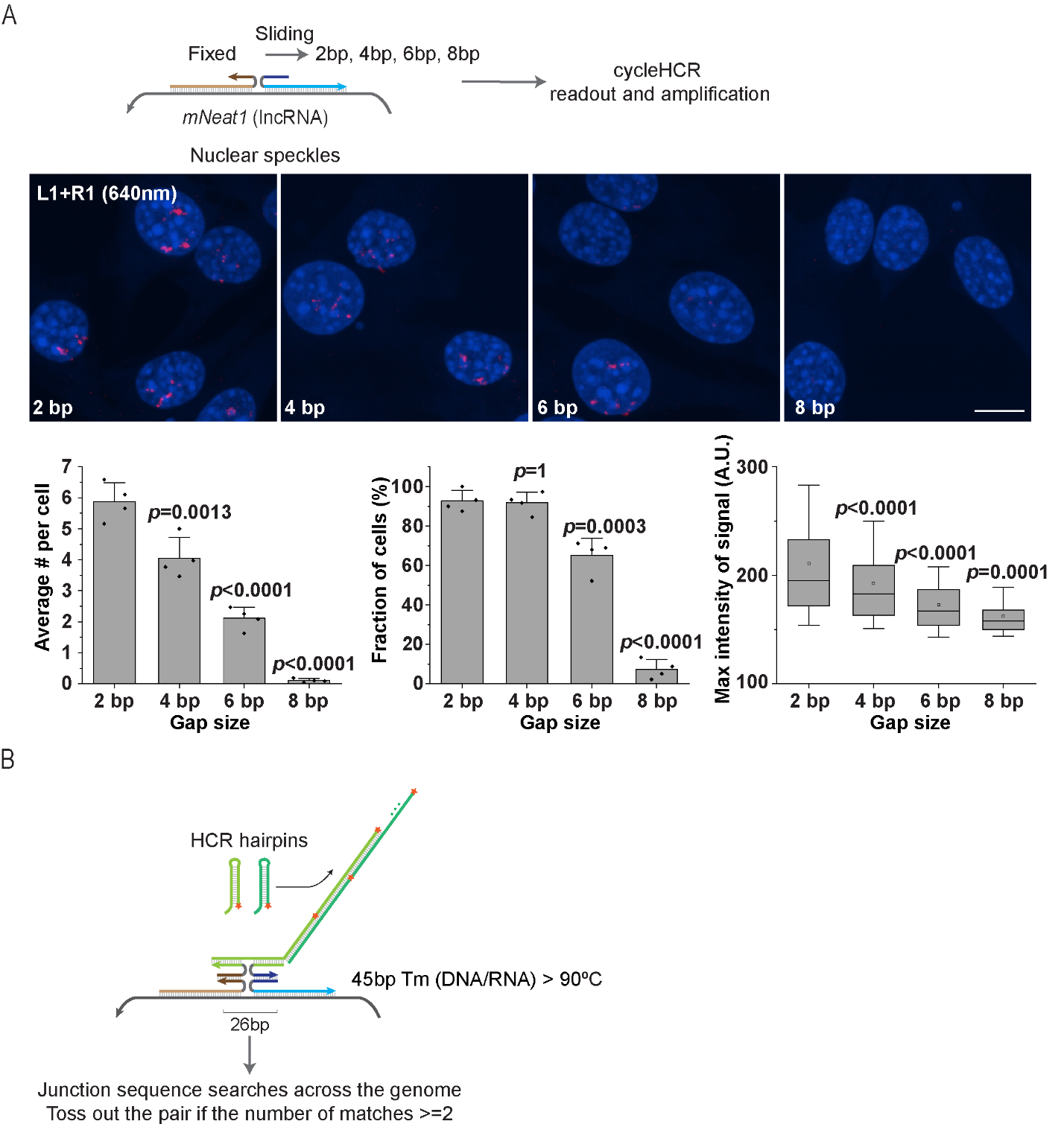


**fig. S2. Primary probe selection for cycleHCR RNA imaging.**

**(A)** Evaluation of gap sizes on cycleHCR detection and amplification efficiency. A single pair of a fixed left probe and a sliding right probe targets *mNeat1*, a long non-coding RNA localized in nuclear speckles. The average number of puncta per cell, fraction of cells containing puncta, and maximum intensity of puncta were quantified for gap sizes of 2 bp, 4 bp, 6 bp, and 8 bp. Data were collected from four fields of view for each group, with total cell counts of 122, 176, 213, and 169 cells for each condition, respectively. Error bars in bar plots represent standard deviations. The lower and upper whiskers represent 10% and 90% values; the box represents the range from 25% to 75% percentile; the center line represents the median; the dotted line indicates the mean. *p*-values were calculated with ANOVA Bonferroni tests by using the 2-bp group as the reference. Scale bar: 10 µm.

**(B)** cycleHCR uses split probe pairs to increase labeling specificity. Each probe pair consists of 45-bp left and 45-bp right probes separated by a 2-bp gap. The melting temperature (Tm) is calculated to ensure that each probe half binds to RNA with a Tm above 90°C. High Tm values contribute to the stability of probe-target interactions even under stringent stripping conditions. A final specificity check involves screening for 26-bp junction sequences across the genome sequence. Any probe pair with more than one genomic match is excluded to avoid non-specific targeting.

The arrowhead indicates the 3’ end of RNA or DNA.


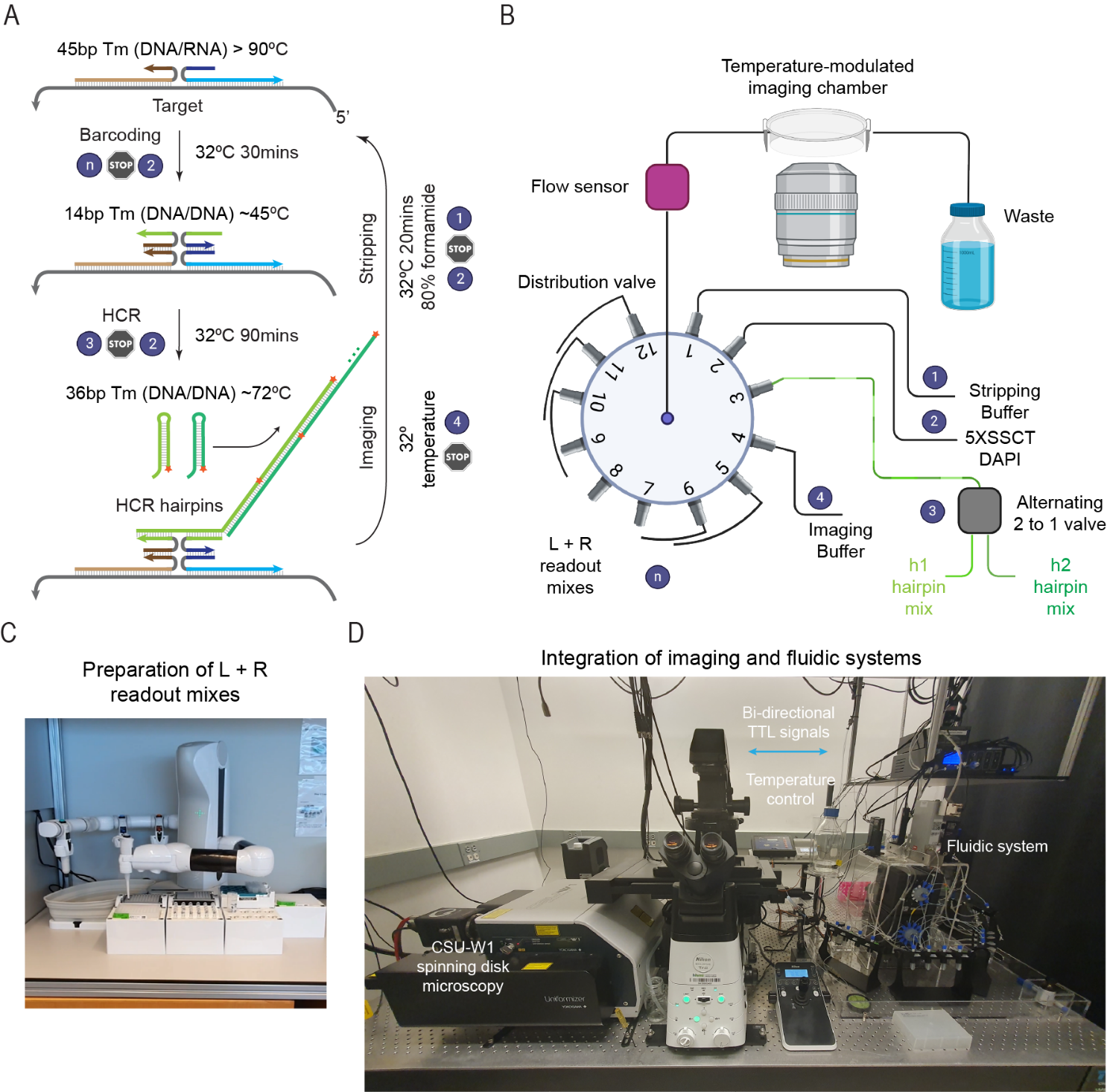


**fig. S3. Automation of cycleHCR procedures.**

**(A)** The cycleHCR protocol uses a consistent incubation temperature of 32°C across all steps after primary probe hybridization. Left and right barcoding probes, each carrying split HCR initiators, are hybridized to the primary probe for 30 minutes. Following a washing step, the HCR amplification process is initiated with h1 and h2 hairpins for 1 hour and 30 minutes. Post-amplification, a final washing step precedes imaging. A tailored stripping step then selectively removes HCR hairpins and left-right barcodes without disrupting the robust primary probe:RNA interactions, due to their high melting temperatures (>90°C). The arrowhead indicates the 3’ end of RNA or DNA.

**(B)** The fluidic system integrates an air pressure modulator and a flow sensor with Proportional-Integral-Derivative (PID) feedback to ensure precise control over the flow rate and volume of probes, washes, imaging, and h1/h2 solutions through a distribution valve. A 2-to-1 valve allows for real-time mixing of h1 and h2 solutions. These solutions then pass through a temperature-controlled imaging chamber before disposal into a waste collection bottle.

**(C)** The preparation process of Left and Right readout mixes utilizes a programmable pipetting robotic arm.

**(D)** The cycleHCR imaging setup includes a CSU-W1 spinning disk microscope, equipped with a temperature control system for both the chamber and objective, integrated with the fluidic system. Communication between the microscope and the fluidic system is facilitated via Transistor-to-Transistor Logic (TTL) communication protocol, ensuring synchronized operation between imaging and fluidic operations.


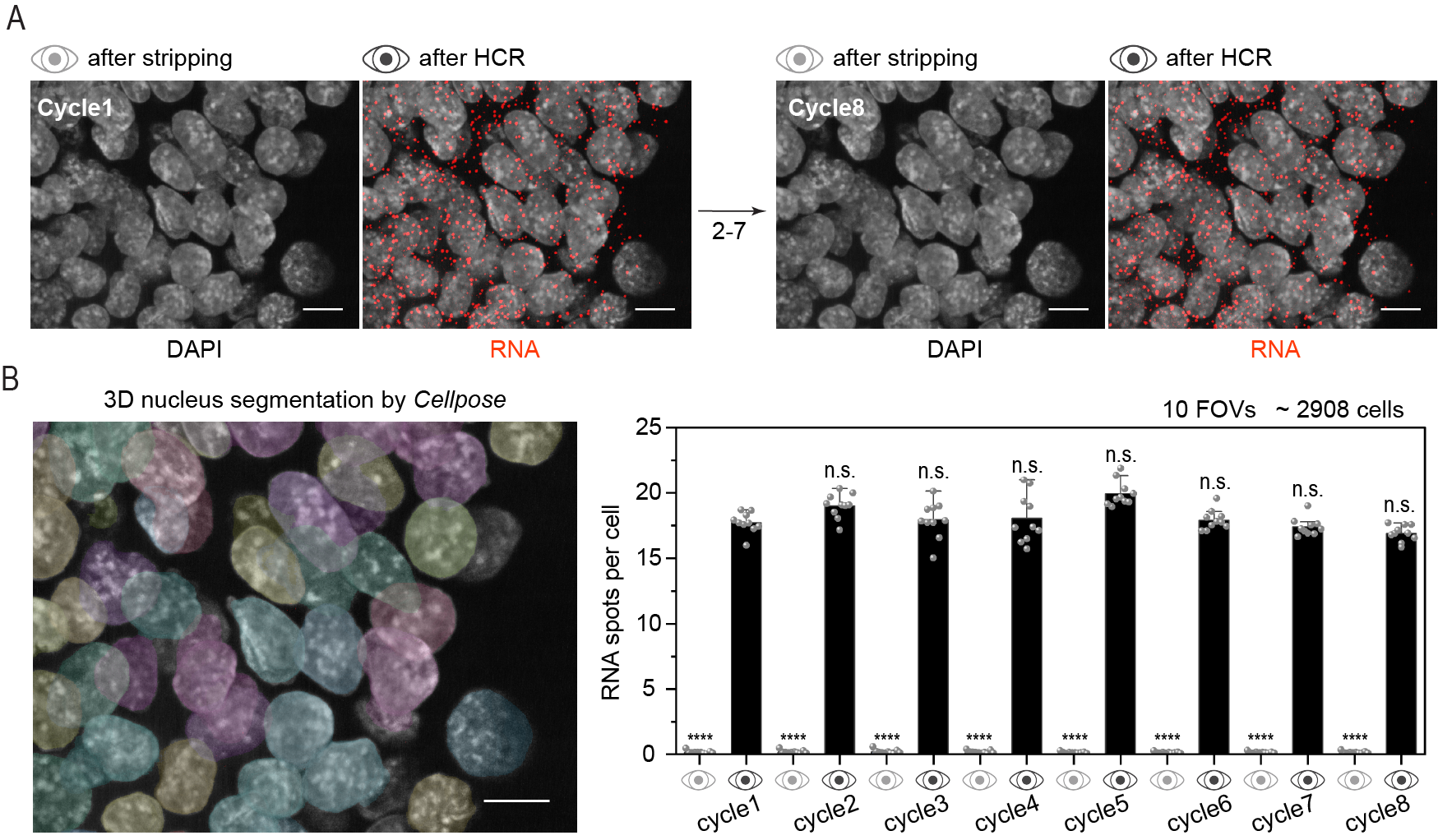


**fig. S4. Evaluation of stripping efficiency and stability of primary probes on RNA targets.**

**(A)** The **multicycle stripping and reprobing efficiency** of cycleHCR is evaluated by repetitively stripping and reprobing the same RNA target (*Trim6*; L2+R2 at 561 nm) across eight cycles. For each cycle, imaging data were captured post-stripping and post-HCR to assess the efficiency of probe removal and the consistency of signal amplification. Pairwise *p*-values were calculated with ANOVA Bonferroni tests by using the cycle1 after HCR group as the reference. n.s., non-significant (*p* > 0.05); ****, *p* < 0.0001.

**(B)** The **stability of primary probe-RNA interactions** was quantitatively evaluated using nuclear segmentation with Cellpose (left panel), enhanced by a custom-trained model, alongside 3D single molecule localization with RS-FISH. RNA spot counts were determined for each cell within 10 fields of view, totaling approximately 2,908 cells. The analysis revealed no significant decrease in signal intensity across the eight cycles (right panel).

Scale bars: 10 µm.


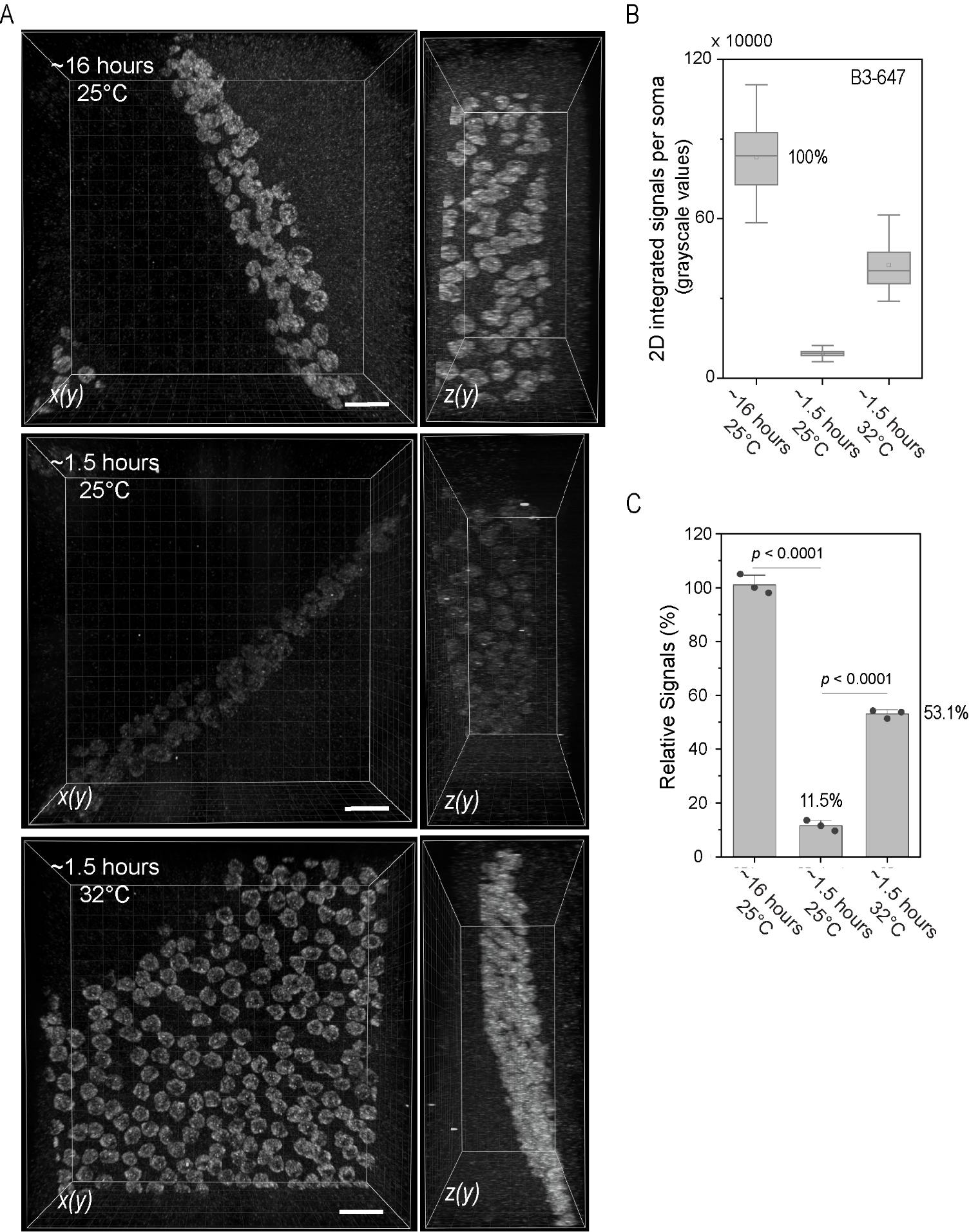


**fig. S5. Optimizing amplification temperature for cycleHCR.**

(A) To optimize the cycleHCR protocol, we evaluated the impact of different amplification temperatures and durations on signal performance using a cerebellar section of approximately 200 µm thickness. *Rgs8* mRNA was repeatedly stripped and reprobed under various temperature conditions to assess signal intensity variations. Imaging was performed to document signal differences across these conditions. 3D Image volumes are rendered by Imaris using MIP mode with the same intensity range for all images. Scale bars: 50 µm.

(B) Comparative analysis revealed that HCR amplification at 32°C for 1.5 hours achieved signal intensities about 51% of those obtained through overnight amplification at ambient room temperature (~25°C). In comparison, a 1.5-hour amplification at 25°C resulted in only approximately 11% of the signal intensity relative to the overnight conditions. Signal quantification involved integrating signals above background levels per cell in 2D slices, with approximately 50 quantifications conducted. The upper and lower whiskers represent 90% and 10% values; the box represents the range from 25% to 75% percentile; the center line represents the median; the dotted line indicates the mean.

(C) Statistics from 3 biological repeats with the same measurement as shown in (B). The average intensity from each field is normalized to the value shown in column 1 in (B) as 100%. *p*-values were calculated with ANOVA Bonferroni tests.

**
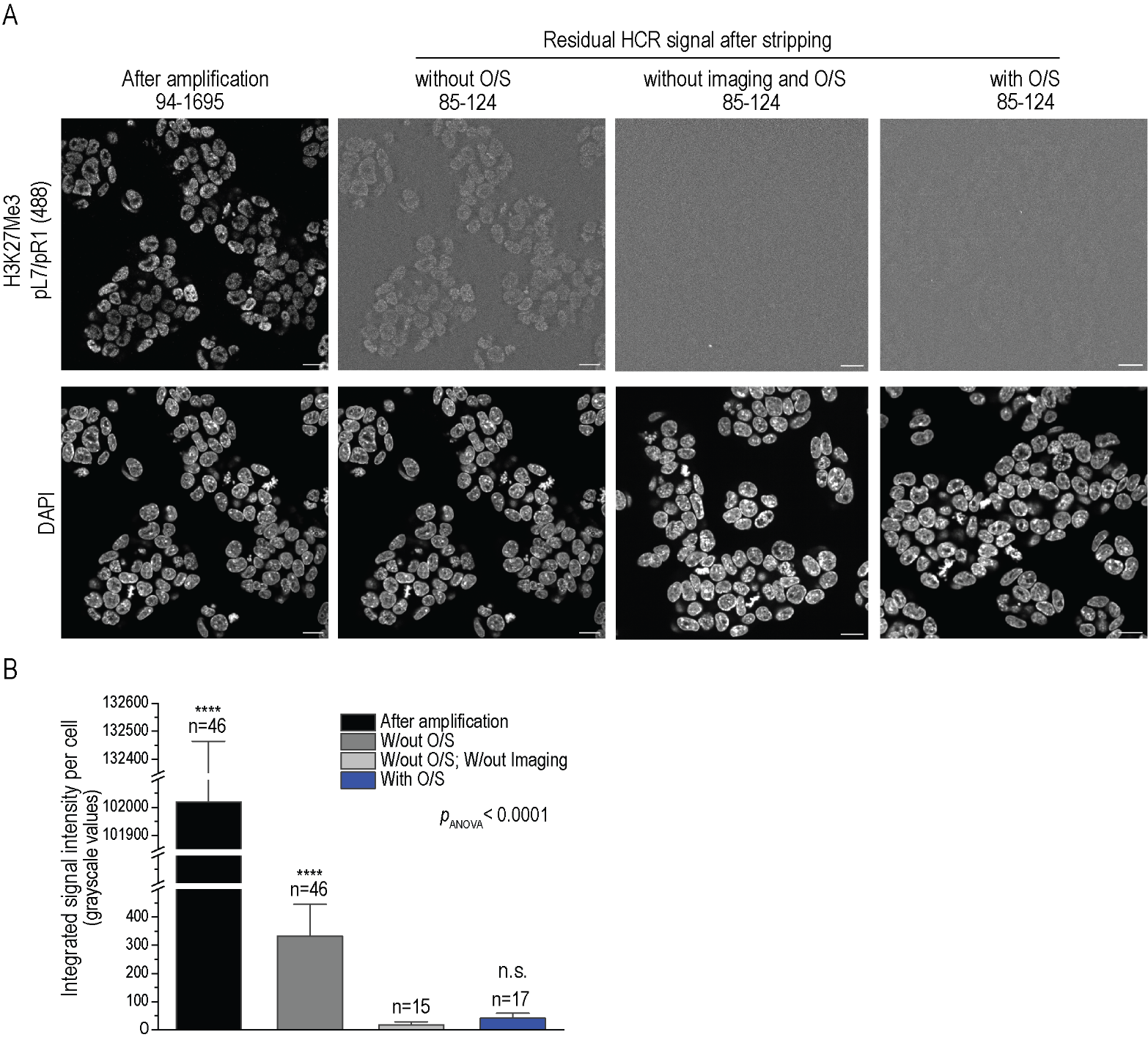
**

**fig. S6. Mitigating photo-crosslinking in cycleHCR imaging using oxygen scavenger.**

**(A) To evaluate photo-crosslinking effect,** samples following protein cycleHCR targeting histone H3K27me3 underwent imaging at 100% laser power. Then, continuous illumination for 15 seconds was then applied both with and without the presence of oxygen scavenger (O/S) to determine its effect on photo-crosslinking of HCR fluorescent probes to the specimen. Fluorescence images captured after probe stripping allowed for the assessment of photo-crosslinking levels. The presence of O/S substantially reduces photo-crosslinking. The display range for these images is provided above. Scale bars: 20 µm.

**(B)** The impact of O/S on reducing photo-crosslinking was quantitatively evaluated by measuring residual fluorescence signals exceeding background levels per cell in 2D slices. The analysis confirms the effectiveness of oxygen scavenger in minimizing undesired photo-crosslinking effects. The error bars represent standard deviations. A one-way ANOVA was conducted to assess significant differences across all groups, followed by pairwise *p*-values calculated using Bonferroni tests, with the no imaging and O/S condition serving as the reference. n.s., non-significant (*p* > 0.05); ****, *p* < 0.0001.


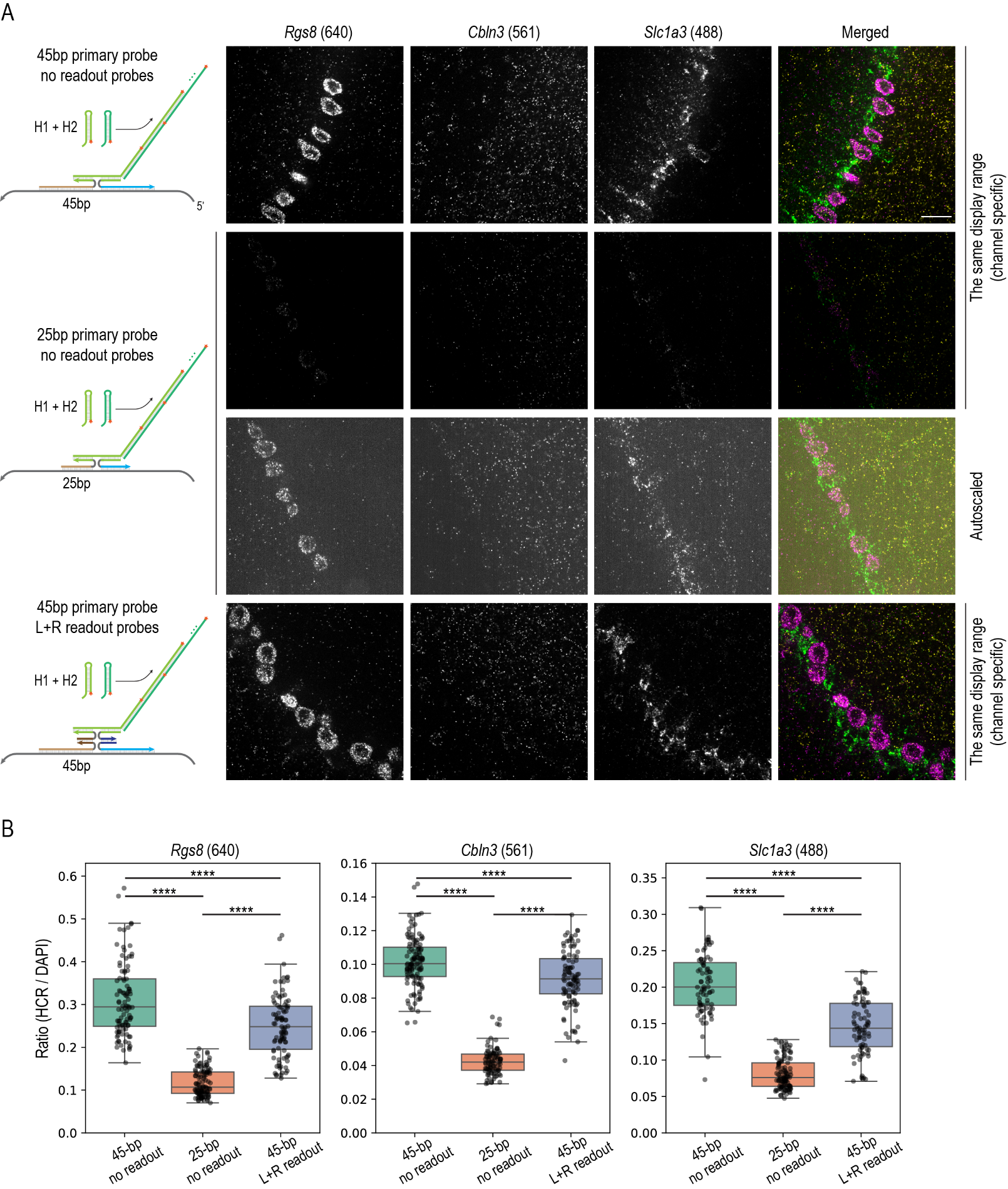


**fig. S7. Evaluation of probe length and barcode addition.**

(A) To assess the impact of increased probe length and the addition of the L + R barcode, we compared three conditions using cerebellar sections of approximately 200 µm thickness: (top row) 45-bp primary probe without barcode, (middle two rows) 25-bp primary probe without barcode, and (bottom row) 45-bp primary probe with L + R barcode. The third row shows autoscaled images for the 25-bp primary probe condition only. The center locations for split probe dock sites are identical across all 3 conditions. A stringent hybridization buffer (50% deionized formamide, 10% Dextran Sulfate, 2X SSC) was used with a normalized final concentration of approximately 1 nM per primary probe. The arrowhead indicates the 3’ end of RNA or DNA. Scale bar: 25 µm.

(B) Statistical analysis was performed from multiple regions of interest (ROIs) by comparing the HCR signal to the nuclear DAPI signal per ROI, normalizing HCR signals to the number of cells. We used cells located at similar image depths (~100 µm) for comparison. A one-way ANOVA followed by Bonferroni tests was performed. ****, *p* < 0.0001.


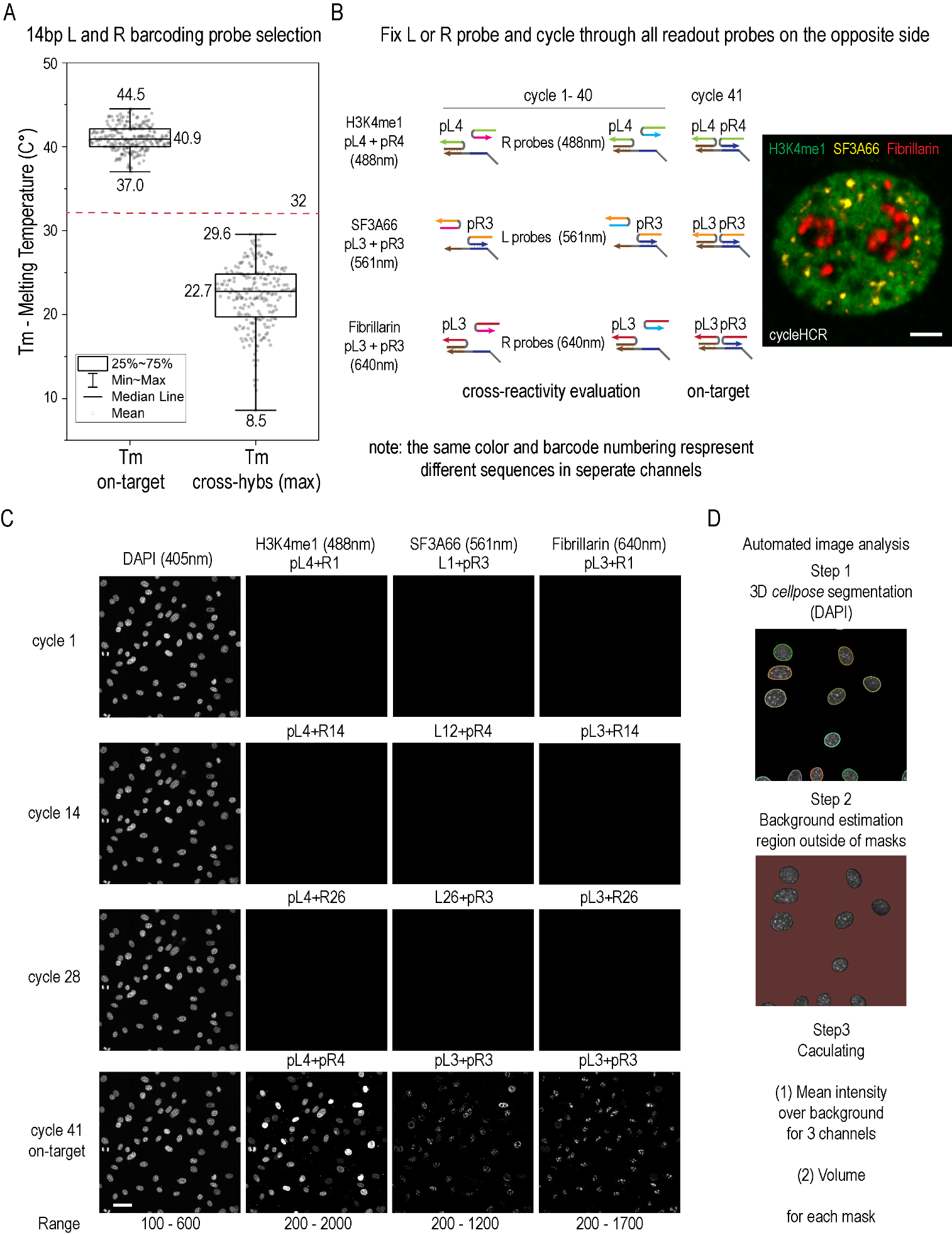


**fig. S8. L + R barcode cross-reactivity evaluation.**

(A) Melting temperatures (Tm) for on-target and cross-hybridization of 230 14-bp L and R barcoding probes. The maximum cross-hybridization Tm is calculated among probes sharing the same split initiator sequence. The arrowhead indicates the 3’ end of DNA.

(B) Evaluation of cross-reactivity among barcodes using abundant nuclear protein targets (H3K4me1, SF3A66, and fibrillarin) in NIH/3T3 cells. The L barcode was consistent for H3K4me1 and fibrillarin, and the R barcode was consistent for SF3A66. The test involved cycling through all off-target barcodes on the opposite side to assess cross-reactivity in the first 40 cycles, with the final cycle demonstrating on-target detection. A zoom-in composite cycleHCR image is shown on the right. Scale bar: 5 µm.

(C) Representative images from indicated cycles for all 4 channels. Scale bar: 50 µm.

(D) Automated imaging analysis pipeline for 3D cell segmentation and calculation of mean intensity above background and nuclear volume for each mask.


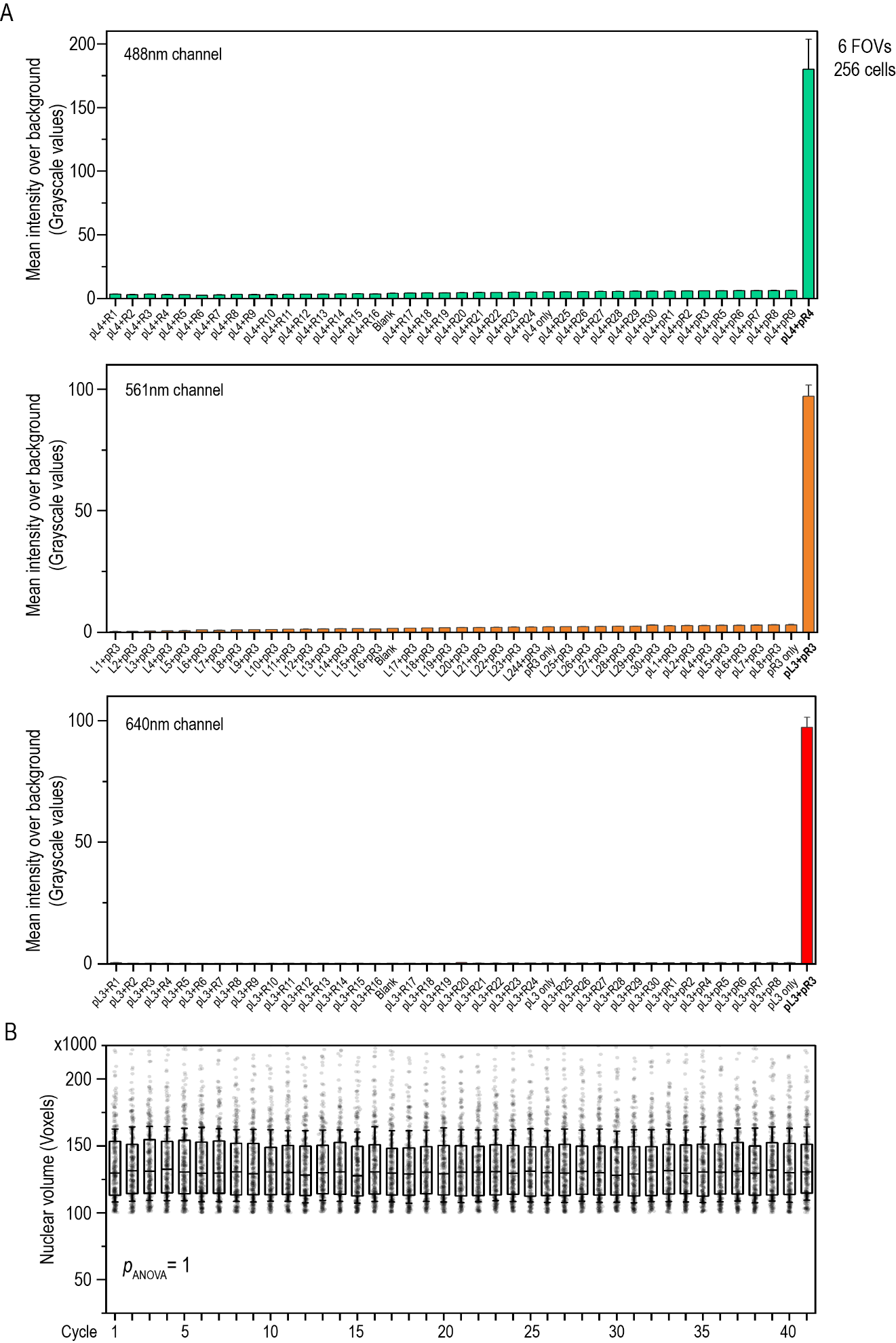


**fig. S9. cycleHCR barcode cross-reactivity evaluation.**

(A) Mean intensity over background within nuclear masks for 256 cells across 6 fields of view (FOVs) over 41 cycles. Error bars represent 95% confidence intervals. Negligible barcode cross-talks were detected for off-target barcodes across all 3 channels. Nonspecific fluorescence background increased approximately 1.7% and 2.8% for the 488 nm and 561 nm channels, respectively, while the background for the 640 nm channel remained stable throughout all cycles. This suggests that background increases are likely attributable to autofluorescence. The error bars represent 95% confidence interval.

(B) Total nuclear volume, measured in number of voxels, for 256 cells across 6 field of views (FOVs) across all 41 cycles. The lower and upper whiskers represent standard deviation (SD); the box shows the range from the 25th to the 75th percentile; the center line represents the median. A one-way ANOVA was conducted to assess significance of differences across all groups.


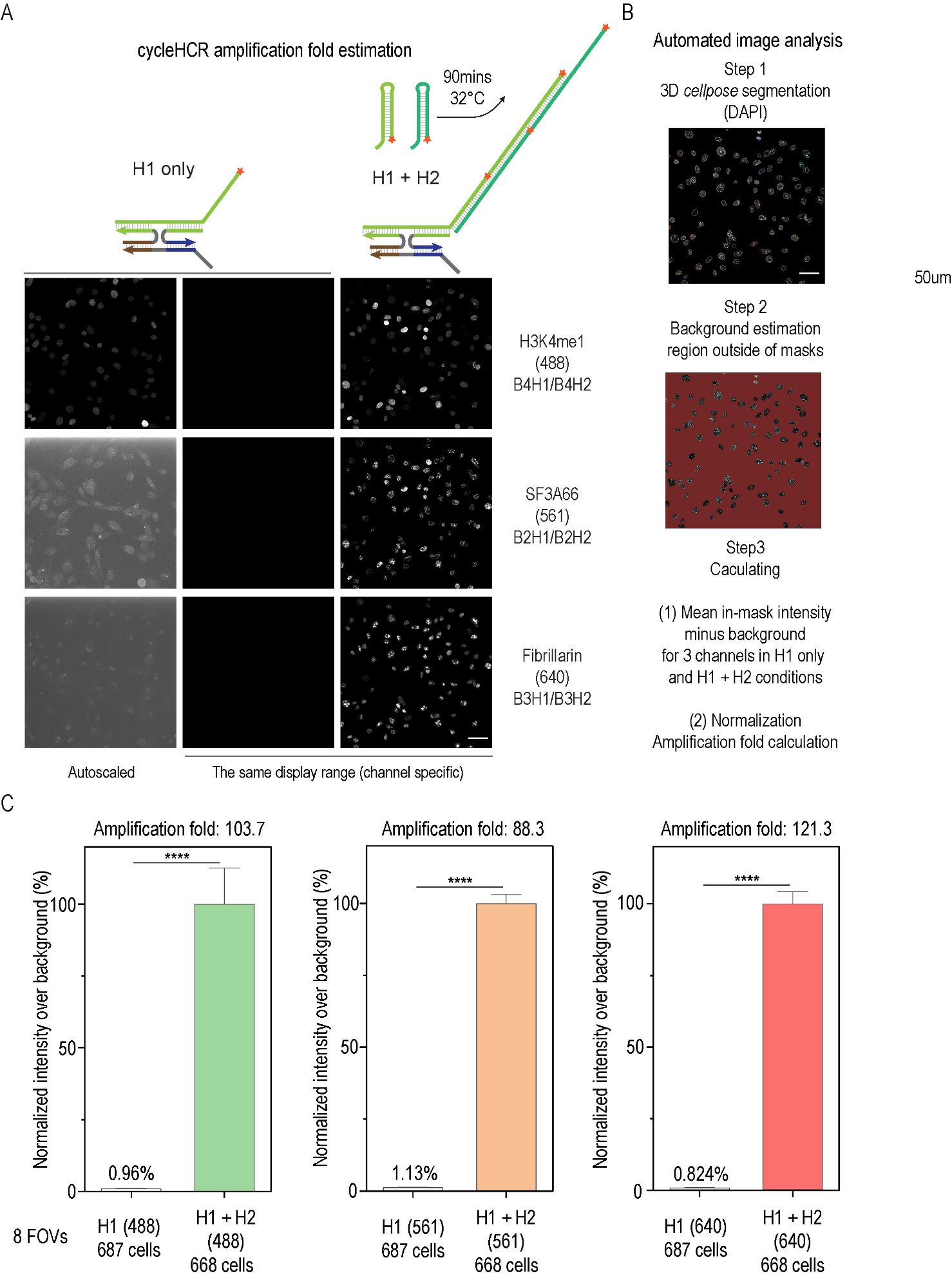


**fig. S10. Amplification fold estimation across 3 color channels**

(A) To estimate the amplification fold across 3 color channels, we compared conditions without amplification (H1 only) and with typical amplification conditions (H1 + H2, 32°C, 90 min) for gel-anchored cycleHCR barcodes targeting H3K4me1 (488 nm), SF3A66 (561 nm), and Fibrillarin (640 nm), used in protein cycleHCR as shown in fig. S8. The left column shows autoscaled images of the H1 only condition. Middle and right columns display images of both H1 only and H1 + H2 conditions with identical range settings. Arrowheads indicate the 3’ end of DNA.

(B) Automated imaging analysis pipeline for 3D cell segmentation and calculation of mean intensity above background for both H1 only and H1 + H2 conditions.

(C) The bar graph represents intensity over background, normalized to the amplification condition (H1 + H2). The number of cells and the field-of-views (FOVs) analyzed under each condition is indicated below each bar. Error bars represent the 95% confidence interval. Significance was calculated using a t-test: ****, p < 0.0001. Calculated amplification fold for each channel is annotated on the top the graph.

Scale bars: 50 µm.

**
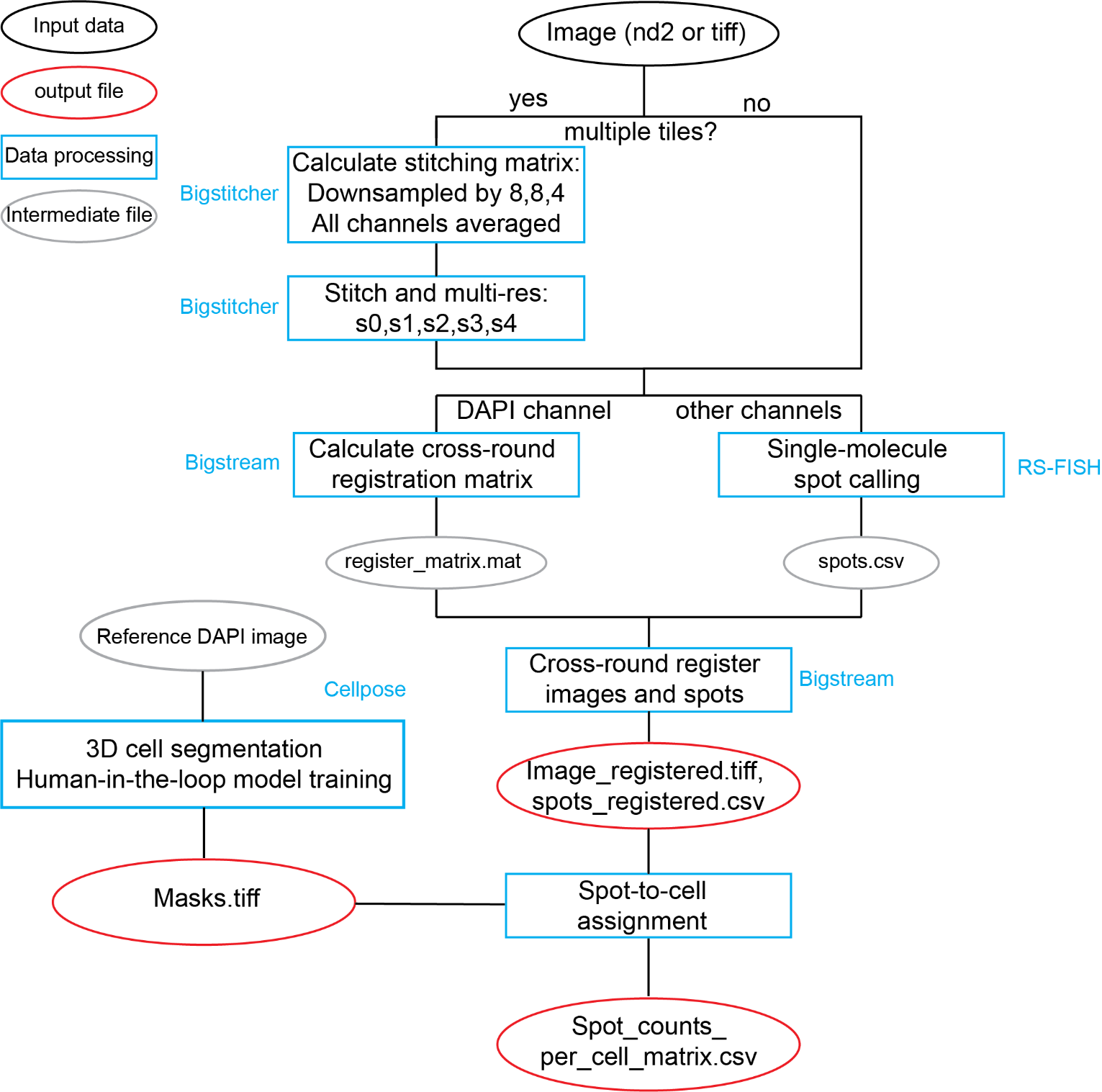
**

**fig. S11. Nextflow orchestrated image processing workflow for cycleHCR.**

This figure presents a workflow designed for efficient processing of imaging data. The workflow is orchestrated with Nextflow and includes the following steps: 1) **Initial categorization:** raw image files, in Nd2 and Tiff formats, are first categorized based on the number of tiles present at each time point. 2) **Image stitching:** for instances where the number of tiles exceeds one, images undergo stitching via BigStitcher. Conversely, single-tile images bypass this stitching step. 3) **RNA localization:** RS-FISH is utilized to identify mRNA single molecule localizations 4) **Cross-round registration:** The BigStream tool calculates cross-round registration matrices, enabling the alignment of images and localization spots across different rounds using DAPI channel data for consistent registration. 5) **Cell segmentation:** cell segmentation is conducted using Cellpose using the reference DAPI channel, using a custom model refined through a human-in-the-loop method to ensure accurate segmentation of nucleus boundaries. 6) **Spot-to-cell assignment:** following registration, localizations are assigned to each segmented cell, culminating in a comprehensive localization count per cell for all identified cell masks within the image.


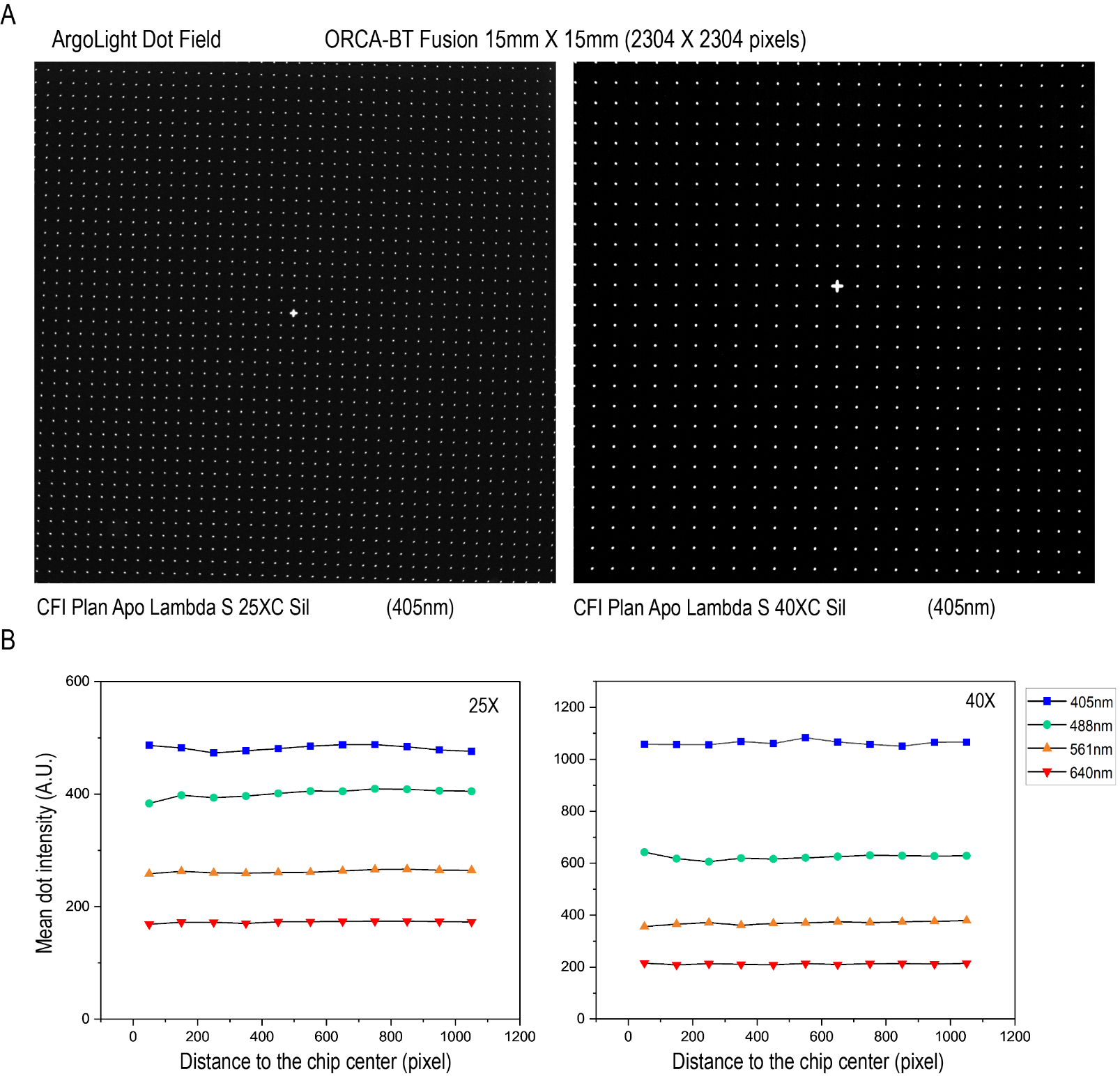


**Fig. S12. Evaluation of laser illumination uniformity.**

(A) Representative full chip images of the ArgoLight dot field obtained using 25x and 40x silicone oil immersion objectives with 405 nm laser illumination.

(B) Illumination uniformity was evaluated by measuring and calculating the average dot intensity, plotted as a function of distance from the camera chip center, for different laser wavelengths: 405 nm, 488 nm, 561 nm, and 640 nm.

Flat illuminations are critical for accurate image stitching across tiles.


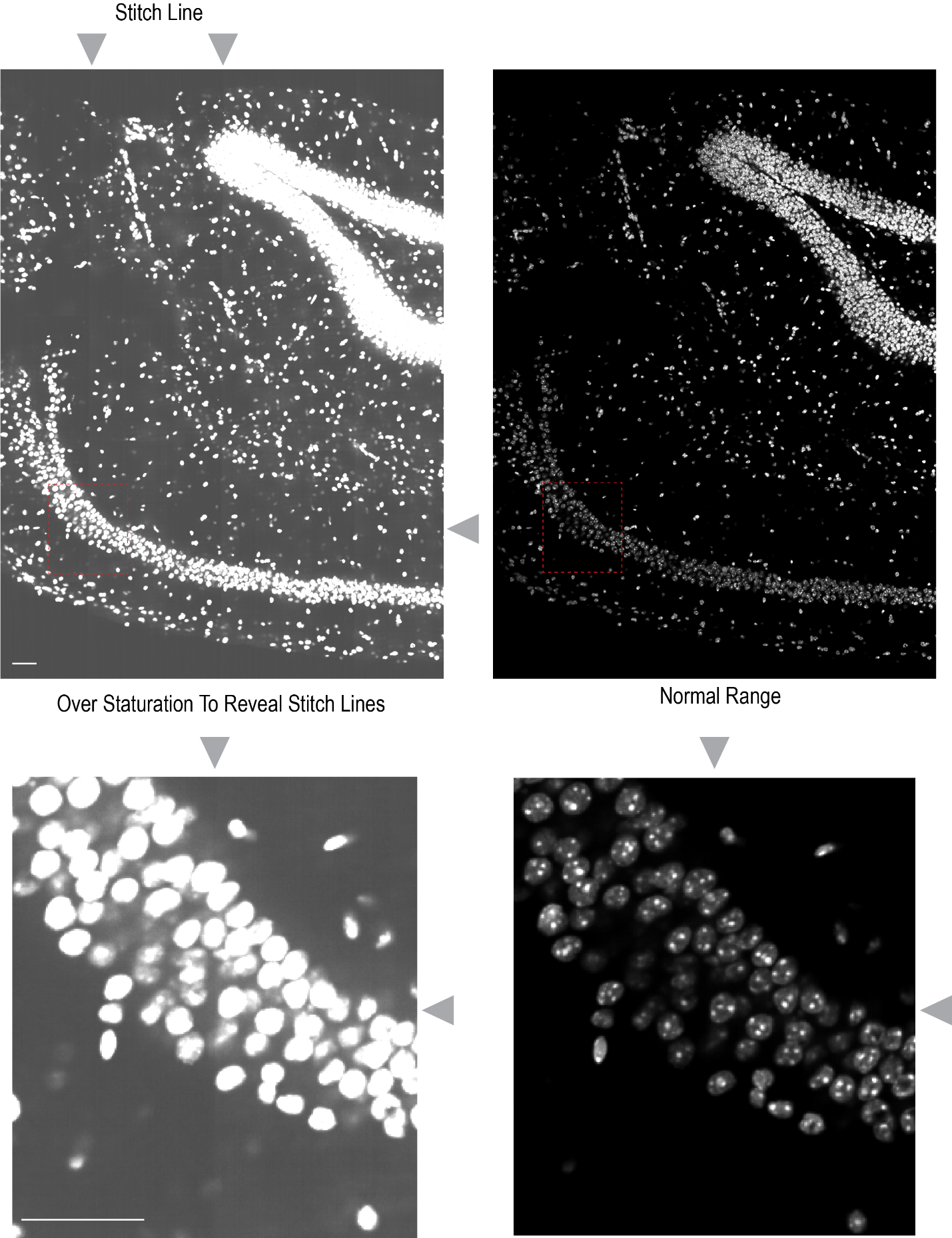


**fig. S13. Typical stitching performance in cycleHCR image processing workflow.**

Zoom-in images of hippocampal data to demonstrate typical image stitching in the workflow: (top-left) under over-saturation conditions revealing stitch lines and (top-right) within a normal range. The zoom-in image of the stitched region is shown on the bottom. Scale bar: 50 µm.

**
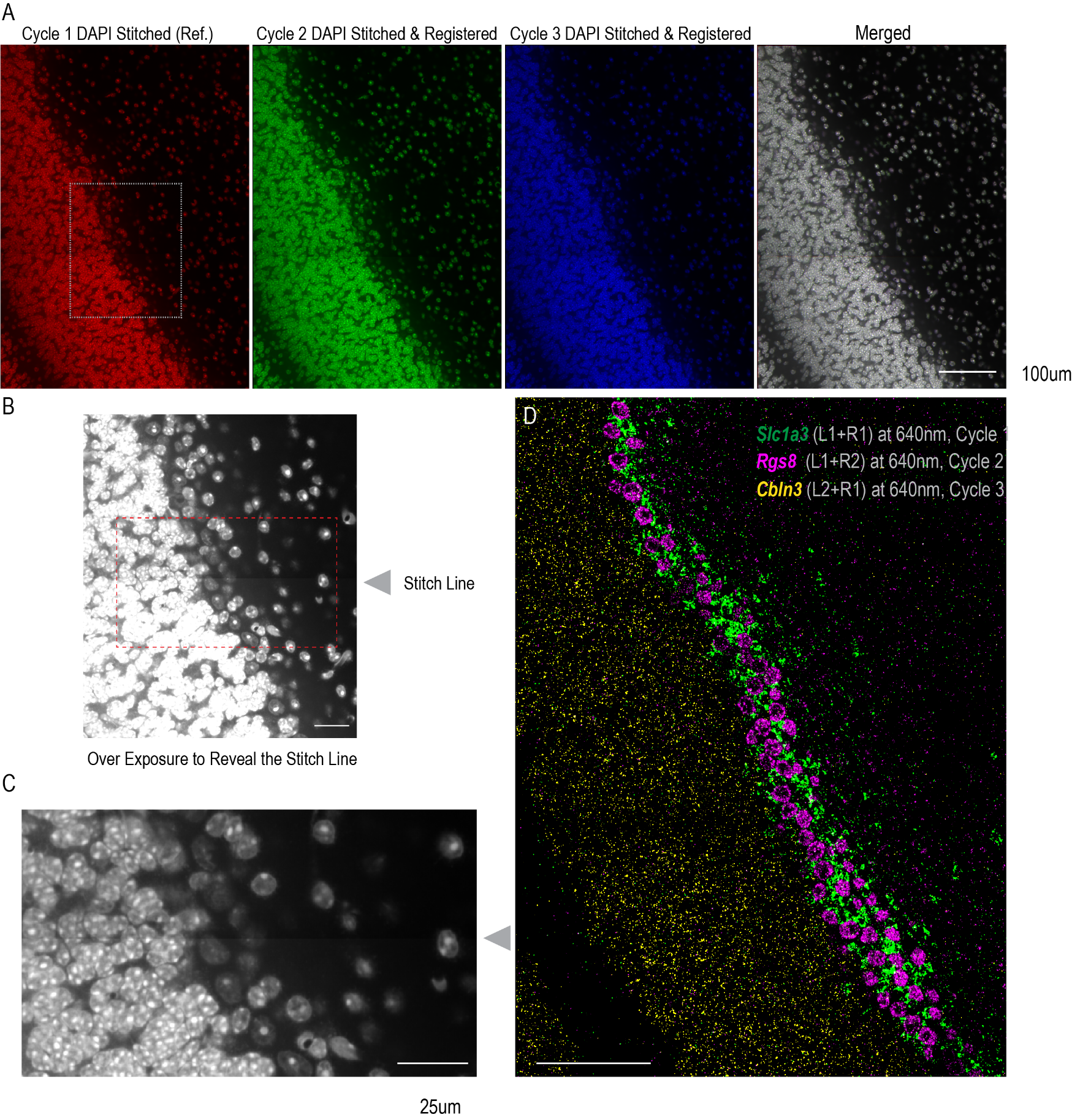
**

**fig. S14. Cross-round registration validation in mouse cerebellar slice imaging.**

(A) Registration validation of post-stitching alignment. The reference DAPI image (red) is overlaid with DAPI images from Cycle 2 (green) and Cycle 3 (blue) for sequential cycles in cycleHCR, as shown in Fig. 1E. Scale bar: 100 µm.

(B) Close-up view of the marked box in (A) with overexposure to reveal the stitch line indicated by the gray triangle. Scale bar: 25 µm.

(C) Close-up view of the same box in (B) with normal display range to evaluate stitching performance across the stitch line. Scale bar: 25 µm.

(D) Sequential cycleHCR RNA signals for three genes: *Slc1a3* (L1 + R1 at 640 nm), *Rgs8* (L1 + R2 at 640 nm), and *Cbln3* (L2 + R1 at 640 nm) across three cycles. Scale bar: 100 µm.


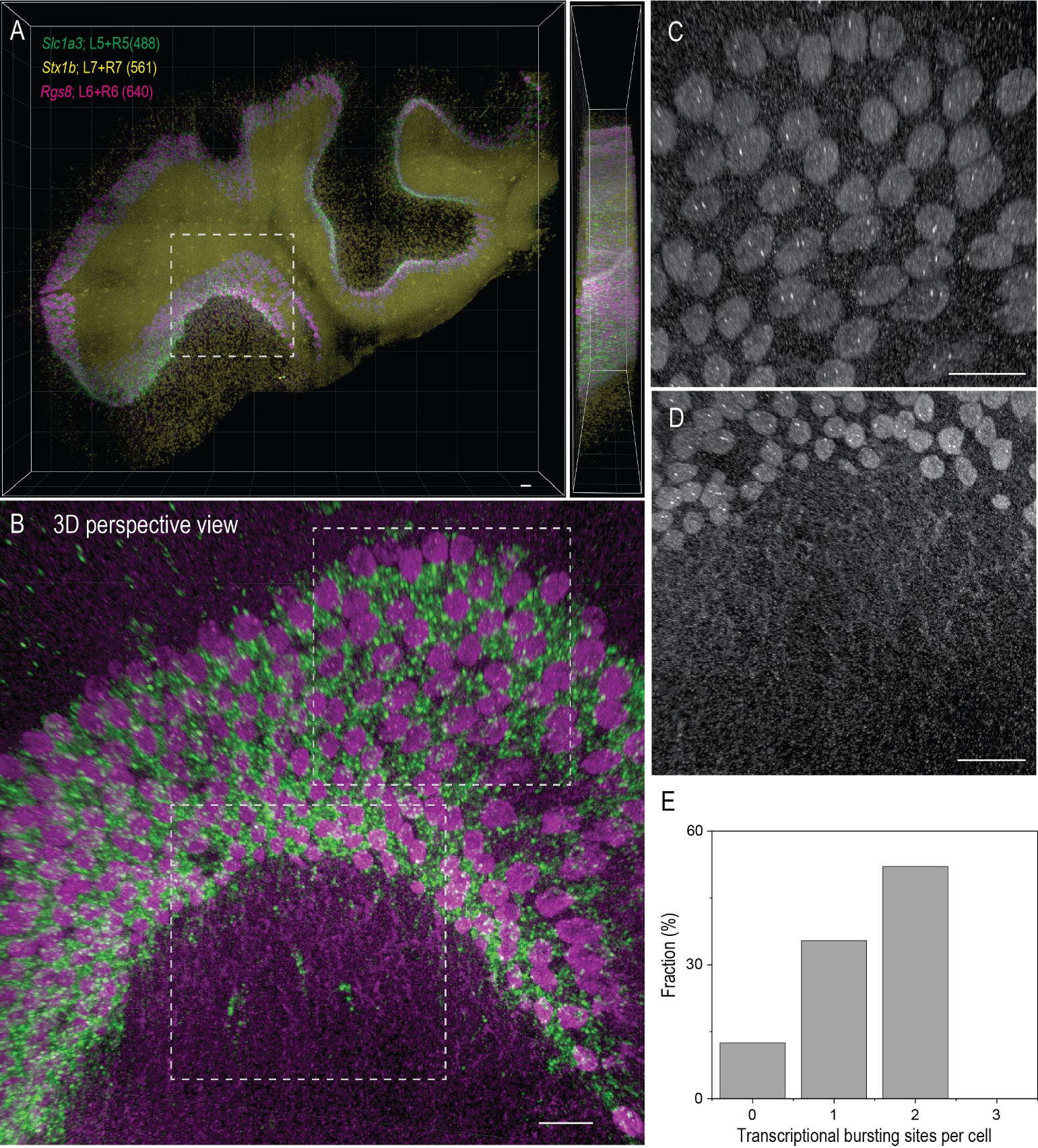


**fig. S15. Detailed sub-cellular RNA imaging in thick cerebellum slice via cycleHCR.**

**(A)** Using a three-color, single-round cycleHCR approach, we visualized RNAs from three genes specific to different cell types within a cerebellum slice measuring 2.4 mm x 1.8 mm x 0.2 mm. The genes imaged include *Slc1a3* (L5 + R5 at 488 nm) for Bergmann glia cells, *Stx1b* (L7 + R7 at 561 nm) for the granule layer, and *Rgs8* (L6 + R6 at 640 nm) for Purkinje cells. This panel shows the capability of cycleHCR to delineate gene expression patterns across cell types in thick tissue sections.

**(B)** A three-dimensional perspective view of *Slc1a3* (green) and *Rgs8* (magenta) RNA localizations within a specified region from (A).

**(C)** cycleHCR reveals transcriptional bursting sites of the *Rgs8* gene within *Purkinje* cells, visible in a designated upper box region in (B).

**(D)** *Rgs8* mRNA molecules localize along the dendrites of Purkinje cells, as shown in the lower box region in (B). This detail emphasizes the ability of cycleHCR to map mRNA distributions within specific subcellular compartments.

**(E)** Statistical analysis of the number of transcriptional bursting sites of *Rgs8* gene (Chr 1) per cell across populations of Purkinje cells, with approximately 200 cells quantified.

Images are rendered using the 3D perspective view of maximal intensity projection (MIP) with Imaris.

Scale bars: 50 µm.


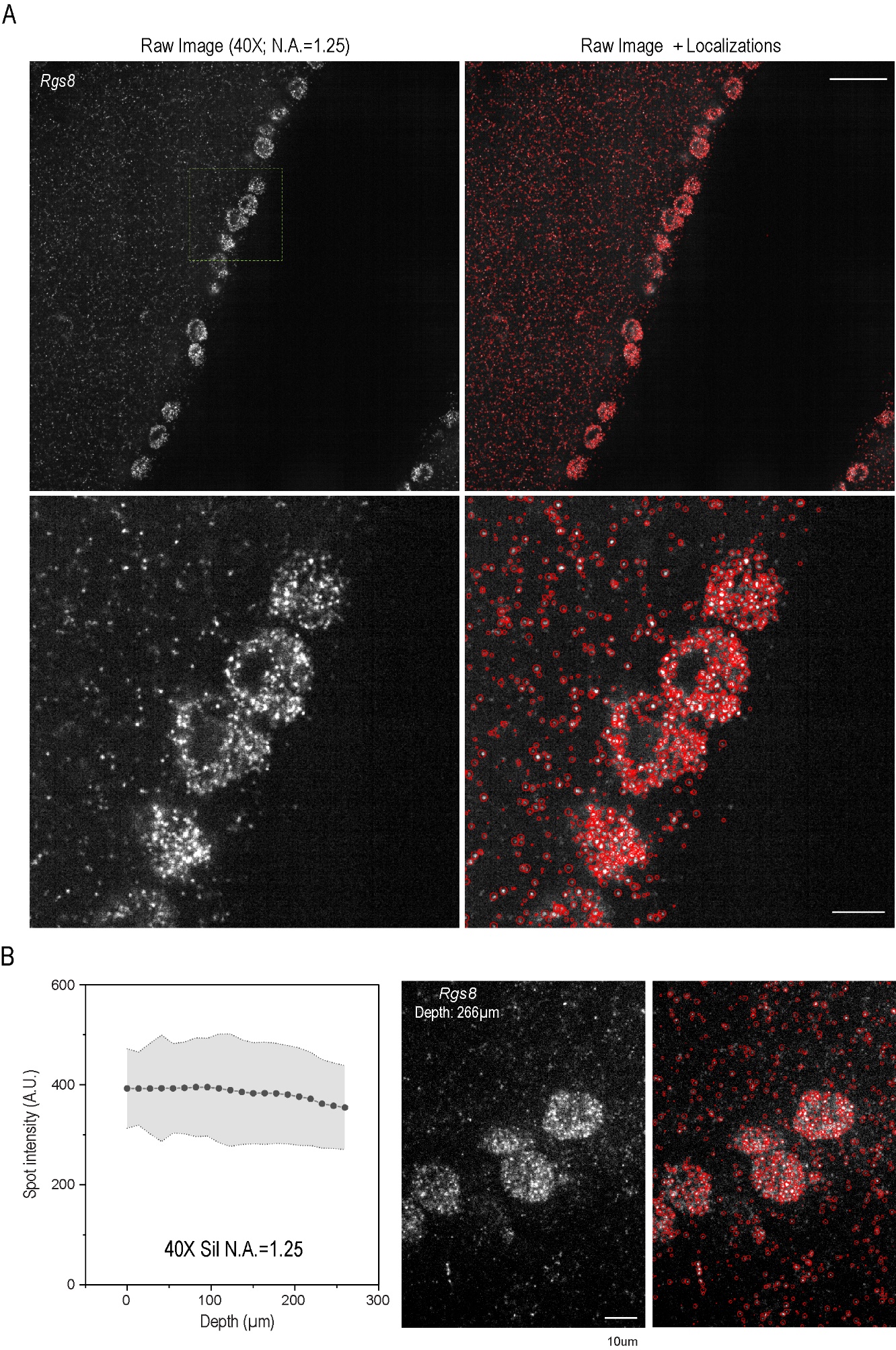


**fig. S16. Validation of cycleHCR RNA spots and RS-FISH localizations.**

(A) The image in fig. S15 was down-sampled and optimized for 3D rendering by Imaris. This figure further validates our ability to resolve and localize individual RNA spots. The upper left panel shows a full-frame raw slice image of *Rgs8* cycleHCR RNA spots in the cerebellar tissue section. The upper right panel displays RS-FISH spot detections marked with red circles overlaid on the raw image. The lower panels provide zoom-in views of the boxed region from the upper panel. Smaller red circles indicate the centers of these molecules are in other *z* slices. Scale bars: 50 µm (upper panels); 10 µm (lower panels).

(B) Evaluation of spot detection over axial depth using a 40X silicone objective. The left panel shows the relationship between spot intensity and depth, indicating relatively stable spot intensity up to 200 µm, with less than 10% reduction beyond 200 µm. The shaded region reflects standard deviation. The middle panel presents raw data acquired at a 266 µm depth, while the right panel displays spot detection marked with red circles overlaid on the raw image. Smaller red circles indicate the centers of these molecules are in other *z* slices. Scale bar: 10 µm

**
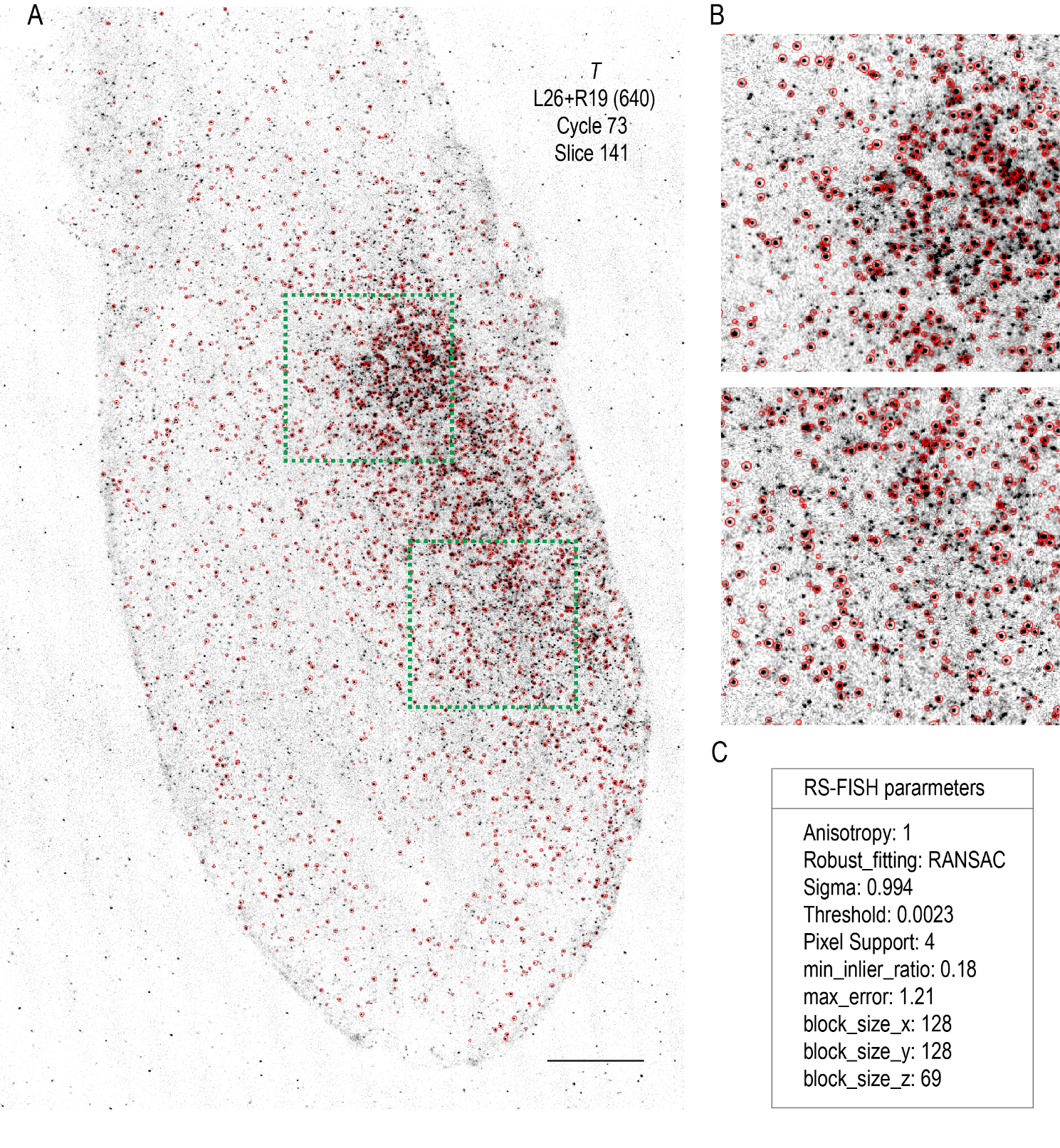
**

**fig. S17. Single-molecule localization for whole-embryo transcriptomics imaging.**

**(A)** This panel demonstrates the precision of single-molecule localization in imaging mRNA transcripts of *T* gene. Red circles denote the positions of localized single molecules detected by RS-FISH, overlaid on an inverted raw image captured using the cycleHCR barcode L26 + R19 at the 640 nm channel at cycle 73. Scale bar: 50 µm.

**(B)** A zoomed-in view of areas within green boxes from (A) provides a closer examination of the single-molecule localization specificity, highlighting the ability of cycleHCR in capturing transcriptomic details at the molecular level.

**(C)** Single-molecule localization parameters with RS-FISH for localizing single-molecules. These parameters were selected to minimize false positive detections.

**
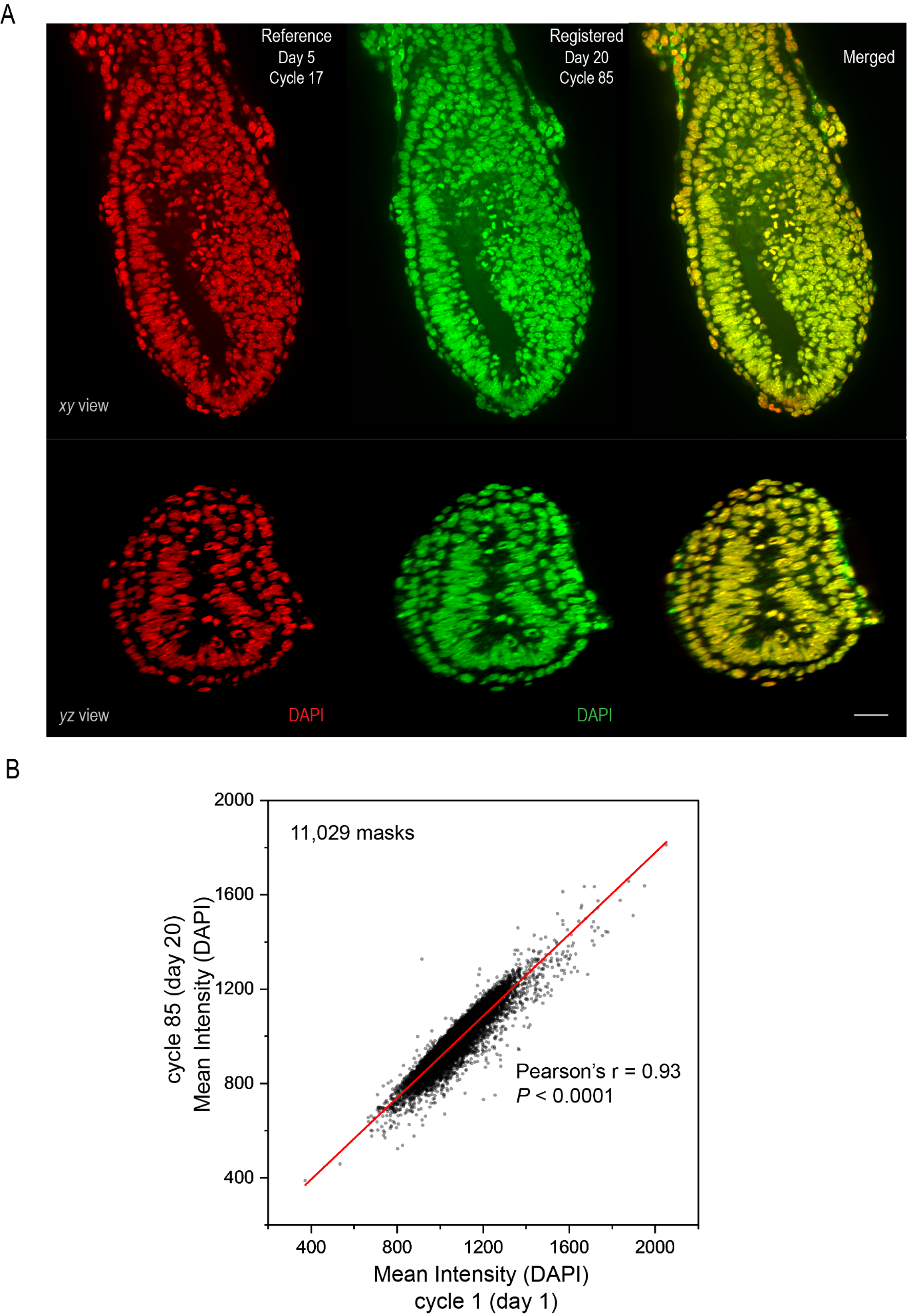
**

**fig. S18. Evaluation of sample deformation during cycleHCR imaging using the mouse embryo.**

**(A) Comparison of the reference DAPI image from Cycle 17 (red) with the image from the final Cycle 85 (green) in both *xy* and *yz* planes. The images demonstrate excellent alignment between disparate imaging cycles using only affine registration, indicating minimal sample deformation. Scale bar: 50 µm.**

**(B) Mean DAPI intensities for 11,029 segmented masks from Cycle 1 (Day 1) are highly correlated with mean DAPI intensities from Cycle 85 (Day 20).**

**
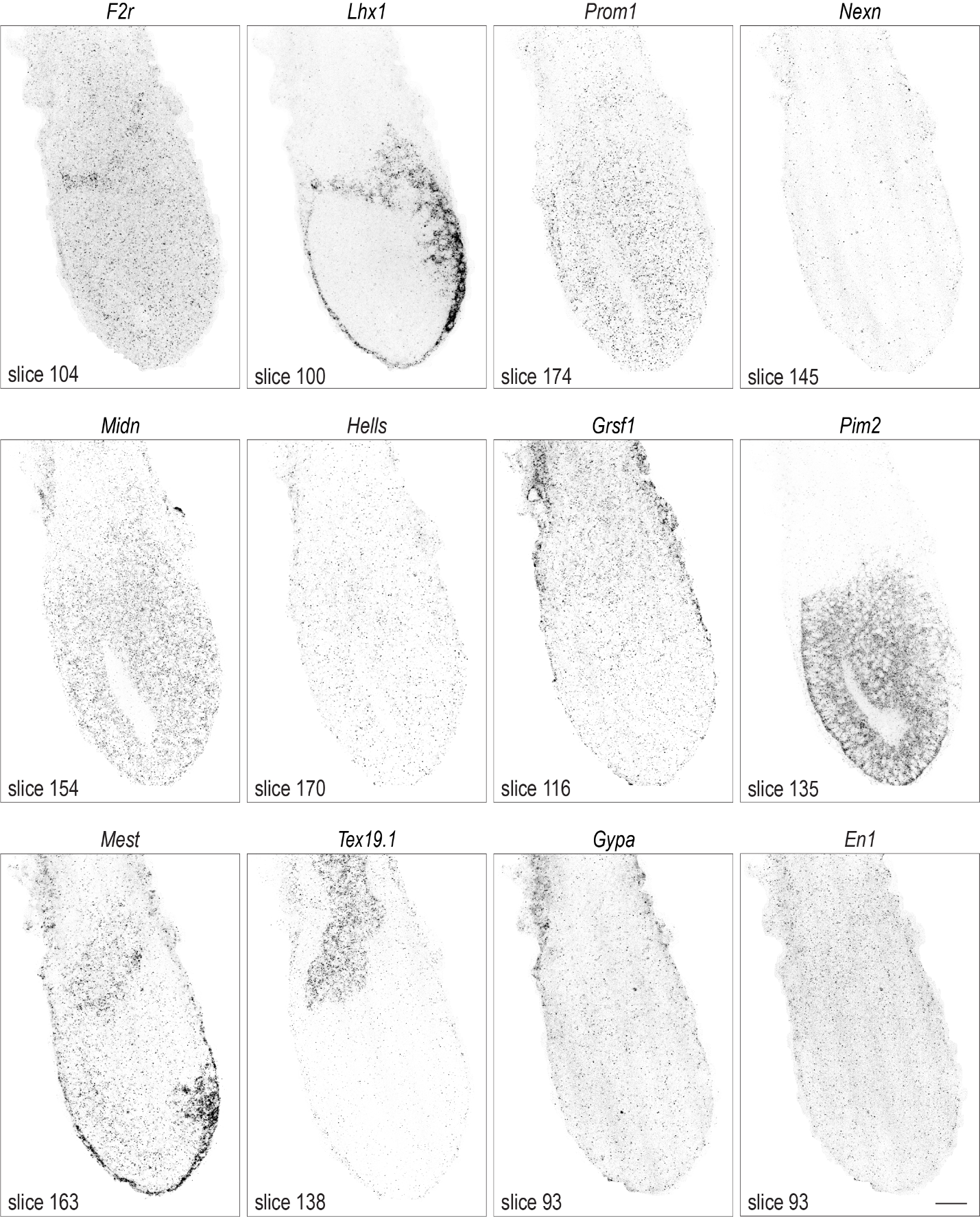
**

**
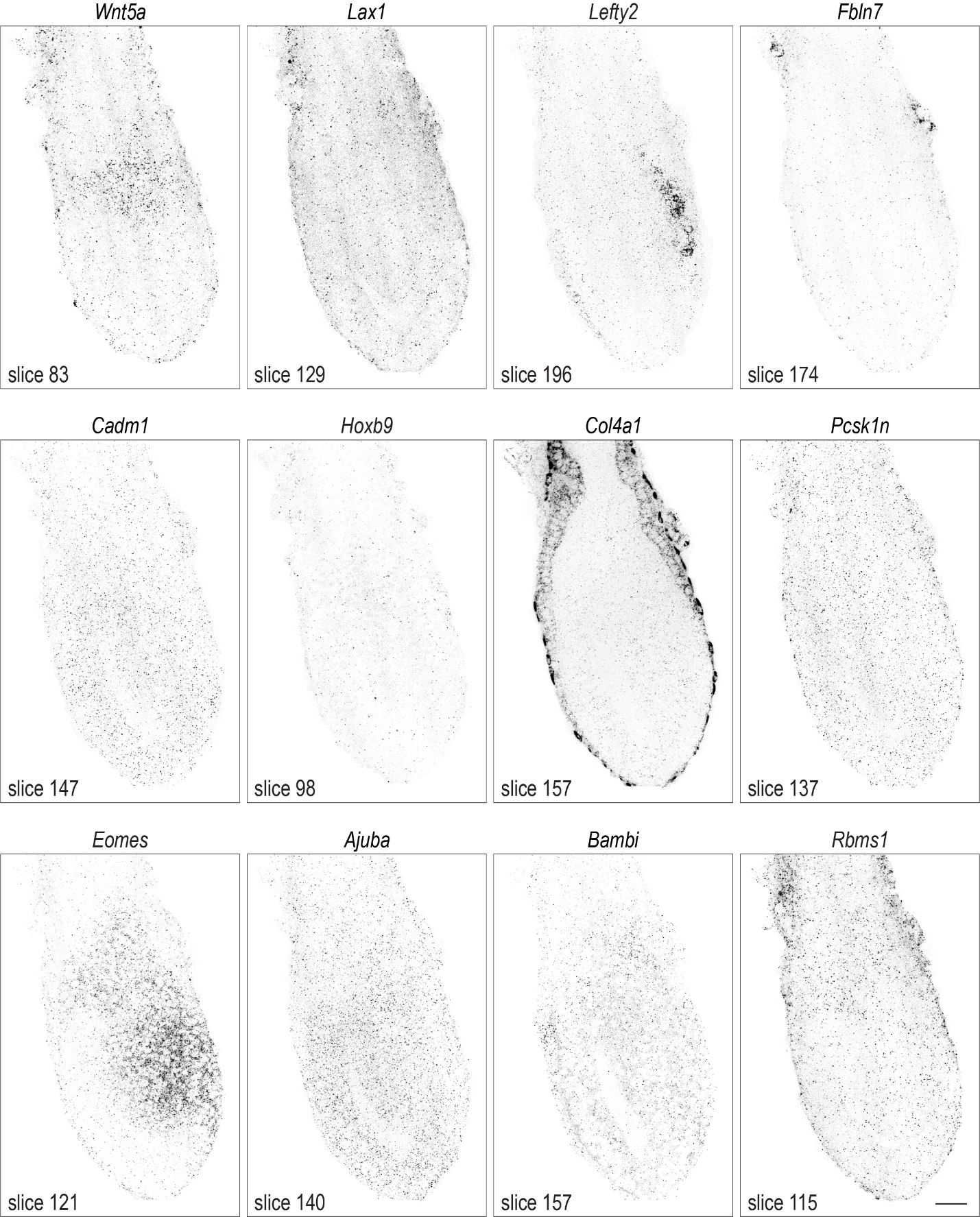
**

**
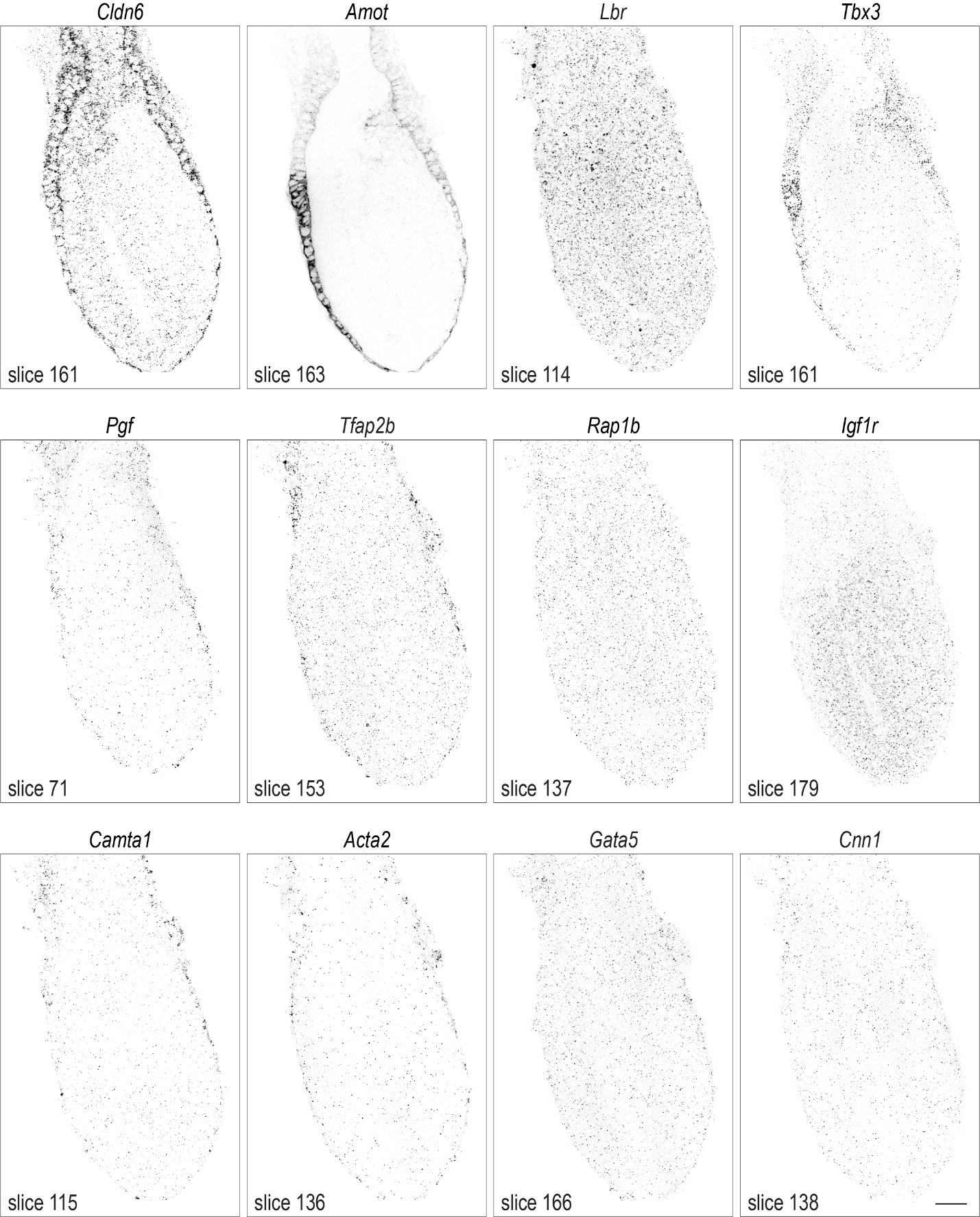
**

**
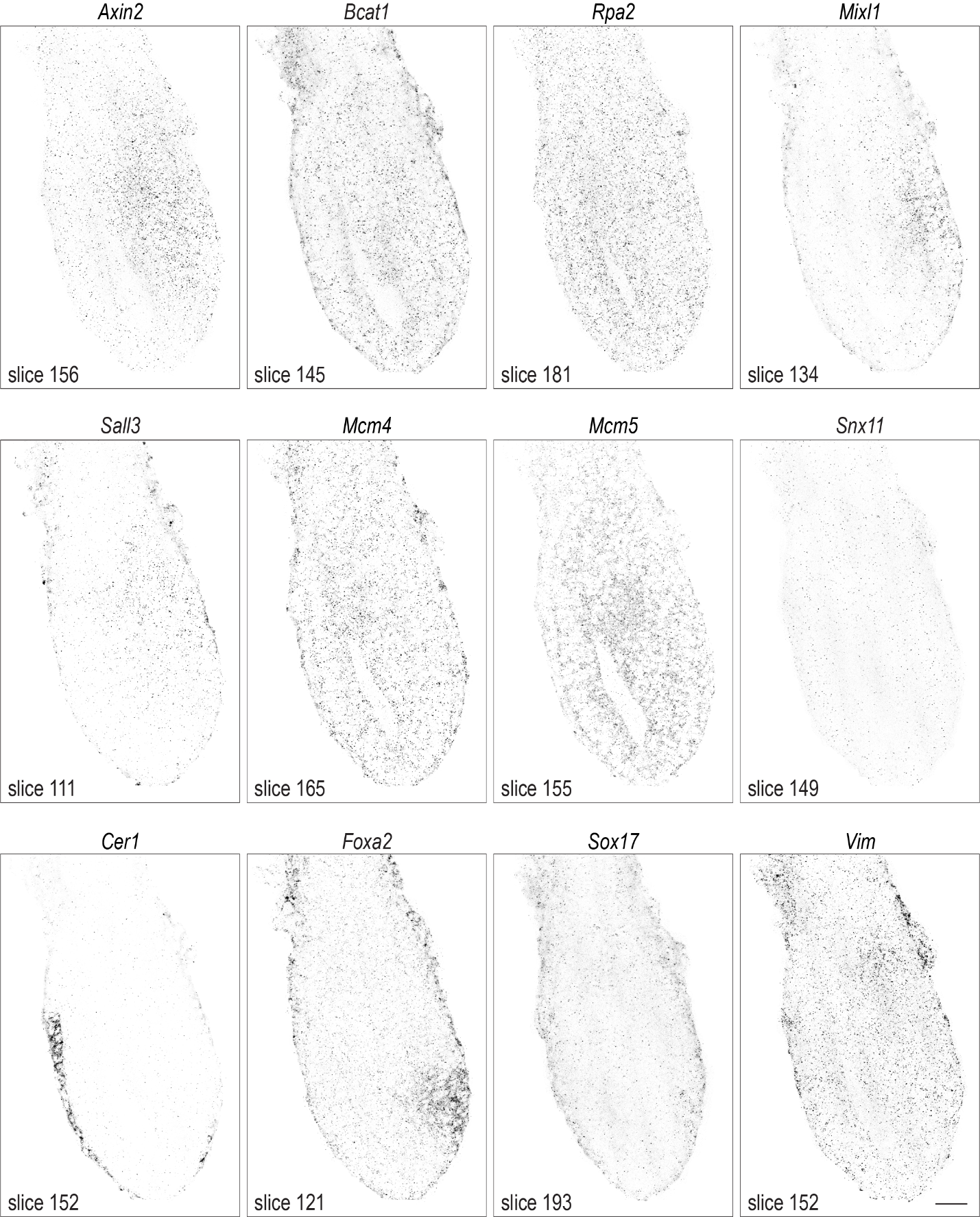
**

**
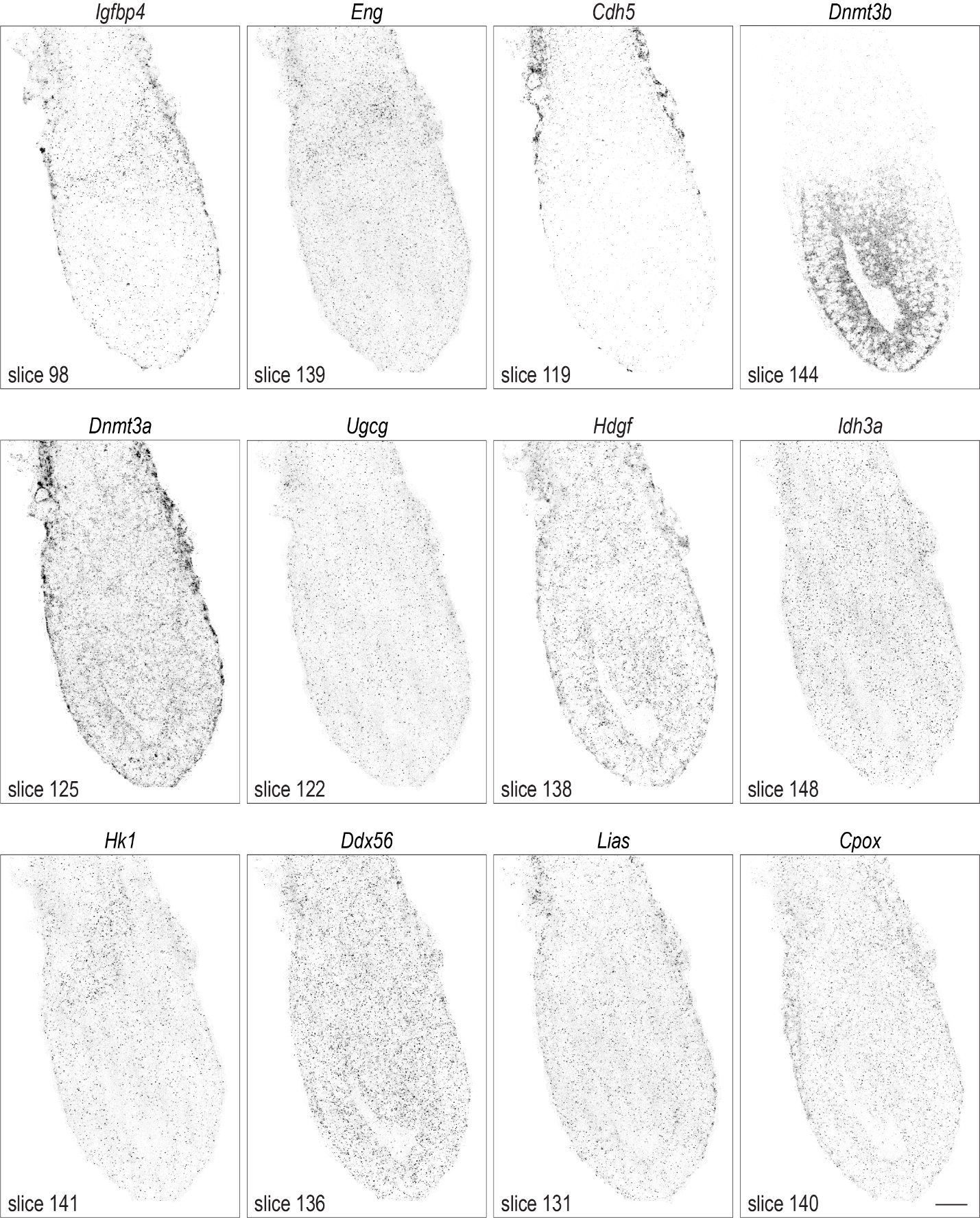
**

**
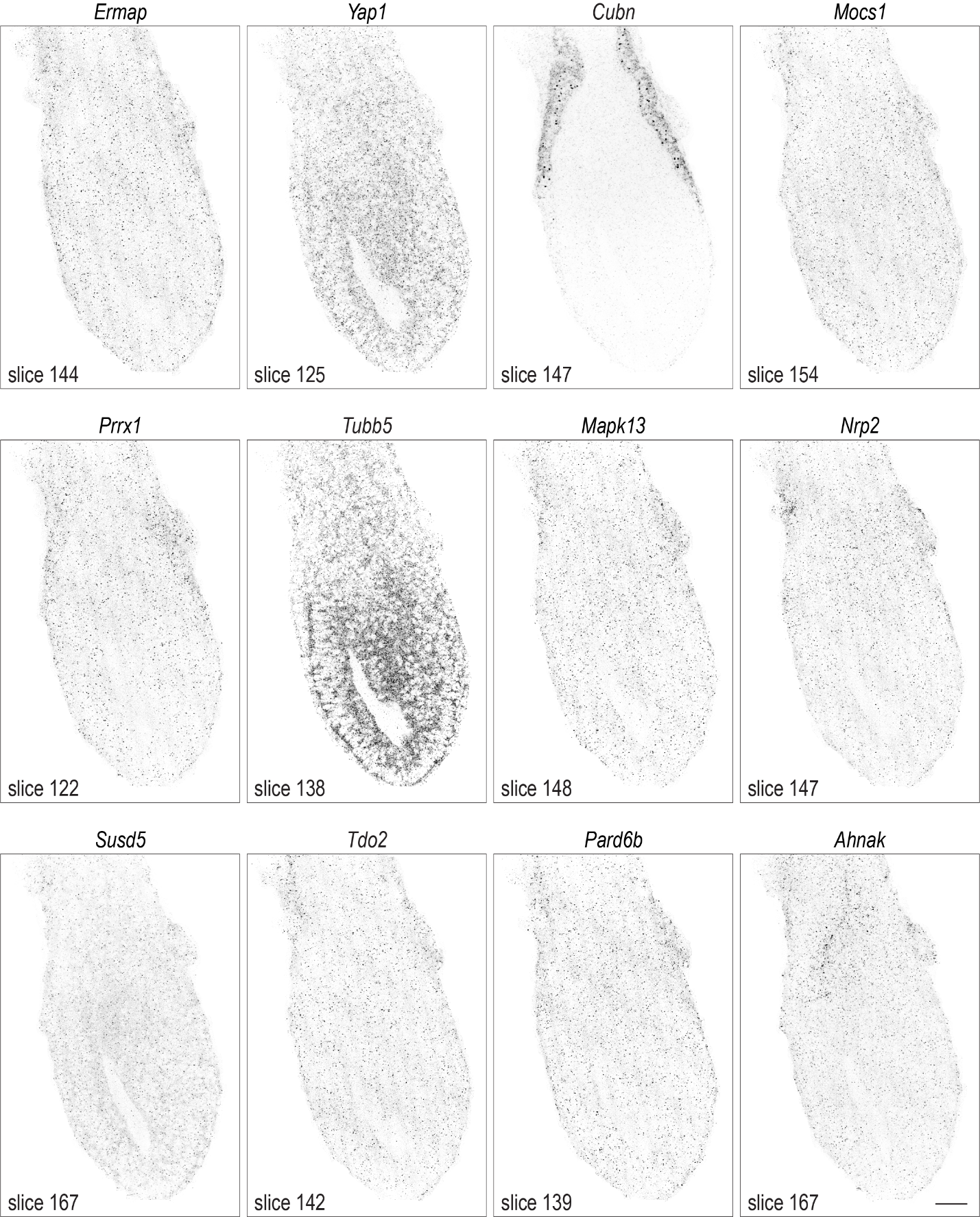
**

**
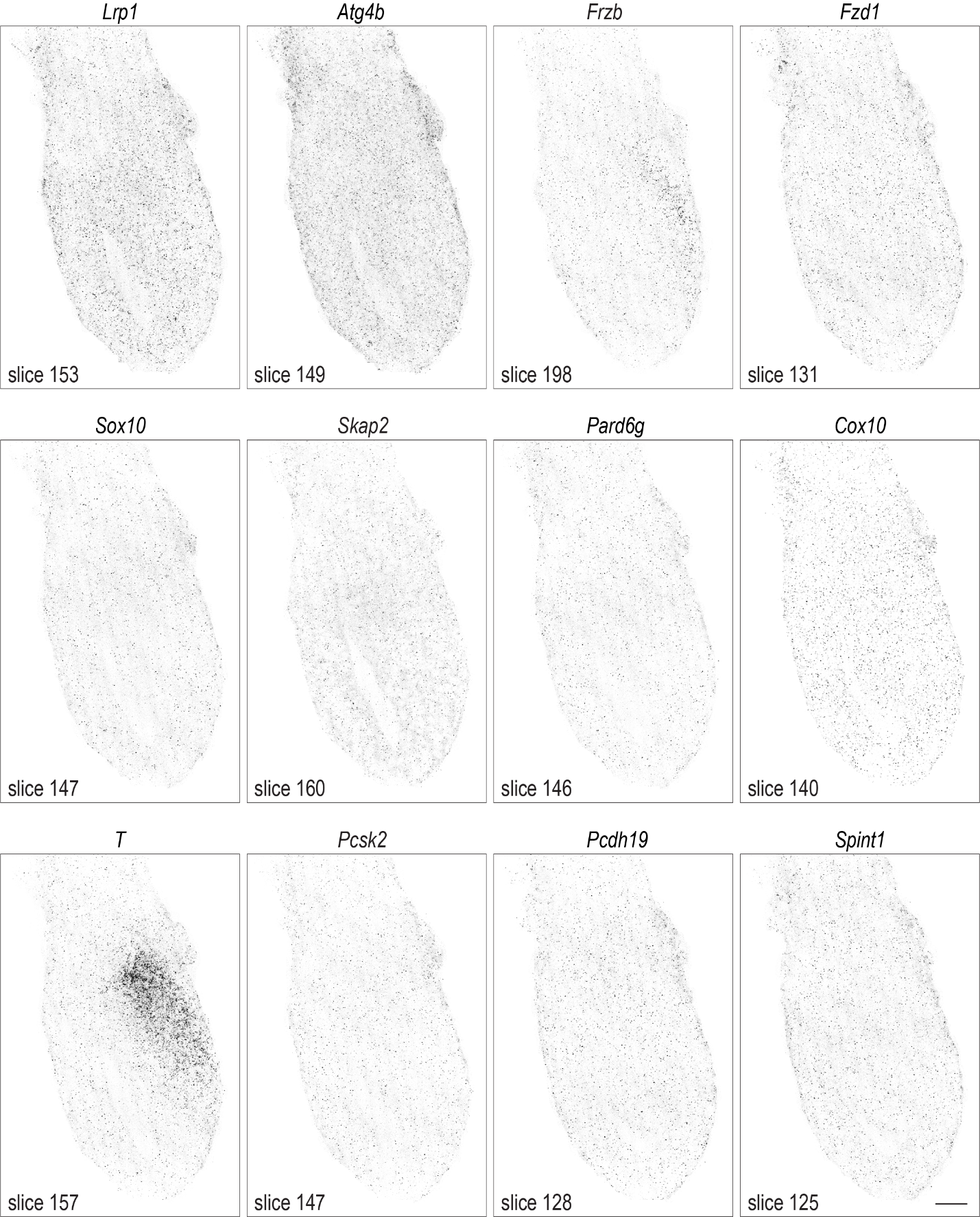
**

**fig. S19. Whole-embryo transcriptomics imaging.**

This figure features a series of inverted raw images captured during whole-embryo transcriptomics imaging as shown in Fig. 2, highlighting the mRNA expression patterns of indicated genes detected by the cycleHCR technique. Each subpanel displays a slice of the embryo, with the slice number indicated. The images are taken at 1 µm intervals. Scale bar: 50 µm.

**
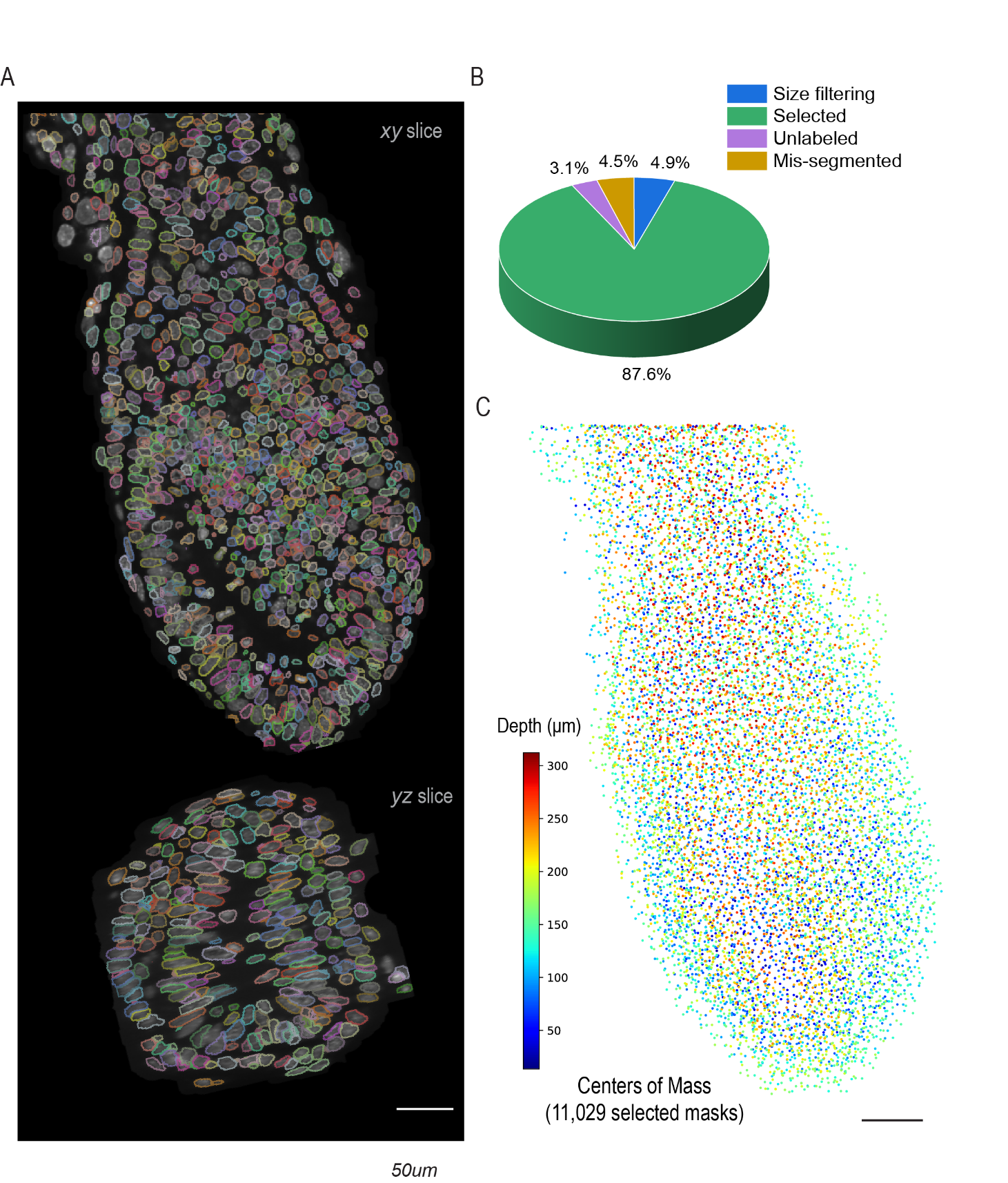
­**

**fig. S20. 3D cell segmentation with a specialist model by Cellpose.**

**(A)** This panel presents both *xy* and yz slice views of the segmented cells within the embryo by Cellpose using a human-in-the-loop custom trained model. The contours delineate DAPI stained nuclei, allowing for detailed examination of cell segmentation result. The images were rendered using ORS dragonfly.

**(B)** A pie chart details the results of the cell segmentation, categorizing the masks into groups: those filtered out by size criteria, selected, unlabeled, and mis-segmented.

**(C)** A scatterplot representing the centers of mass for all 11,029 selected masks, with the *z*-axis depth of each point color-coded according to the accompanying color bar.

**
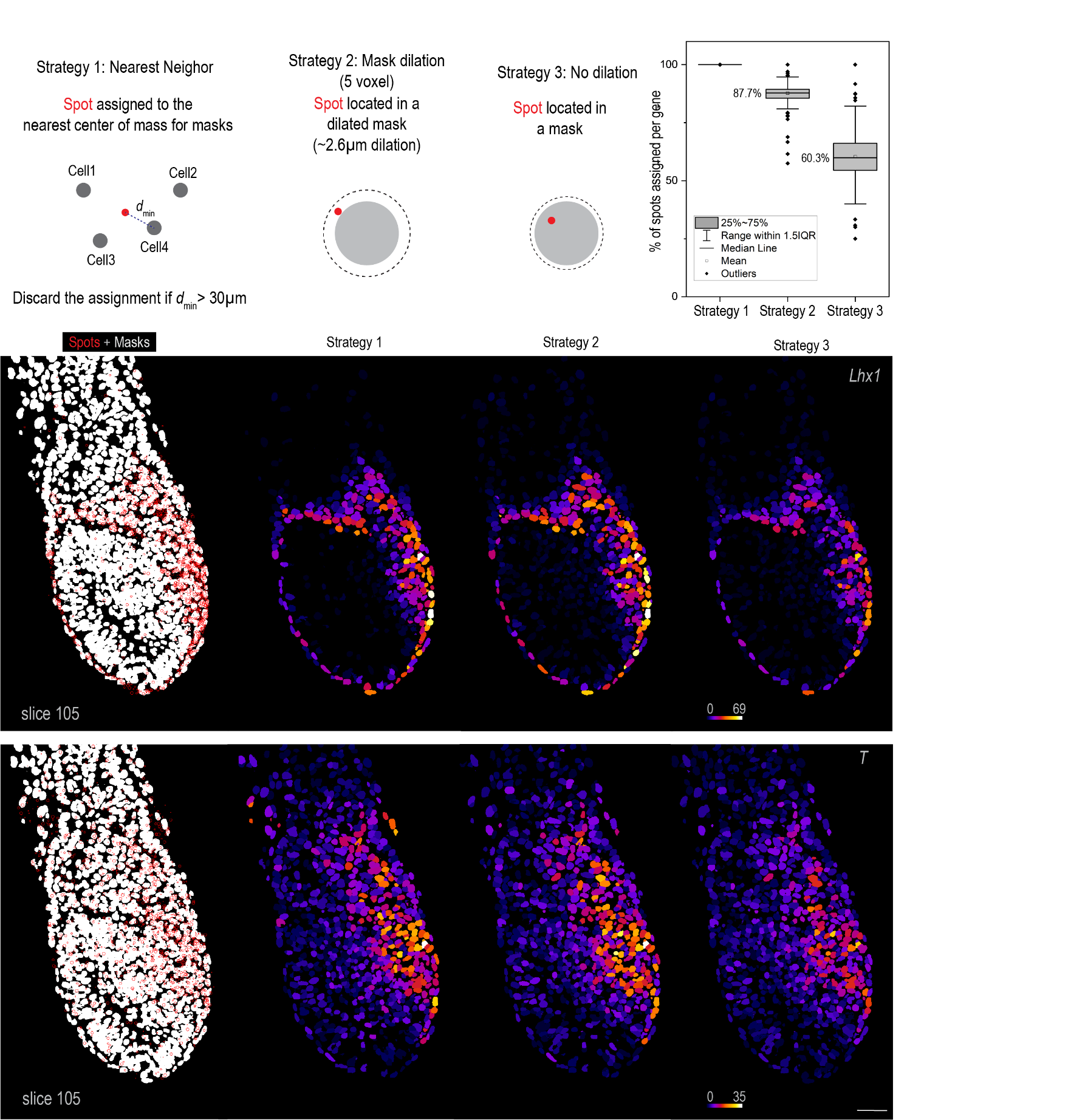
**

**fig. S21. Spot-to-cell assignment and visualization.**

Following single-molecule localization of cycleHCR RNA signals and cell segmentation outlined in fig. S17 and S20, three spot-to-cell assignment strategies were evaluated. The first strategy assigns each RNA spot to the nearest center of mass for masks, discarding assignments if the minimal distance exceeds 30 µm. The second strategy utilizes dilated masks (achieved through 5 voxel iterative non-overlapping dilations in an isotropically resampled volume with 0.52 µm voxel size), while the third assigns spots based on original masks without dilation. It is worth noting that cellpose masks are slightly dilated compared to DNA counterstained nuclei (Fig. 2E). The percentage of spots assigned per gene per strategy is showed in the box plot (n = 254) with mean levels noted in the panel.

Visual representation includes localized RNA spots as red dots against white-colored nucleus masks (Left panel). Spot counts are integrated into cell mask labels within the image stack. Representative *xy* slice views (slice 105) for genes *Lhx1* and *T* under different assignment strategies show single-cell gene expression levels, color-coded for each cell according to a color bar located at the bottom of the image. Scale bar: 50 µm.


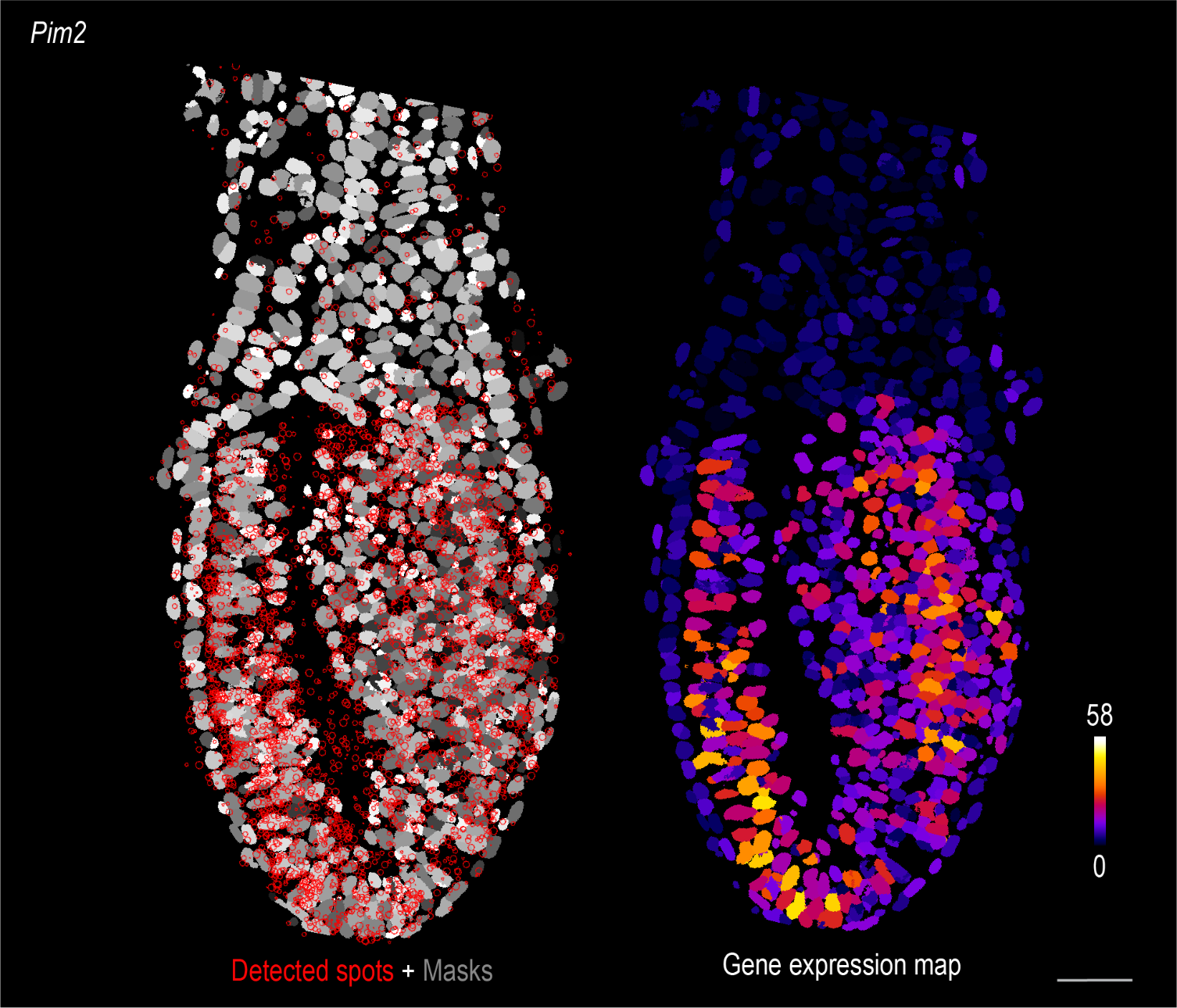
­

**fig. S22. Spot-to-mask overlap and gene expression map.**

Left: A 2D slice of the mouse embryo showing the overlap between detected spots (red circles) and nuclear masks (grayscale). Right: Gene expression map for *Pim2* mRNA, where spots are assigned to individual cells based on Cellpose-generated masks. Cells in the slice are color-coded according to transcript counts, as indicated by the provided color map. Scale bar: 50 µm.


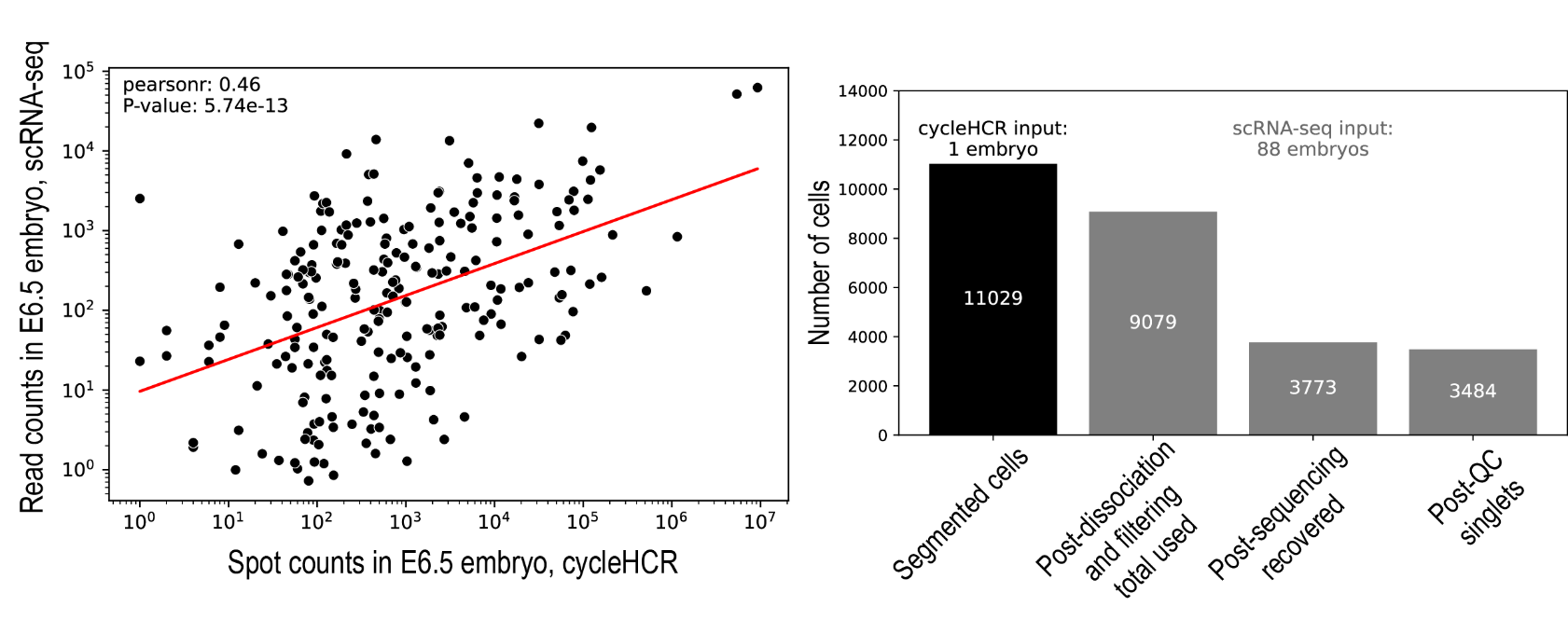


**fig. S23. Cross-method comparison with scRNA-seq.**

To assess the accuracy of gene expression captured by cycleHCR in the mouse embryo sample, we compared cycleHCR counts to aggregated scRNA-seq data from different stages. We found the highest correlation between our data and scRNA-seq from the E6.5 mouse embryo (*23*). For cycleHCR, all RNA spots within the embryo were used, and 6 outlier genes were excluded. Each dot represents a gene, and the Pearson correlation coefficient was calculated using log-transformed data. The bar graph displays the number of cells segmented from a single embryo in cycleHCR. In contrast, the scRNA-seq dataset for this stage was generated by pooling 88 embryos, resulting in ~9,079 cells post-dissociation, with 3,484 cells (~39.5 cells per embryo) passing sequencing quality control (QC).

**
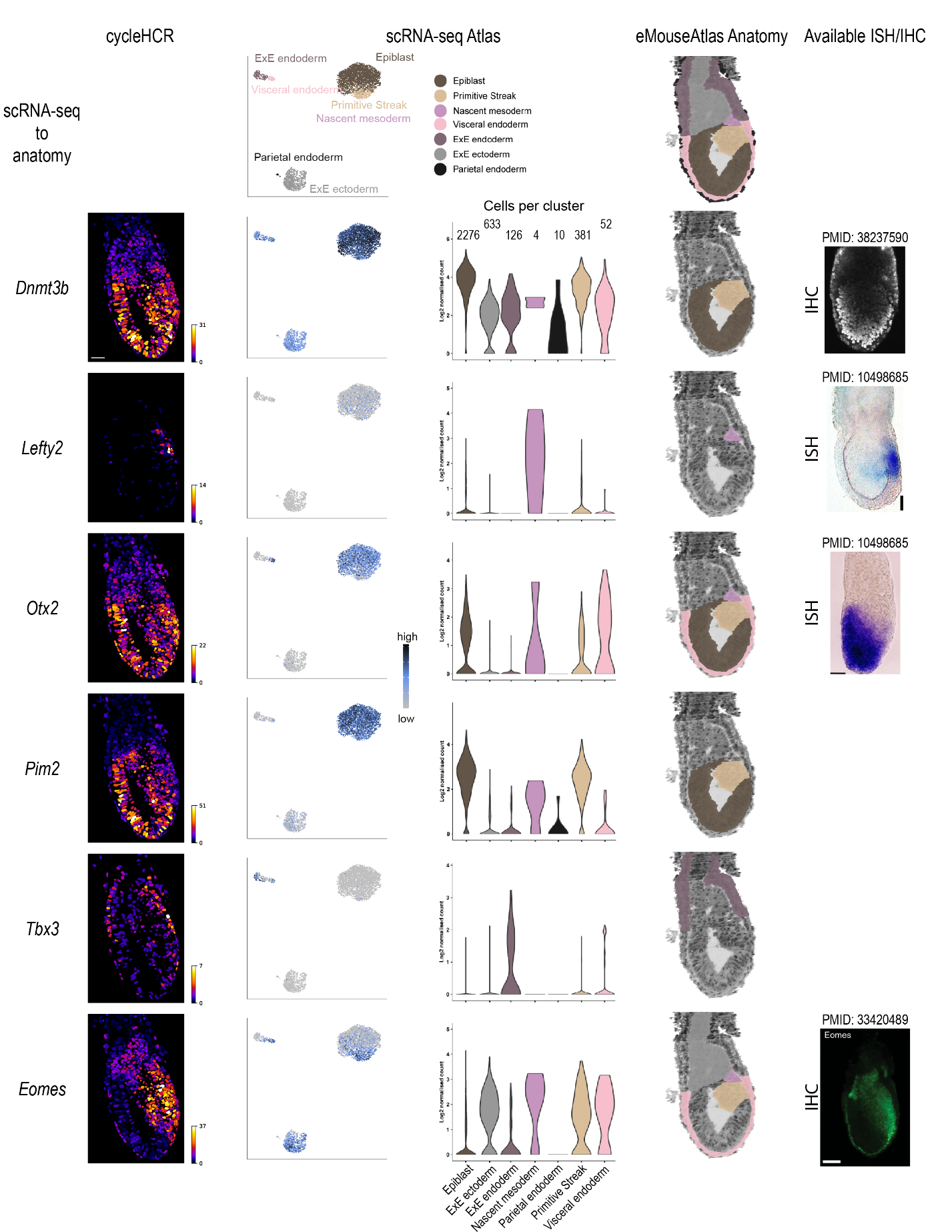
**

**
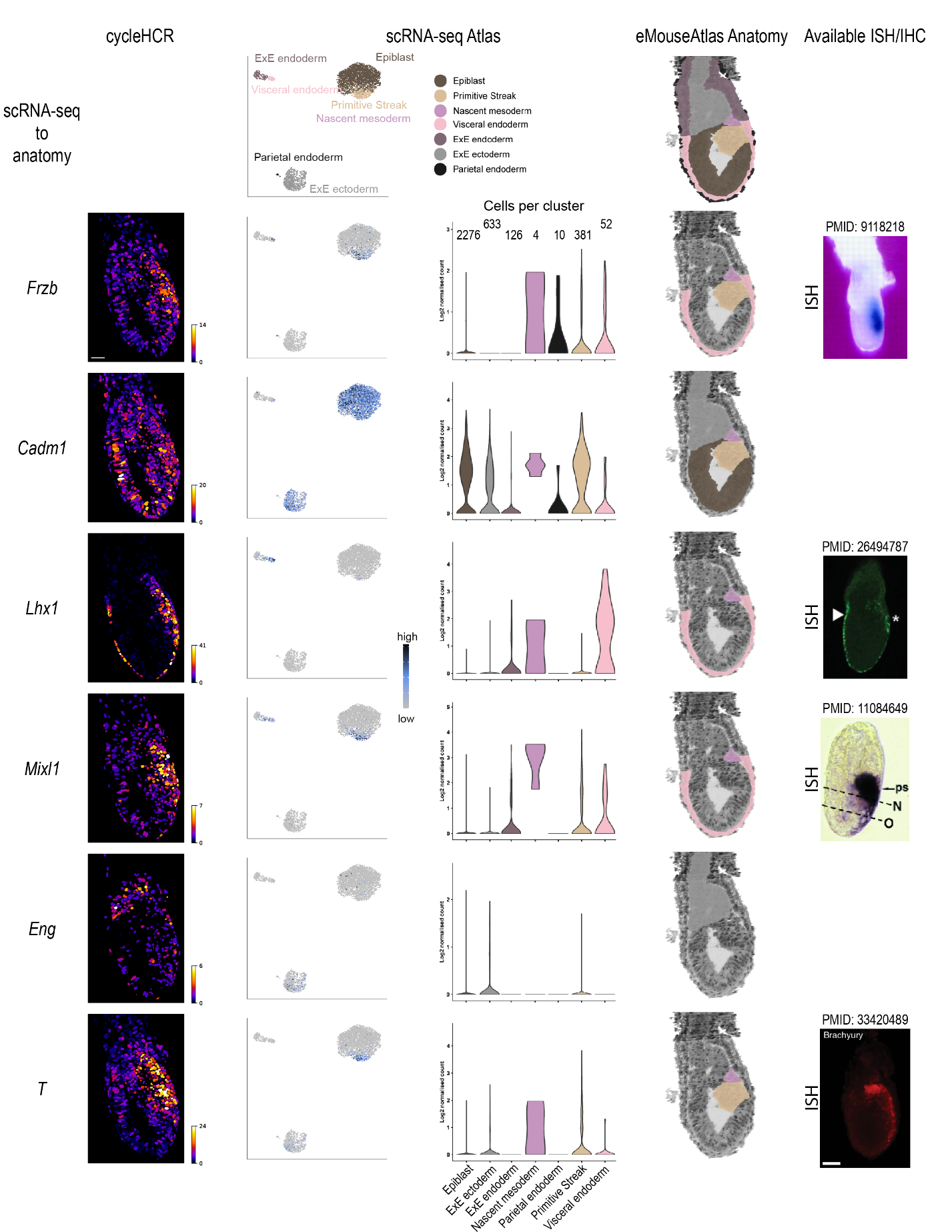
**

**
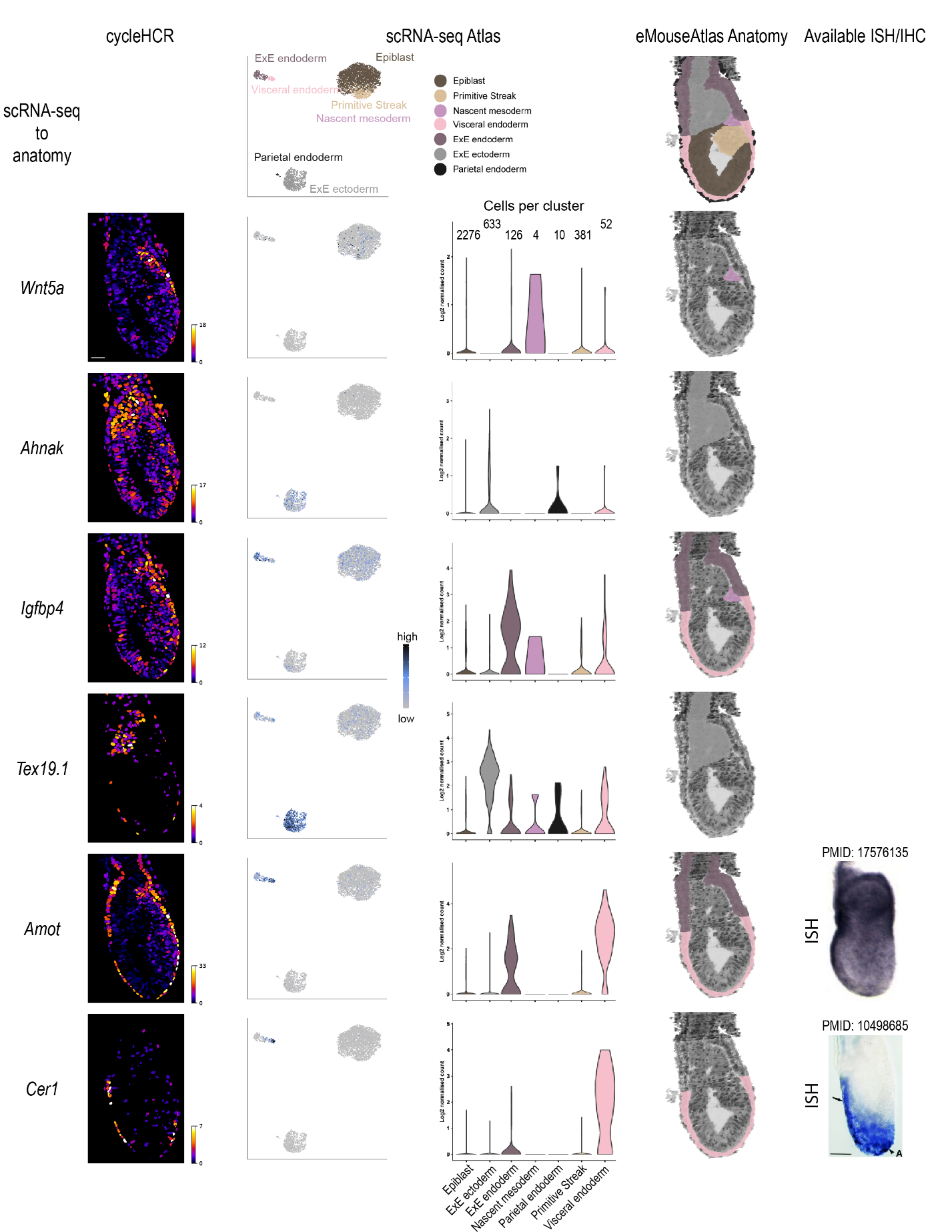
**

**
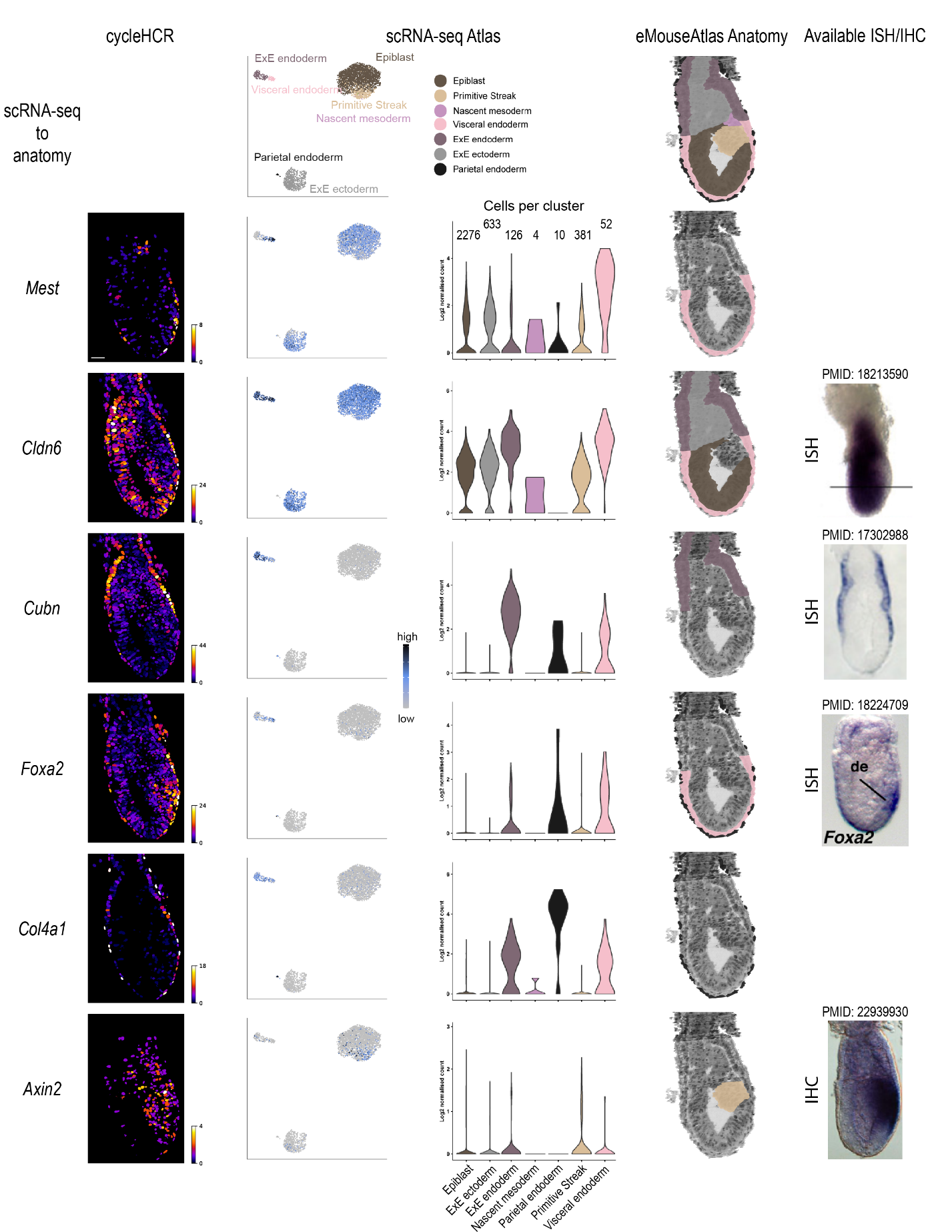
**

**fig. S24. Cross-method comparison with spatially inferred scRNA-seq and ISH/IHC data.**

Spatial gene expression data for the mouse embryo during early development is limited. To address this, we first translated gene enrichment from scRNA-seq UMAP clusters (*23*) into anatomical information using the eMouseAtlas (*36*) and manually collected ISH/IHC data from individual publications (*25-35*). A representative 2D slice of cycleHCR gene expression is shown on the left. The middle-left panel displays gene imputation in the UMAP, along with gene expression enrichment per gene per cluster. The middle-right panel shows the corresponding regions from the eMouseAtlas where the gene is expressed, while ISH and IHC data are presented on the far right. Scale bar: 50 µm

**
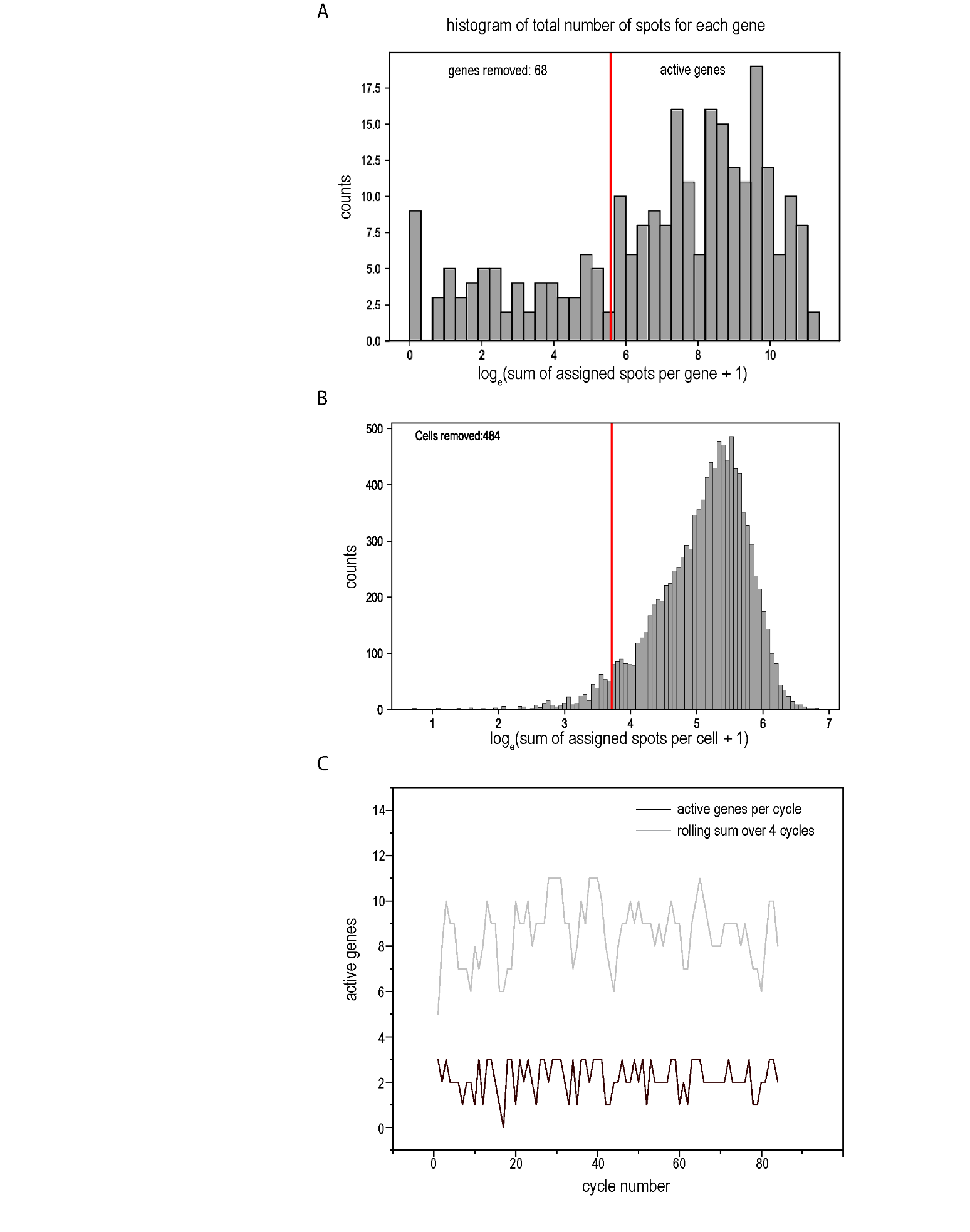
**

**fig. S25. Gene expression thresholding, cell filtering and sample integrity.**

**(A)** A histogram showing the distribution of total spot counts for each gene across all cells on a logarithmic scale to accommodate the broad range of expression levels. Genes with fewer than 410 total spots are removed based on Otsu’s method (*77*), retaining 186 genes for subsequent analysis.

**(B)** A histogram presenting the total number of spots assigned per cell, also on a logarithmic scale. Cells with spot counts below 40, approximately 484 cells (5% of the total), were excluded from further analysis.

**(C)** To evaluate the detection of actively transcribed genes over increasing cycle numbers, the number of active genes detected (as shown in (A)) is plotted as a function of the cycle number. We observed stable detection of active genes across 85 cycles, indicating that RNA and primary probes were well-preserved during cycleHCR imaging.


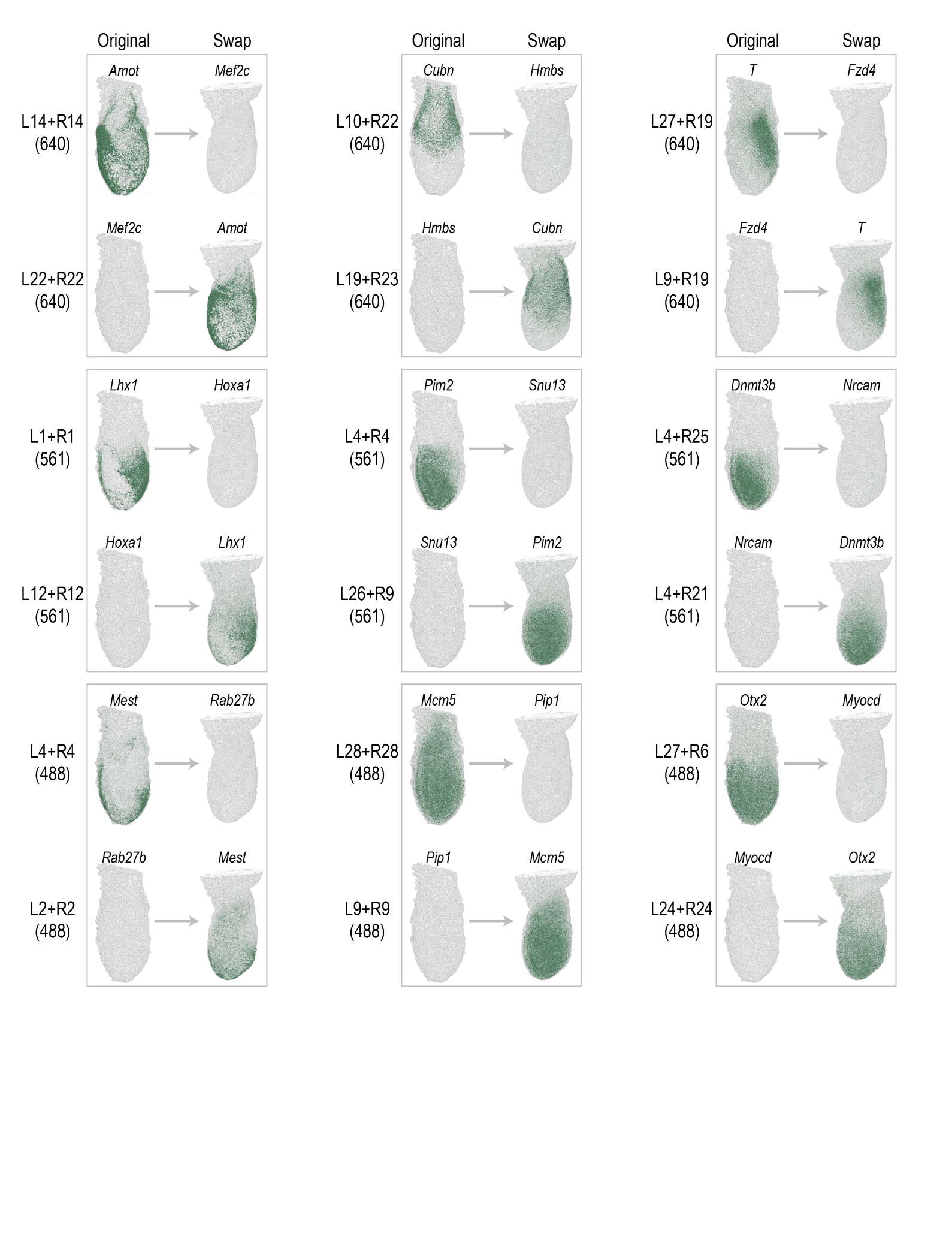


**fig. S26. Barcode swapping between active and silent genes.**

To determine if the low number of spots detected in certain genes is due to barcode issues, we performed barcode swaps between 3 silent and 3 active genes per color channel, covering 18 genes across 3 color channels. Barcode swaps resulted in swapped gene expression patterns, indicating that the low spot counts are related to gene expression levels rather than the barcode identity. The raw RNA localization patterns (dark green) are overlaid onto the embryo mask. Rendered using the napari Python package. Scale bar: 50 µm.


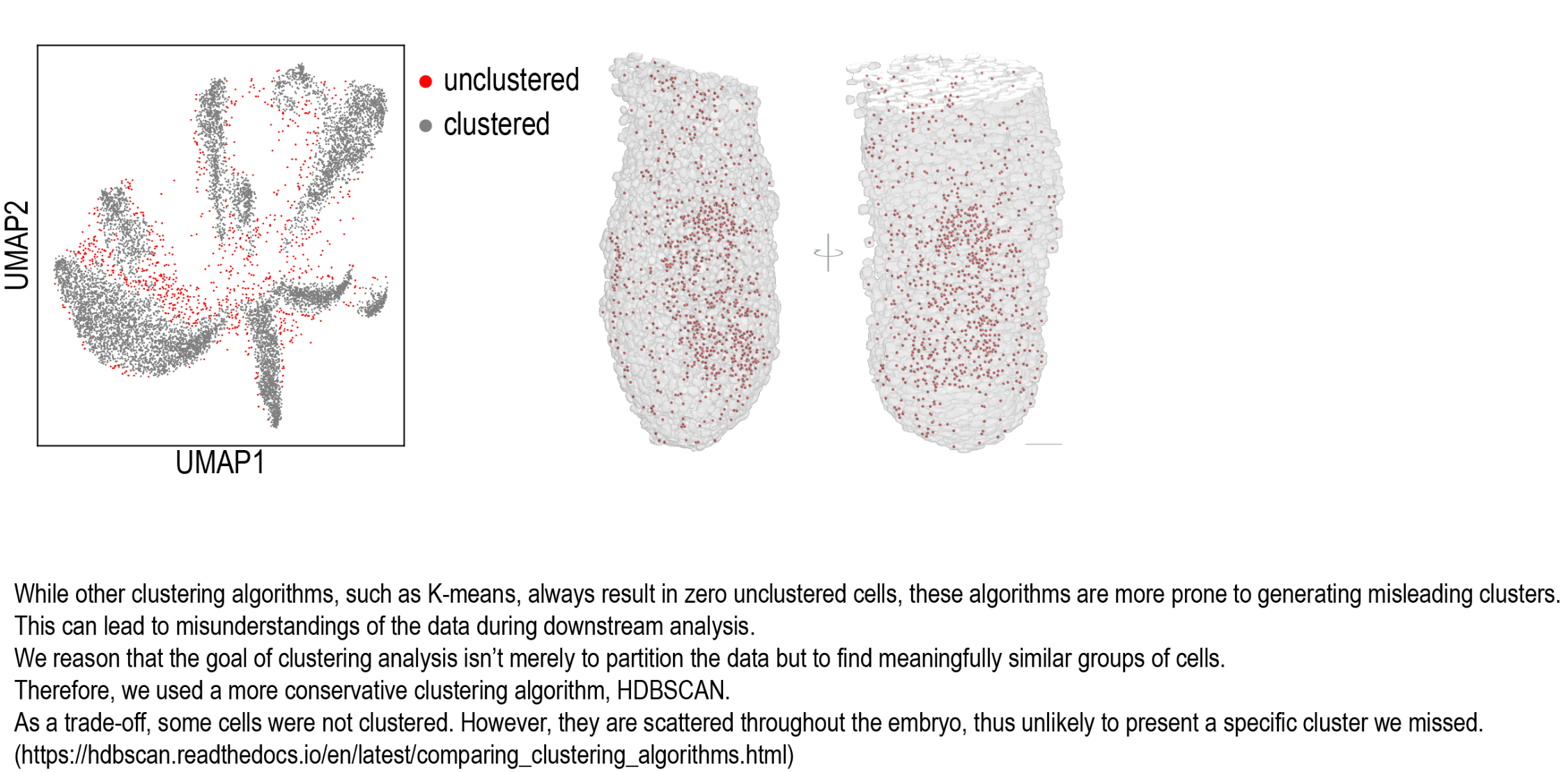


**fig. S27. UMAP and 3D physical localization of unclustered cells.**

While algorithms like K-means often produce zero unclustered cells, they are more susceptible to creating misleading clusters, which can misrepresent the data during downstream analysis. Since the primary goal of clustering is to identify meaningfully similar groups of cells, we applied a more conservative clustering algorithm, HDBSCAN (*80*). As a result, some cells remained unclustered. These unclustered cells are scattered throughout both the UMAP and the physical space within the embryo, suggesting that they do not represent a missing cluster. Scale bar: 50 µm.


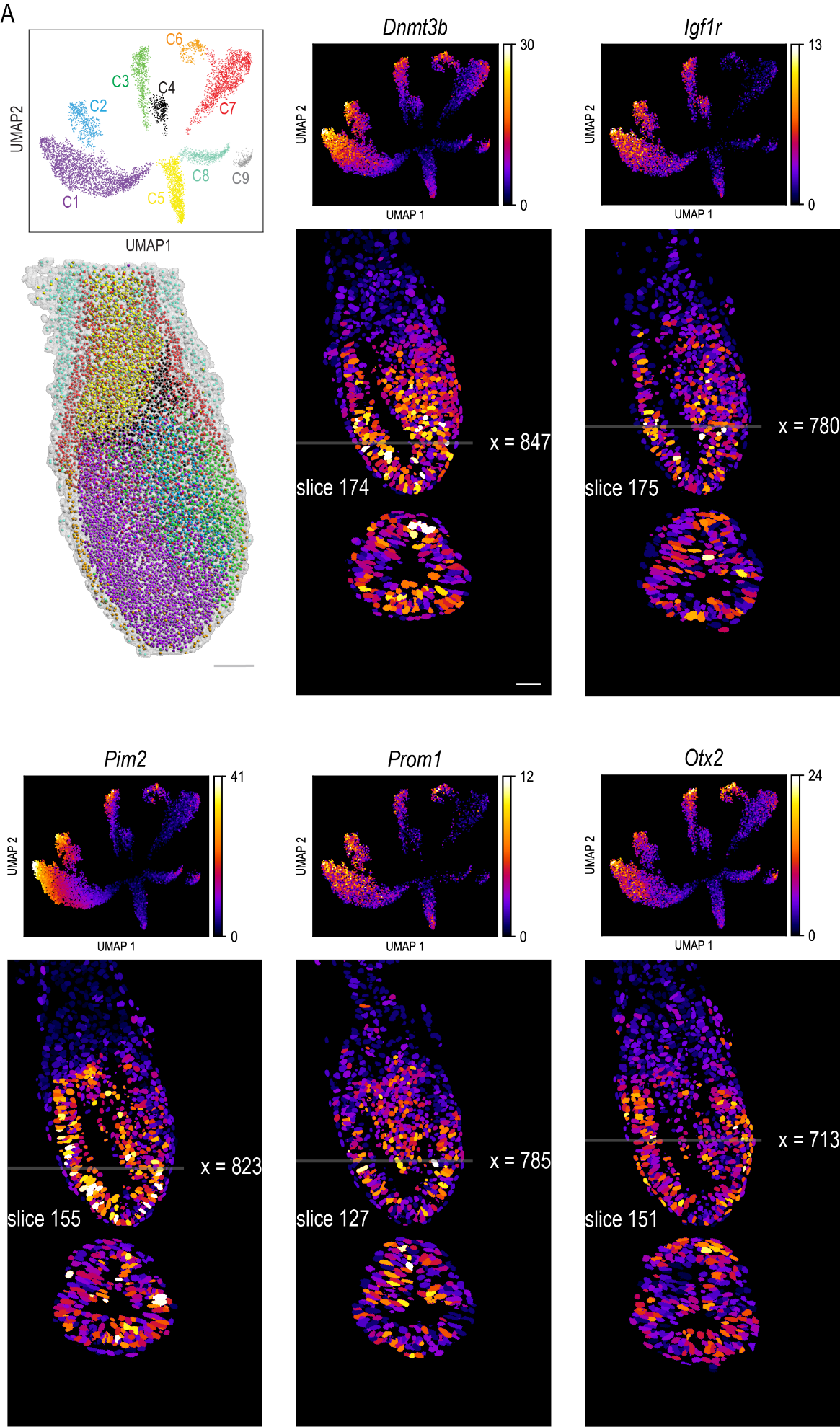


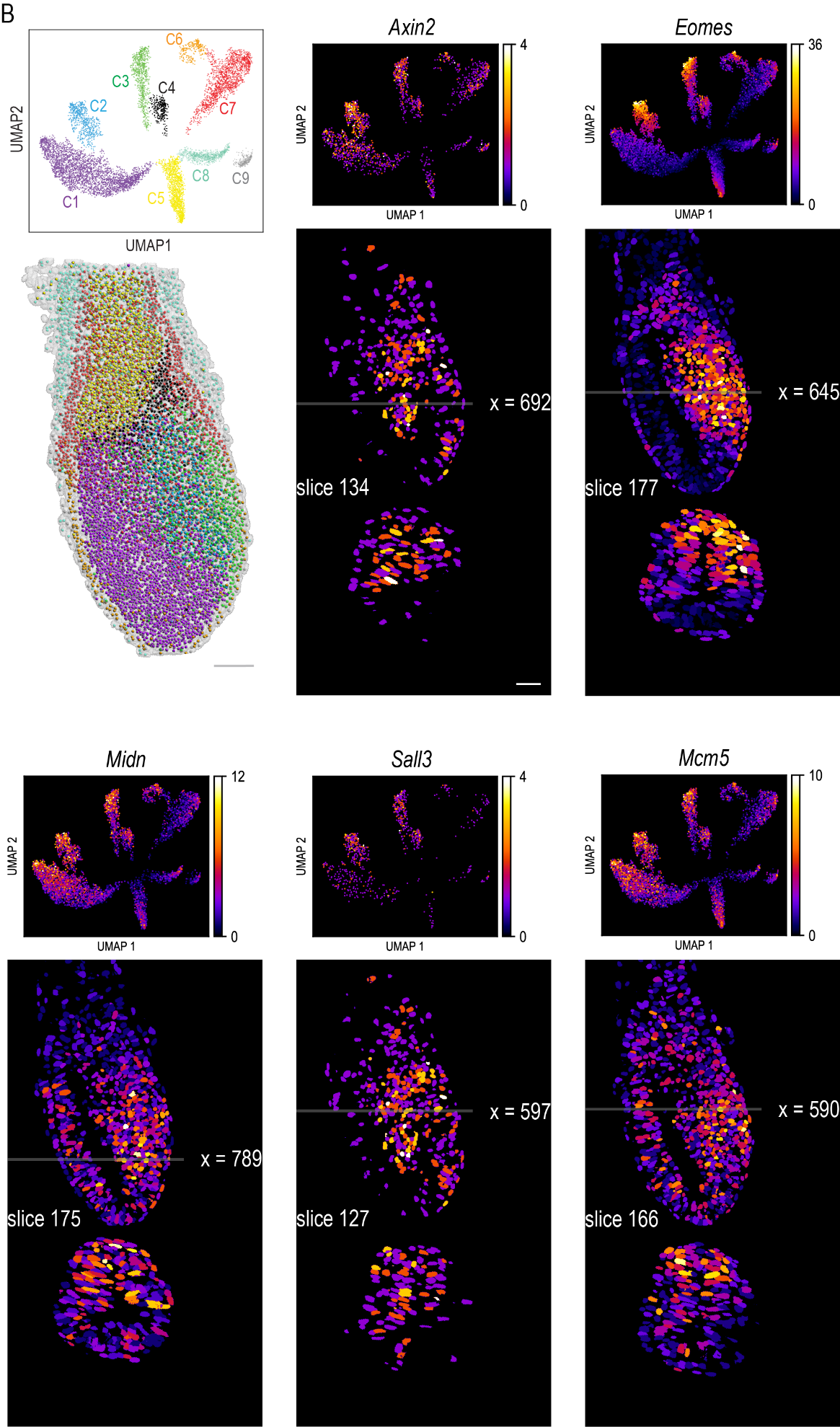


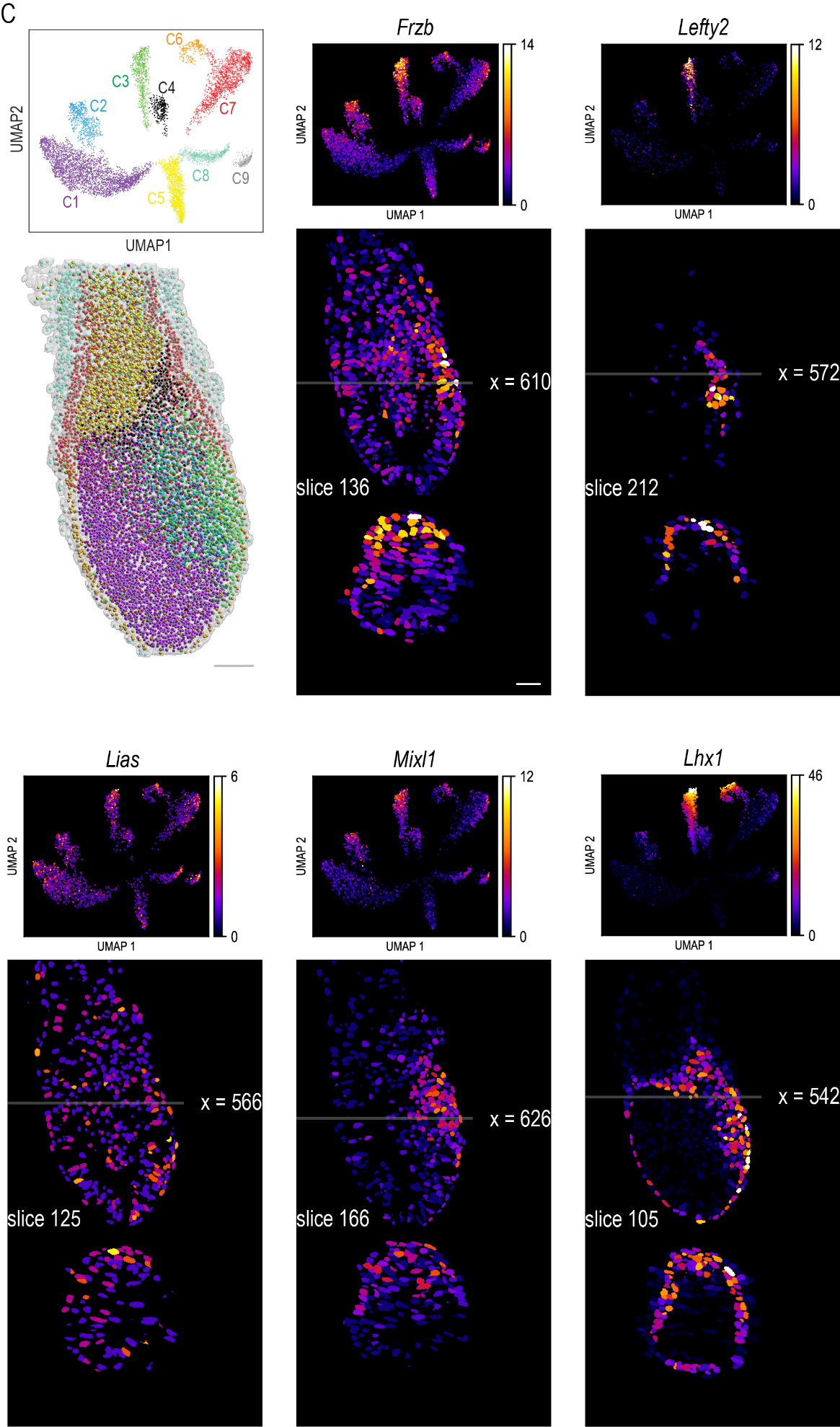


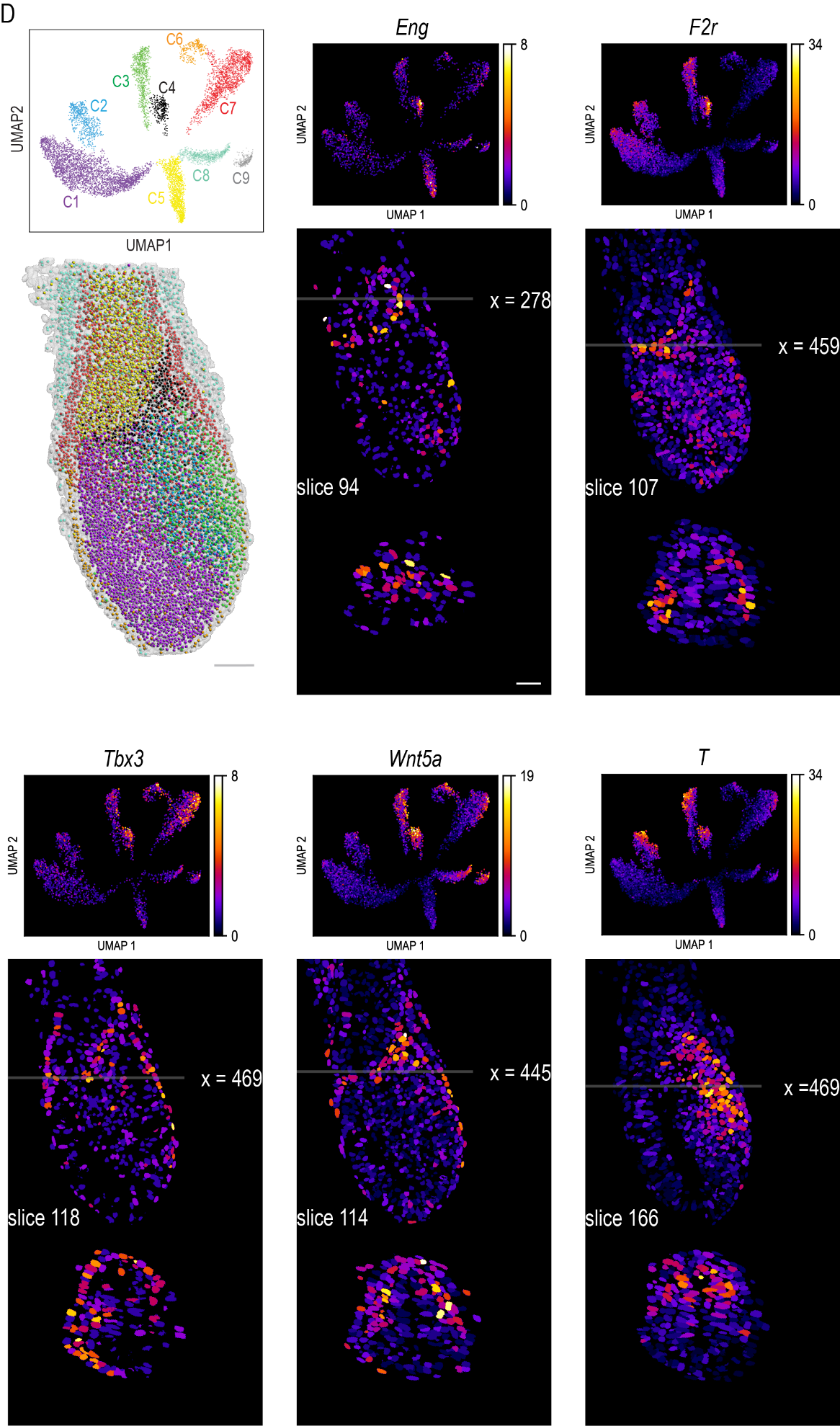


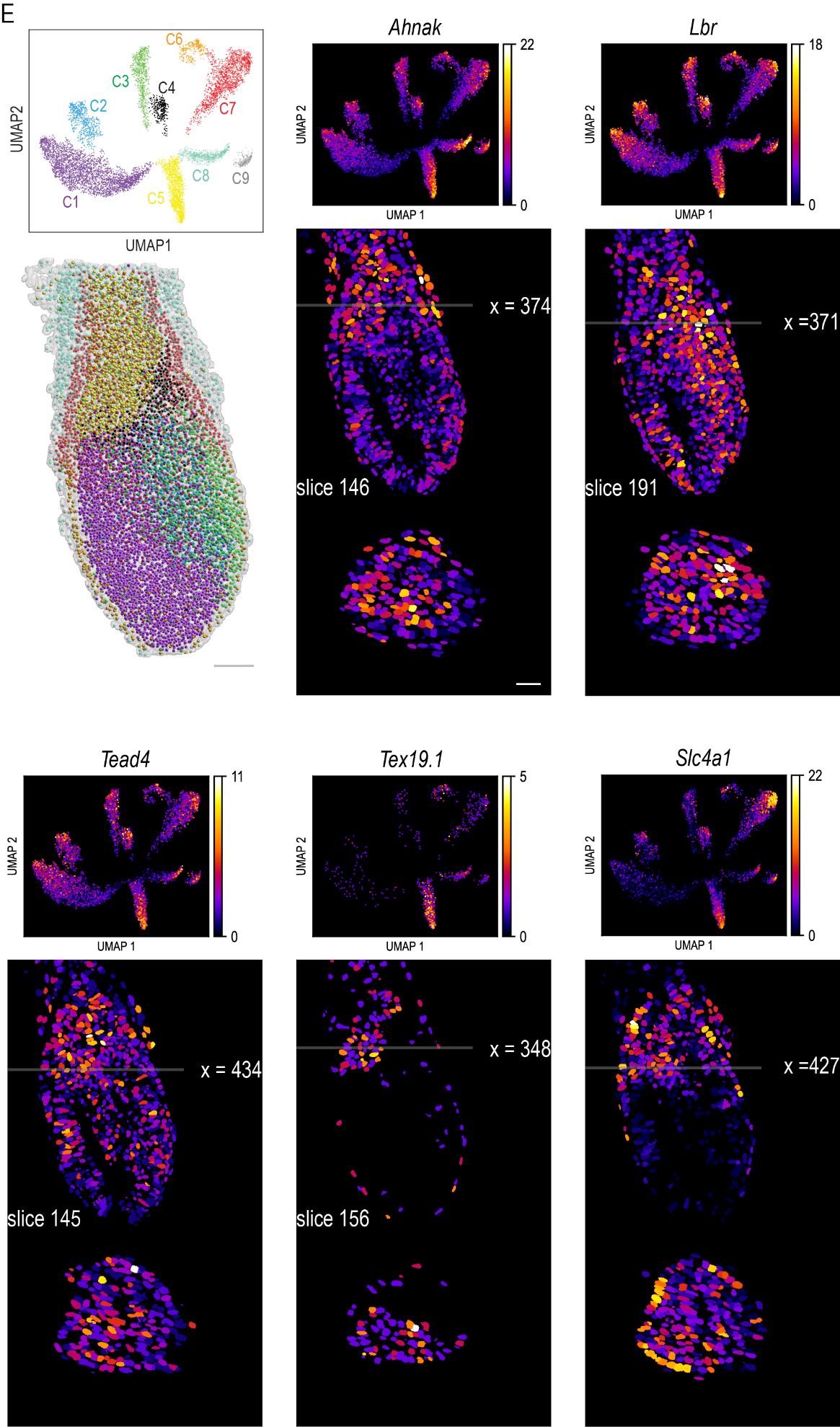


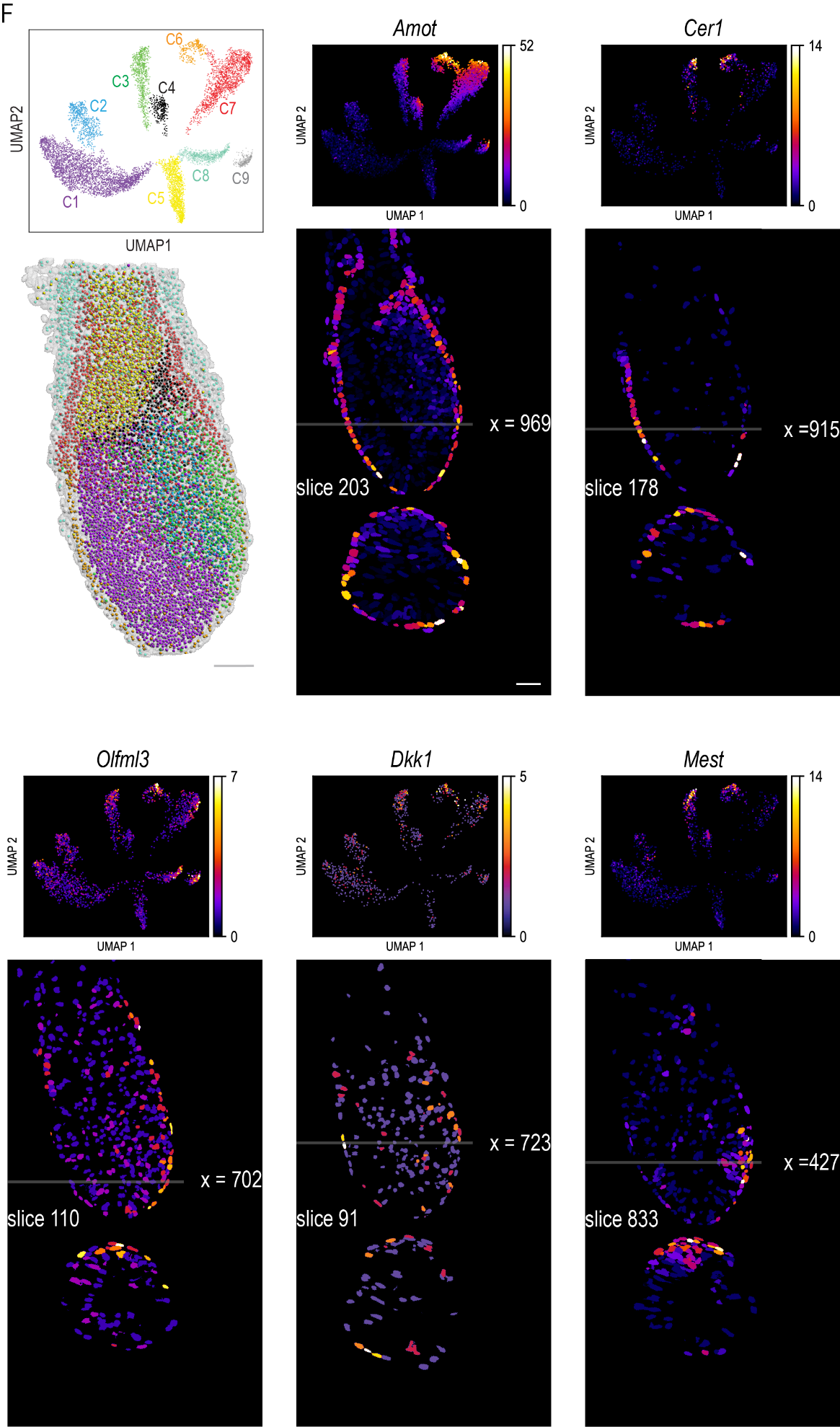


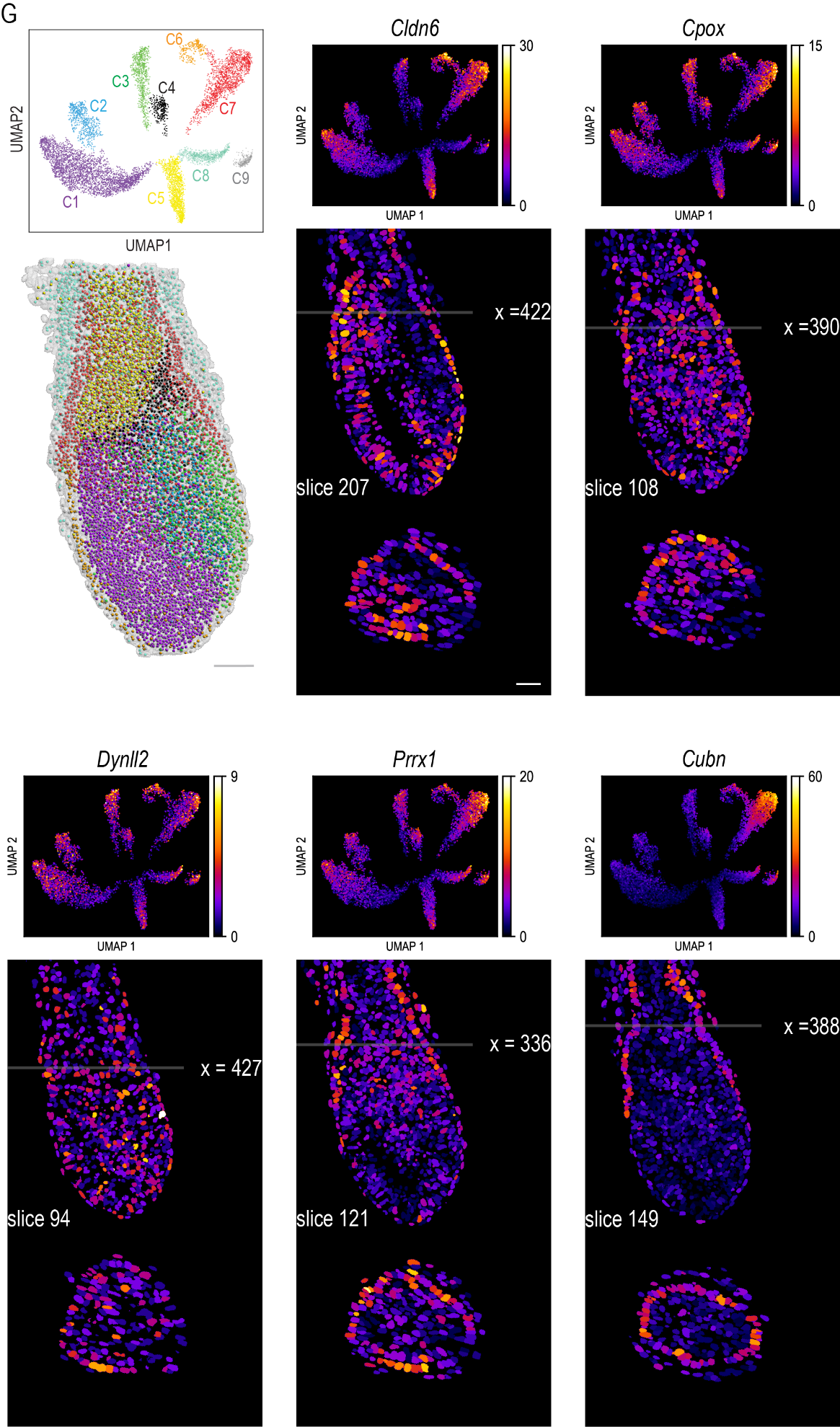


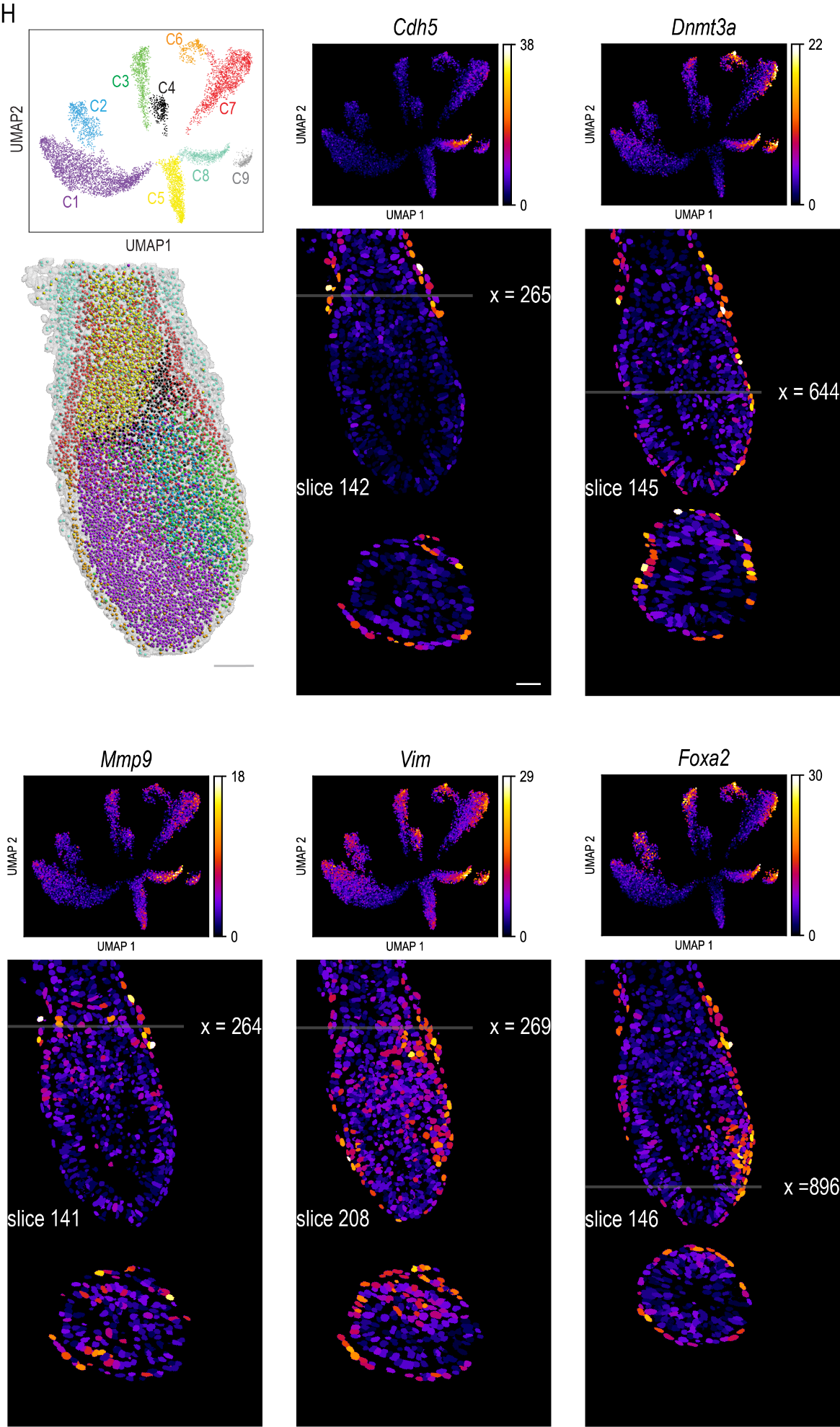


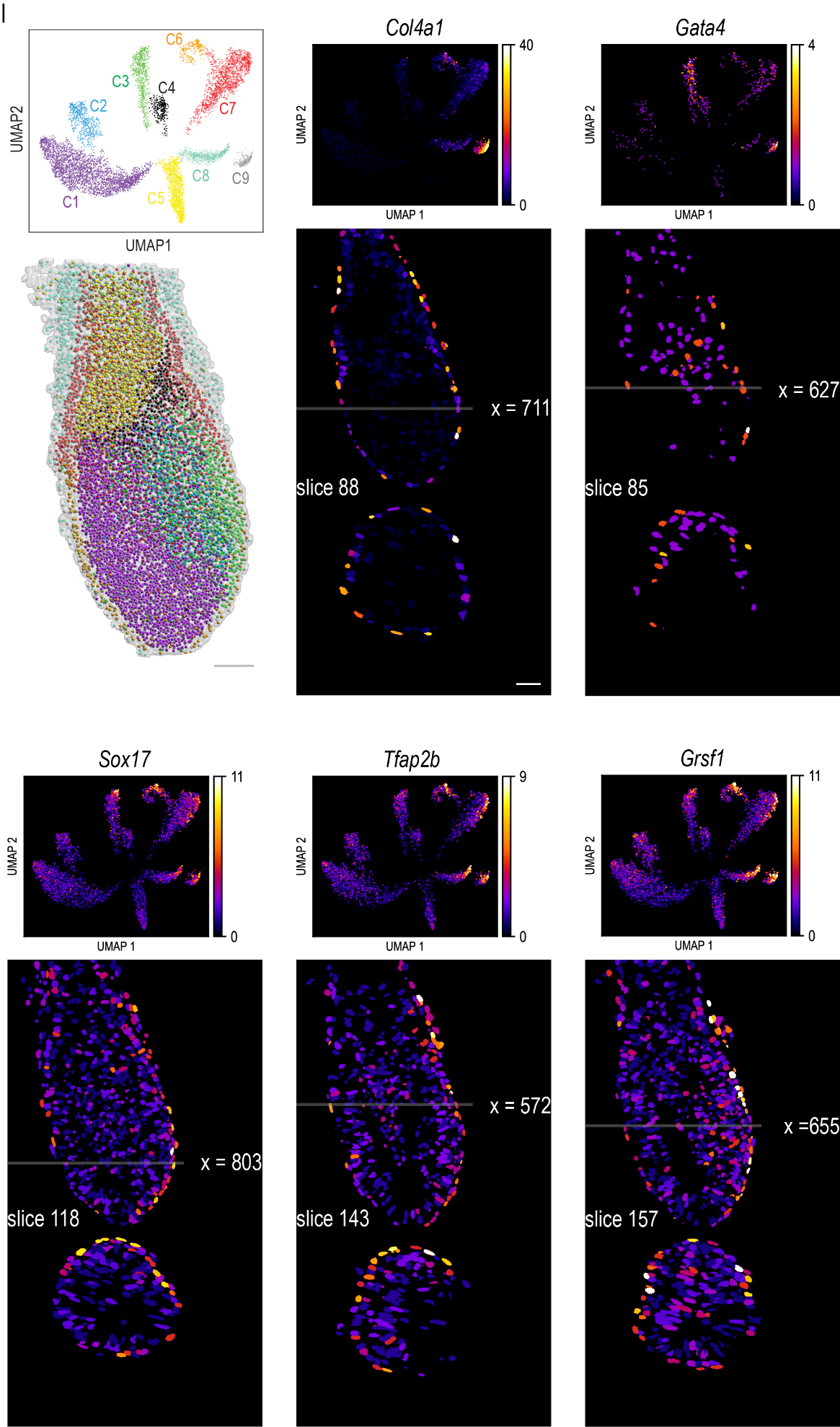


**fig. S28. UMAP imputation and 3D spatial gene expression.**

**(A-I)** Gene expression levels for five selected genes for each of the nine clusters are color-coded in the upper panel, with panels A-I representing clusters 1-9, respectively, providing a visual representation of gene expression variations among clusters. The lower panel offers selected *xy* and *yz* views of the embryo, illustrating the spatial distribution of gene expression throughout the embryo. Unified color maps adjacent to UMAP panels aid in interpreting the gene expression levels. Scale bar: 50 µm.

**­**

**
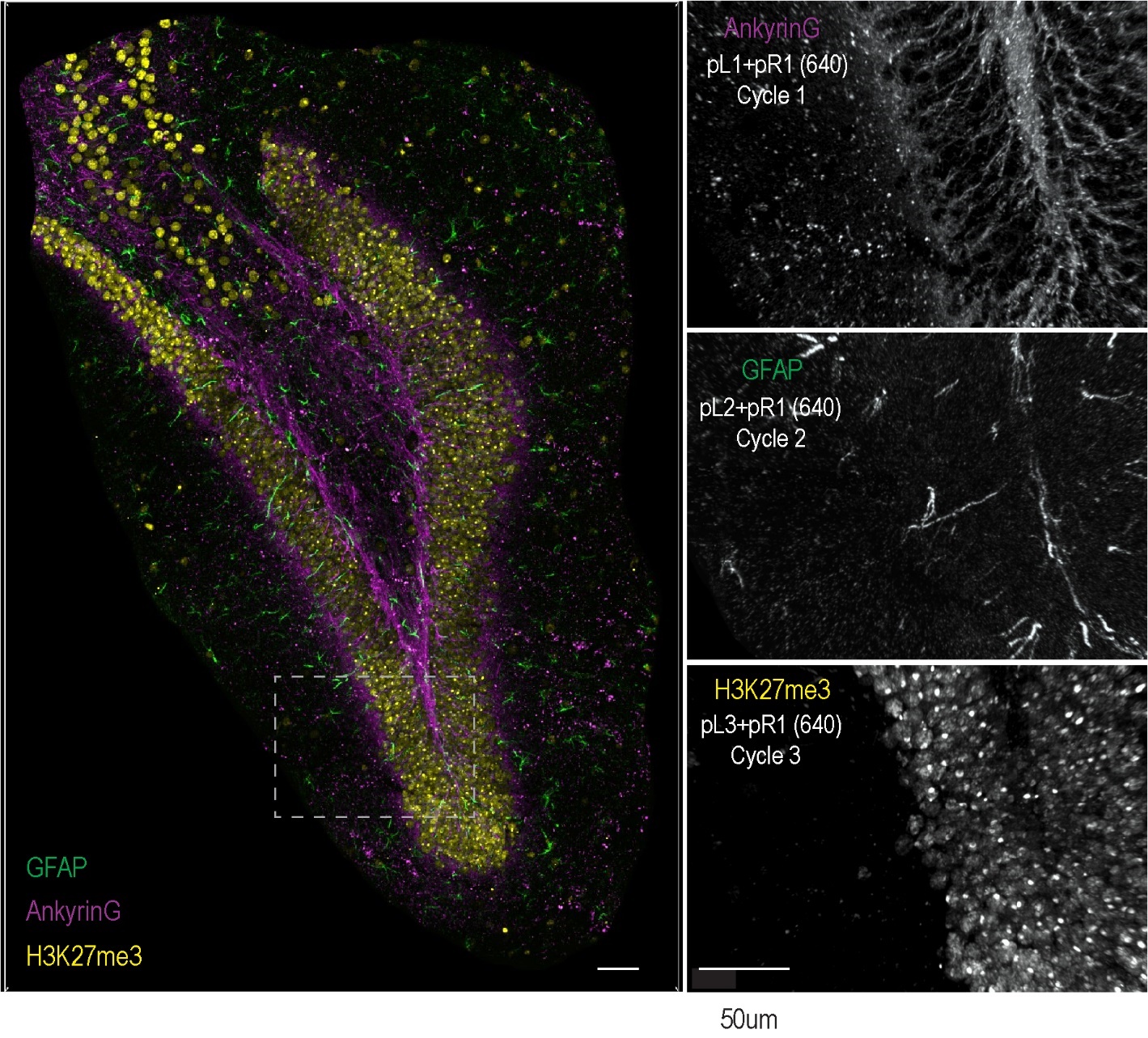
**

**­**

**fig. S29. Demonstrating cycleHCR protein labeling specificity in tissue.**

This figure shows the capacity of cycleHCR to achieve protein labeling specificity within a complex tissue matrix, specifically in the dentate gyrus of a 40 µm thick hippocampal slice. By maintaining a constant right readout probe (pR1 (640)) and sequentially changing the right readout probes, cycleHCR shifted its target from the axonal initial segment marker ankyrin G (pL1 + pR1 at 640 nm, Cycle 1) to the glial cell marker GFAP (pL2 + pR1 at 640 nm, Cycle 2), and finally to the heterochromatin marker H3K27me3 (pL3 + pR1) at 640 nm, Cycle 3). The composite image is on the left panel and zoom-in cross-cycle views of the boxed region are displayed on the right. Scale bar: 50 µm.

**
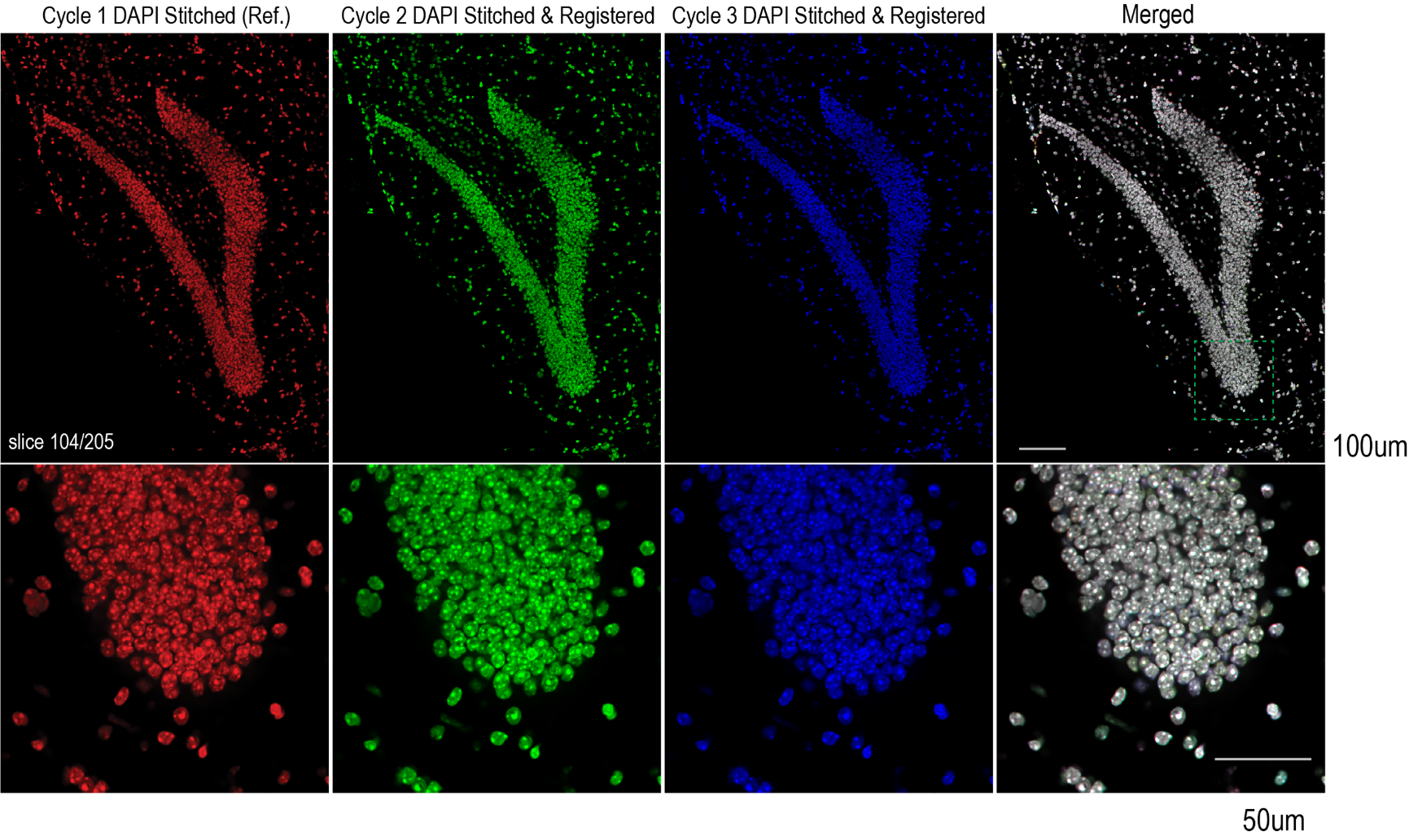
**

**fig. S30. Cross-cycle registration validation for protein cycleHCR in mouse hippocampal slice.**

This figure illustrates the cross-cycle registration for protein cycleHCR imaging using a mouse hippocampal slice showed in fig. S29. The alignment of the reference DAPI image (depicted in red) with the DAPI images from subsequent cycles—Cycle 2 (shown in green) and Cycle 3 (in blue). A detailed examination within the boxed area in the upper panel underscores the precise alignment of DAPI signals across the three consecutive cycleHCR imaging rounds, confirming the reliability of the imaging and registration process over multiple cycles. Scale bar: 100µm (the upper panel); 50 µm (the lower panel).

**
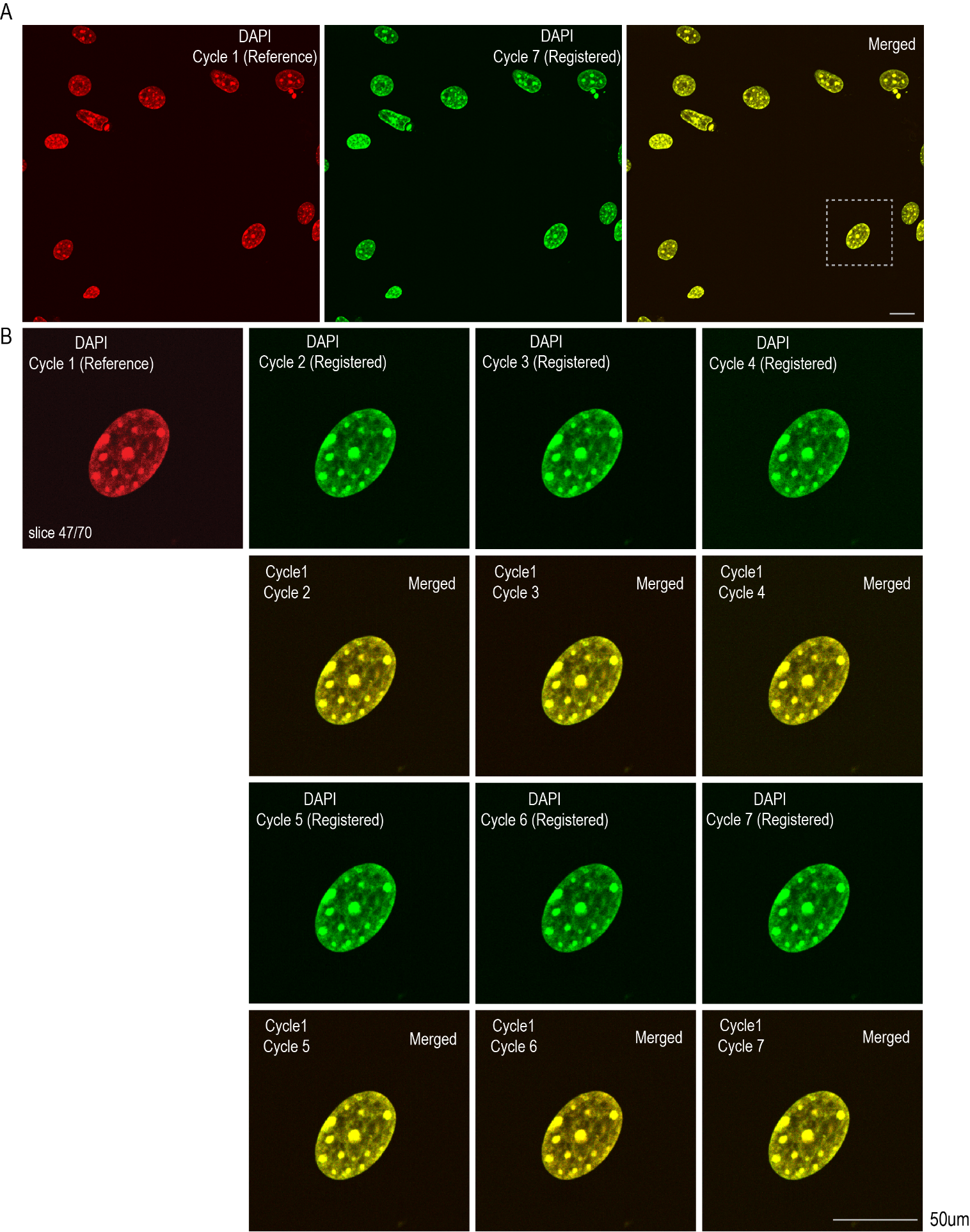
**

**fig. S31. Verifying cross-round registration accuracy in protein cycleHCR imaging of expanded mouse embryonic fibroblasts.**

**(A)** The precision of cross-cycle registration in protein cycleHCR imaging applied to ~4X expanded mouse embryonic fibroblasts associated with Fig. 5. The alignment of the reference DAPI image (depicted in red Cycle 1) with the DAPI images from last cycle — Cycle 7 (shown in green).

**(B)** A detailed examination of the cell within the boxed area in (A): The alignment of the reference DAPI image from Cycle 1 (rendered in red) with DAPI images from subsequent cycles—Cycles 2 through 7 (each displayed in green). The composite image by fusing the reference image with the registered image are showed below, offering a visual assessment of the accuracy of cross-round registration.

Scale bars: 50 µm.

**
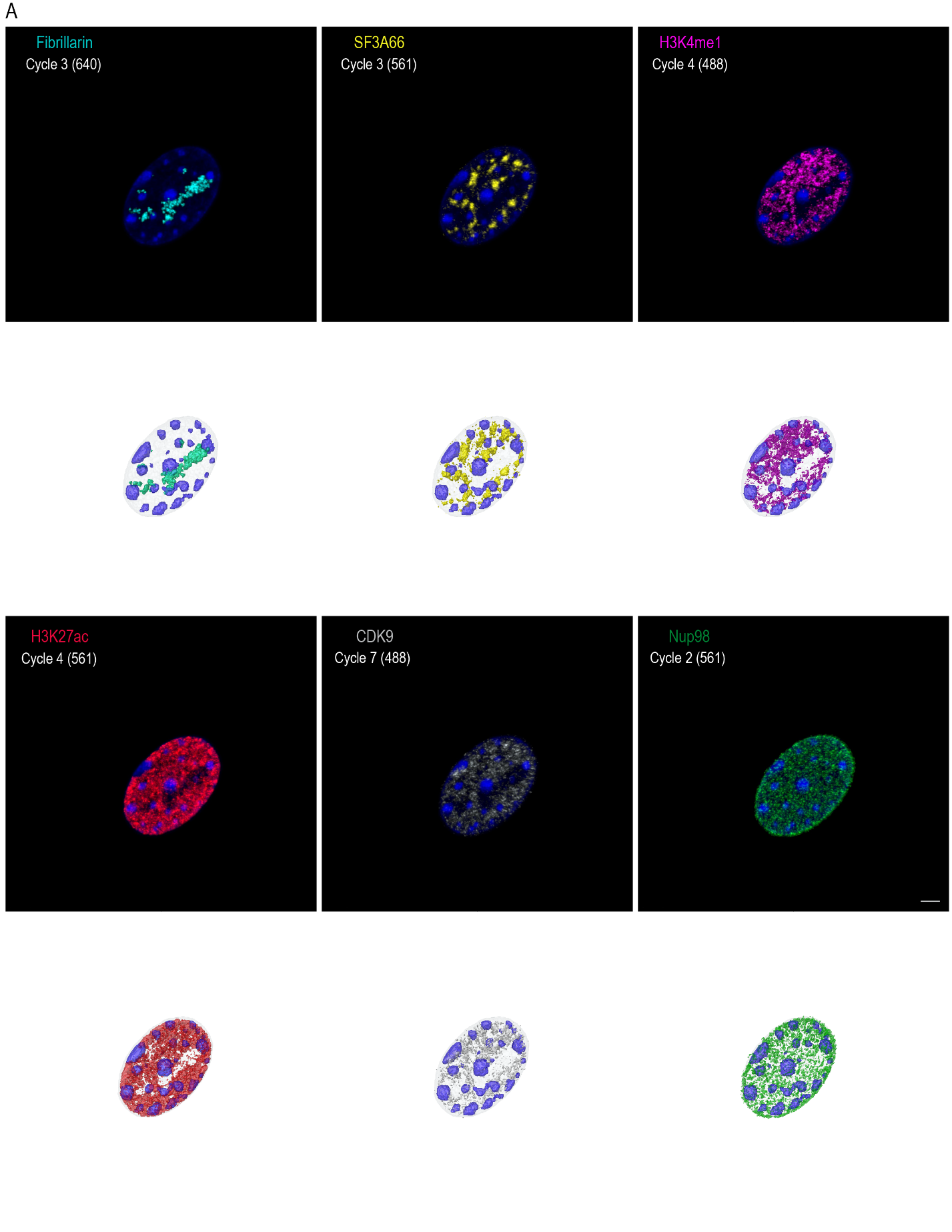
**

**
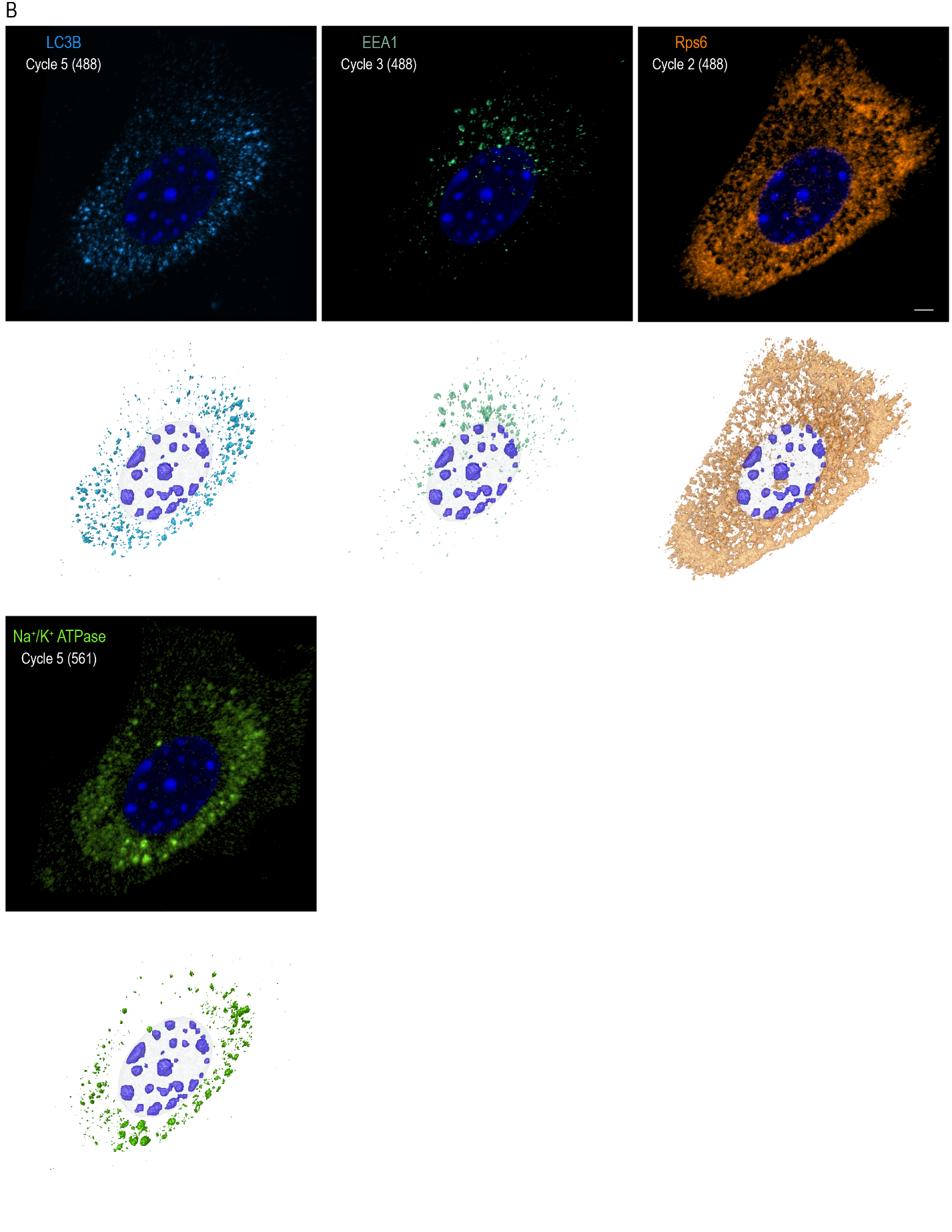
**

**fig. S32. 3D segmented models of subcellular structures.**

Protein cycleHCR images of specific protein targets within a mouse embryonic fibroblast after ~4X expansion are presented by Imaris Maximum Intensity Projection (MIP) volume rendering on the top. Accompanying each fluorescent image, 3D segmentation masks are displayed in mesh format, rendered by ORS Dragonfly.

**(A)** The proteins visualized include Fibrillarin (marking nucleolar fibrillar regions in the nucleolus), SF3A66 (highlighting nuclear speckles), H3K4me1 (associated with enhancers), H3K27ac (indicating active chromatin regions), CDK9 (involved in Pol II elongation), and Nup98 (marking nuclear pores).

**(B) The proteins visualized include** LC3B (autophagosomes), EEA1 (early endosomes), Rps6 (ribosomal subunit), and Na^+^/K^+^ ATPase.

Scale bars: 10 µm.

**
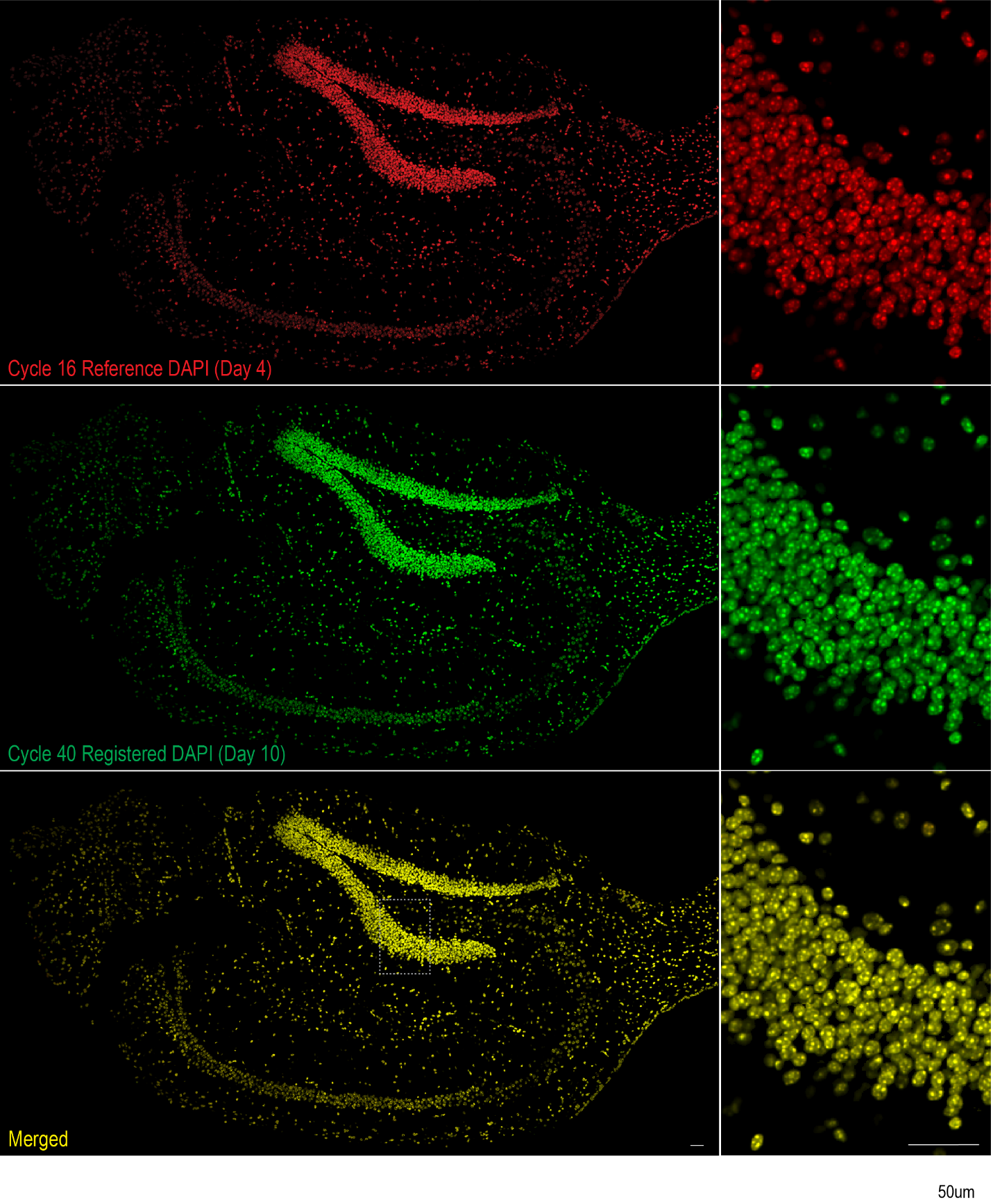
**

**fig. S33. Verifying cross-round registration accuracy in joint cycleHCR protein and RNA imaging of hippocampal slice.**

This figure demonstrates the precision of cross-cycle registration in joint cycleHCR protein and RNA imaging of the hippocampal slice associated with Fig. 6. The alignment of the reference DAPI image (depicted in red for Cycle 15) with the DAPI images from the last RNA cycle (Cycle 40, shown in green) is illustrated. The composite image, created by fusing the reference image with the registered image, is shown below, providing a visual assessment of the accuracy of cross-round registration. Zoomed-in views of the boxed region are displayed on the right.

Scale bars: 50 µm.


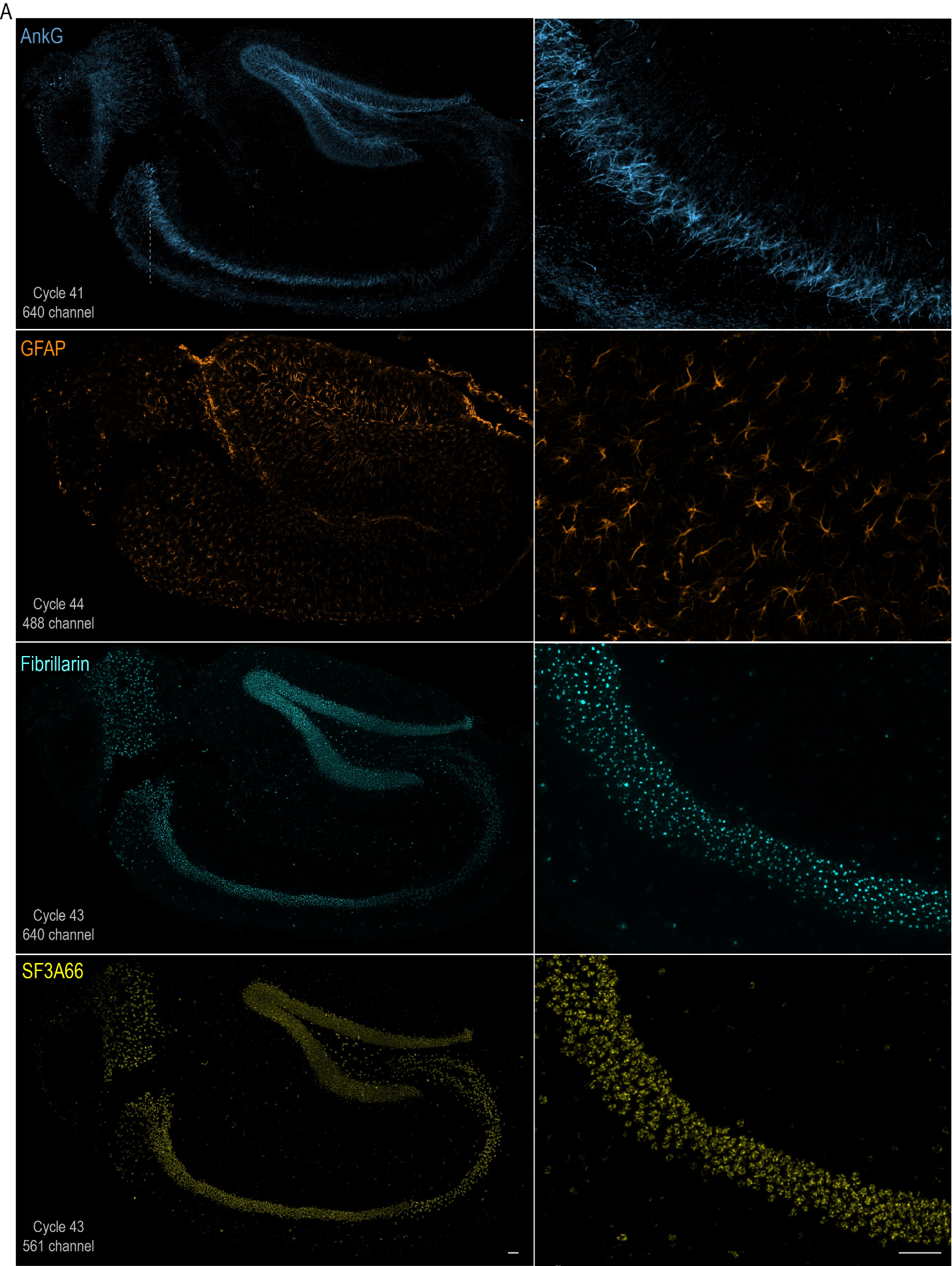


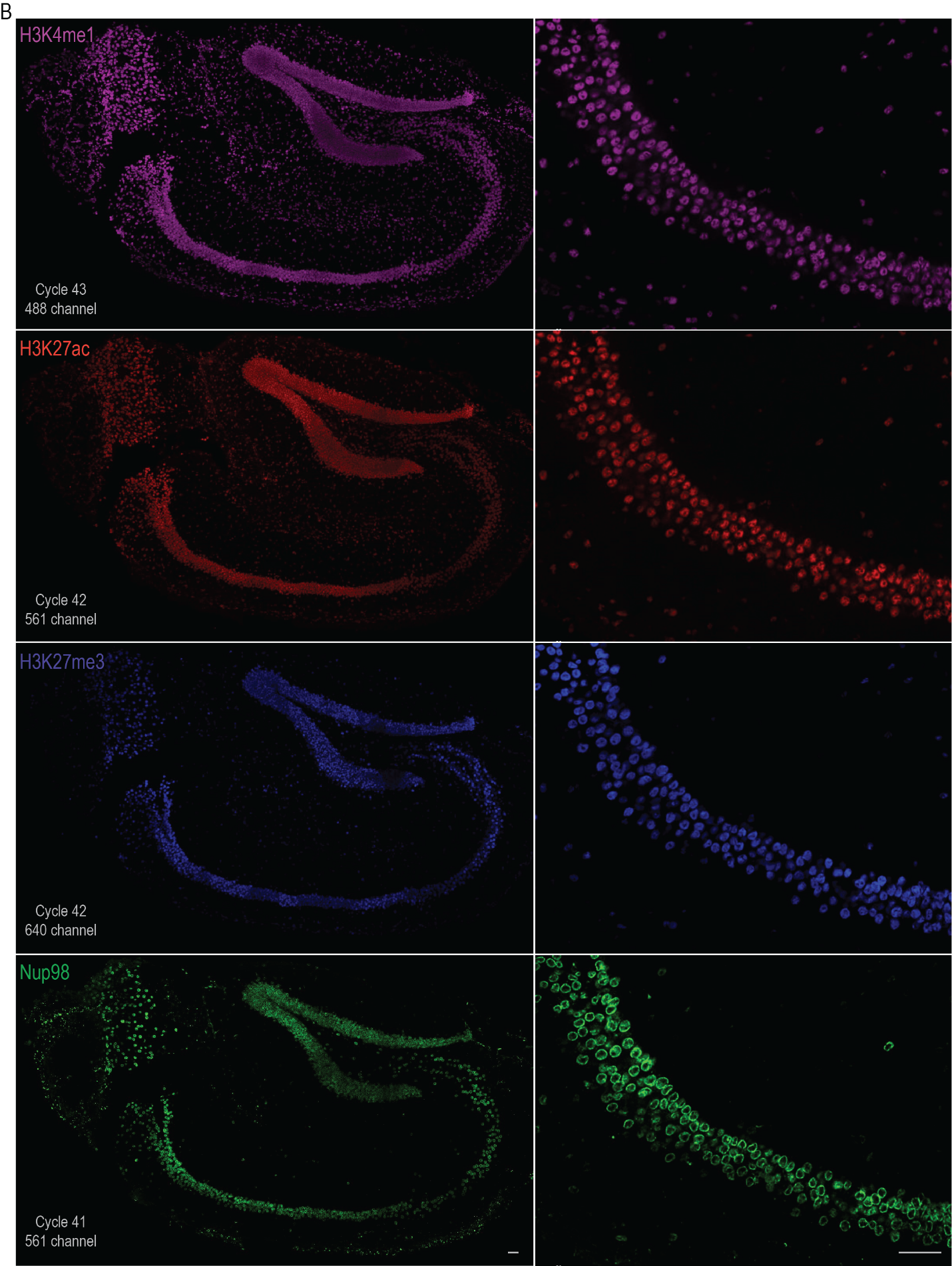


**fig. S34. Protein cycleHCR in hippocampal slice (8 antibodies).**

**(A)** This panel presents stitched and registered images from protein cycleHCR targeting the axon initial segment marker AnkG, astrocyte marker GFAP, nucleolar fibrillar center marker Fibrillarin, and nuclear speckle marker SF3A66 in the hippocampal slice. Zoomed-in images of the boxed region are provided on the right panel.

**(B)** This panel displays stitched and registered images from protein cycleHCR targeting the active enhancer H3K4me1, active chromatin H3K27ac, inactive chromatin H3K27me3, and nuclear pores Nup98 in the hippocampal slice.

Scale bars: 50 µm.

**
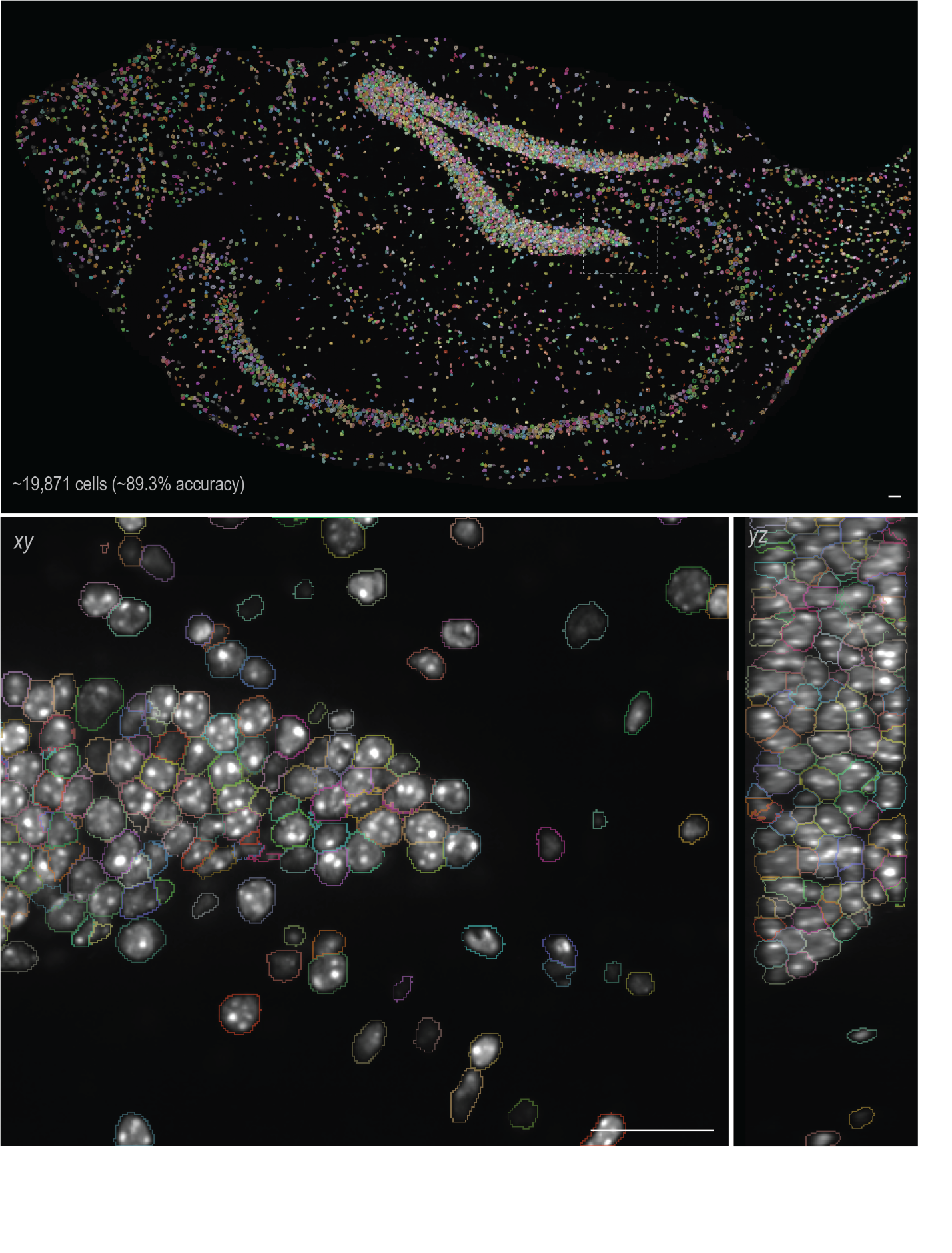
**

**fig. S35. 3D segmentation of hippocampal slice for joint protein and RNA cycleHCR imaging.**

The upper panel displays a slice view of segmented cells within the hippocampal slice by Cellpose, utilizing three-view segmentation of human-in-the-loop custom-trained models trained on orthogonal views (*xy* and *yz*). Contours delineate DAPI-stained nuclei. The lower two panels show the *xy* and *yz* views of the boxed region in the upper panel. Images were rendered using ORS Dragonfly.

Scale bars: 50 µm.

**
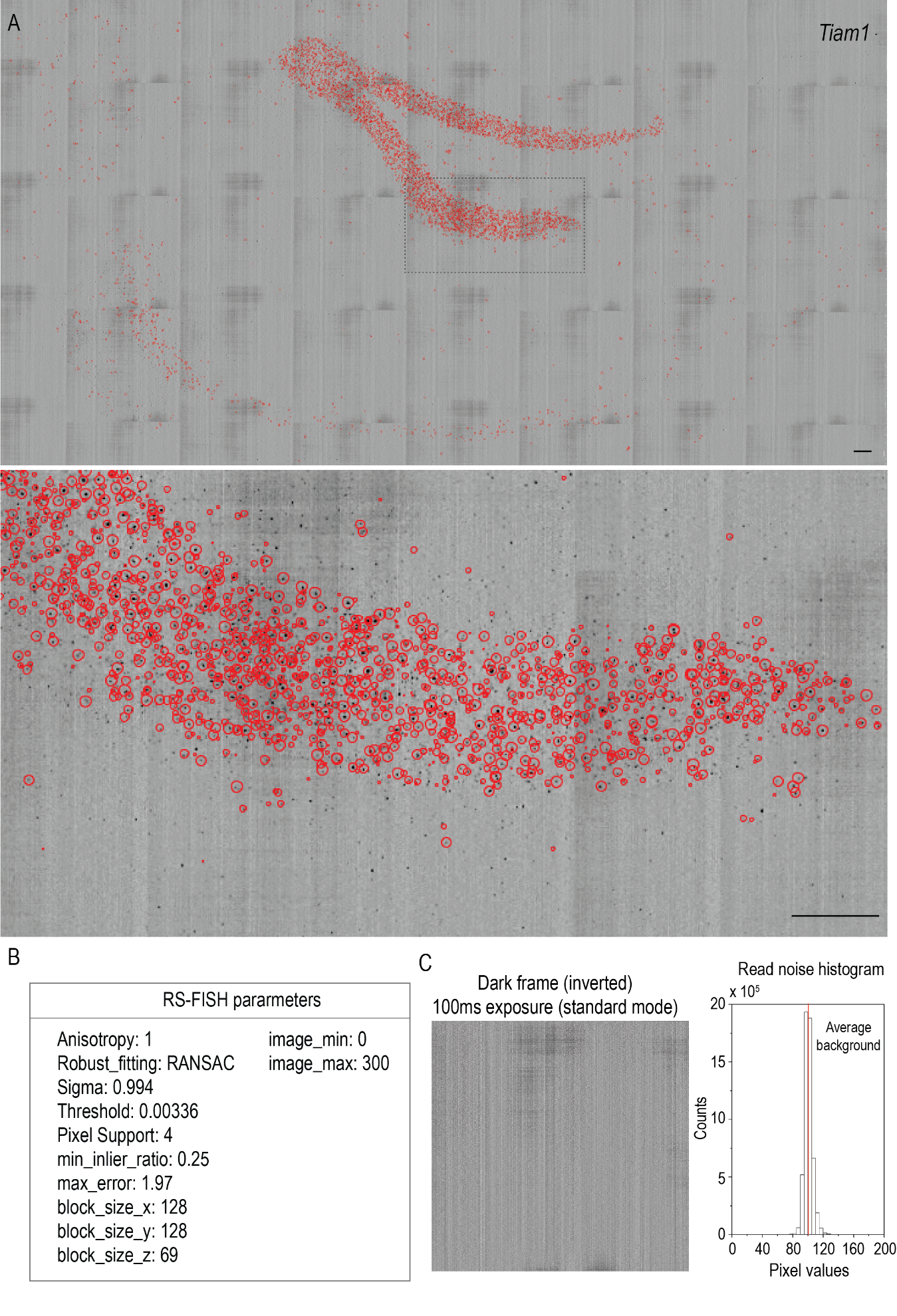
**

**fig. S36. Spot detection for joint protein and RNA cycleHCR imaging of hippocampal slice.**

(A) Spot detection in imaging mRNA transcripts of the *Tiam1* gene in the hippocampal slice. Red circles indicate the positions of localized single molecules detected by RS-FISH, overlaid on an inverted raw image. A zoomed-in view of the boxed region is shown below. Scale bar: 50 µm. Image stitching was performed using one-tile-wins strategy as detailed in the method part to avoid any localization artifact caused by image fusion.

(B) RS-FISH parameters for localizing spots. These parameters were carefully selected to minimize false positive detections and were maintained consistent for processing data from all cycles.

(C) Inverted dark frame image acquired with a 100 ms exposure time from the Hamamatsu BT fusion camera in standard mode, showing the broad read noise pattern on the camera chip. As expected, read noise is tightly centered around the average background value of approximately 100 units (red line). The read noise pattern does not noticeably influence spot detection as shown in (A).

Scale bars: 50 µm.


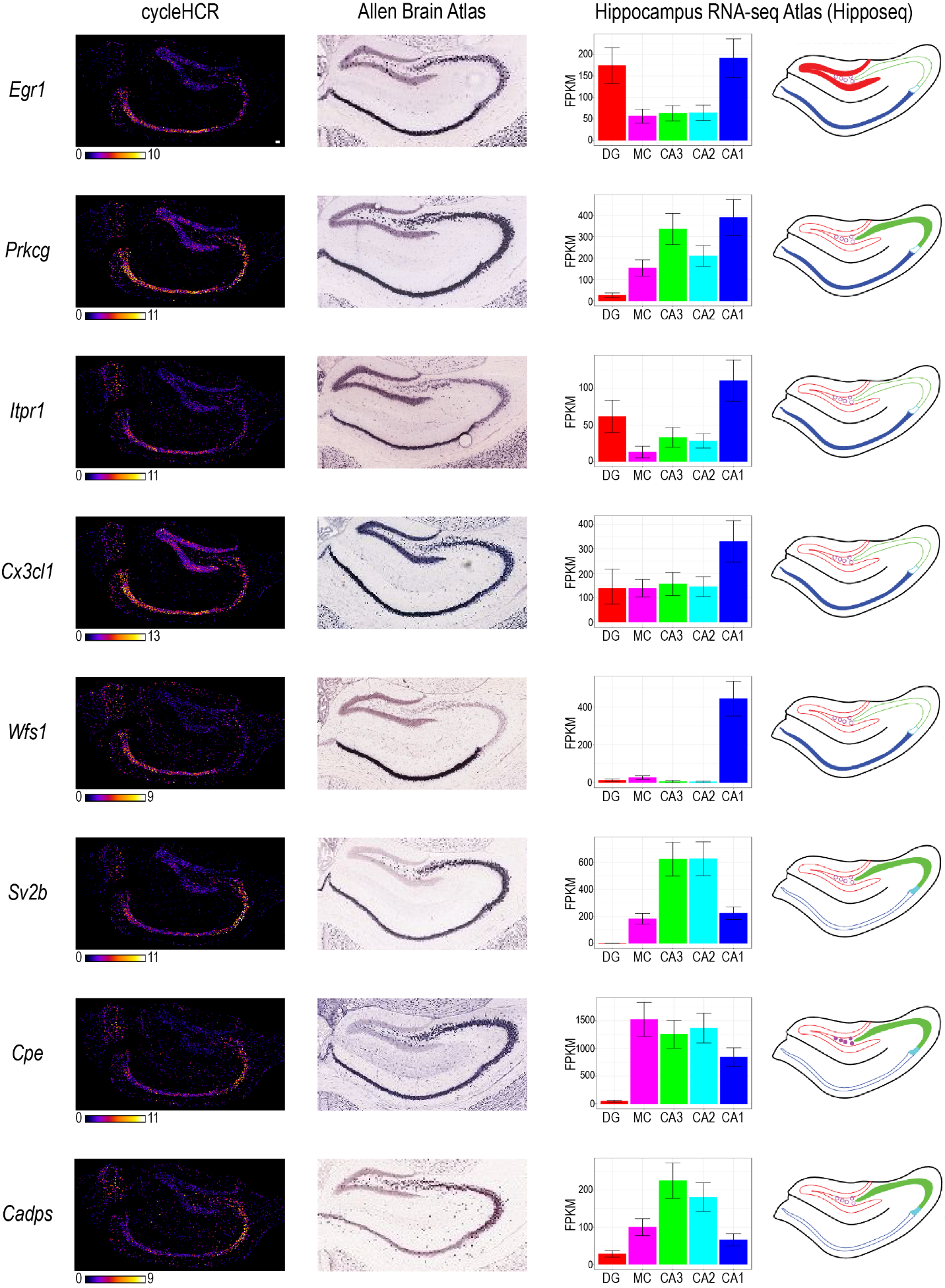


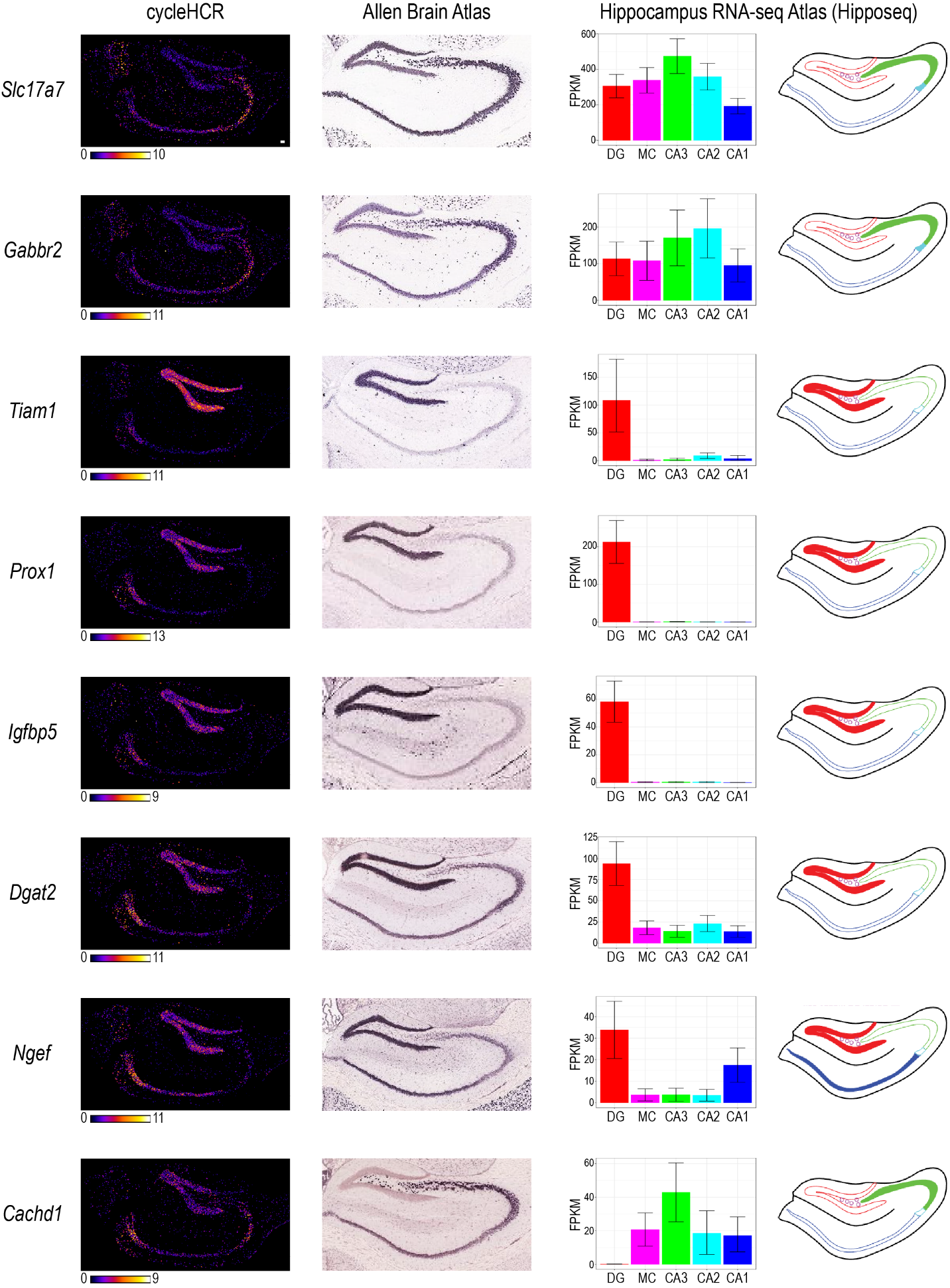

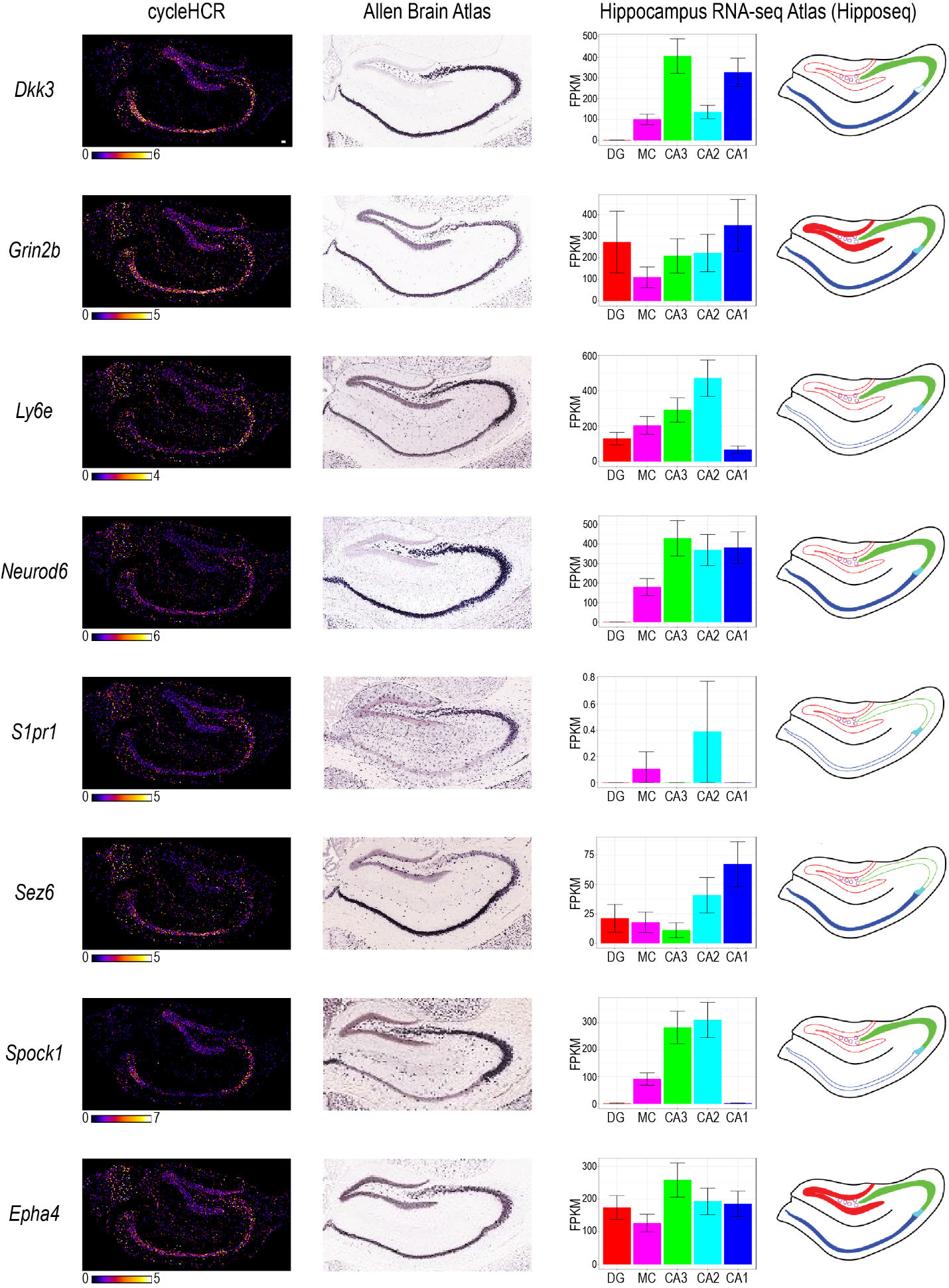


**fig. S37. Cross-method comparison with spatial RNA-seq and ISH data.**

A representative 2D slice of cycleHCR gene expression is shown on the left. The middle panel displays ISH data from the Allen Brain Atlas (*58*). The right-panel shows gene expression in major cell types from Hipposeq data (*57*). The structures expected to show high expression based on FPKM are highlighted in the cartoon on the far right. Scale bar: 50 µm.

_
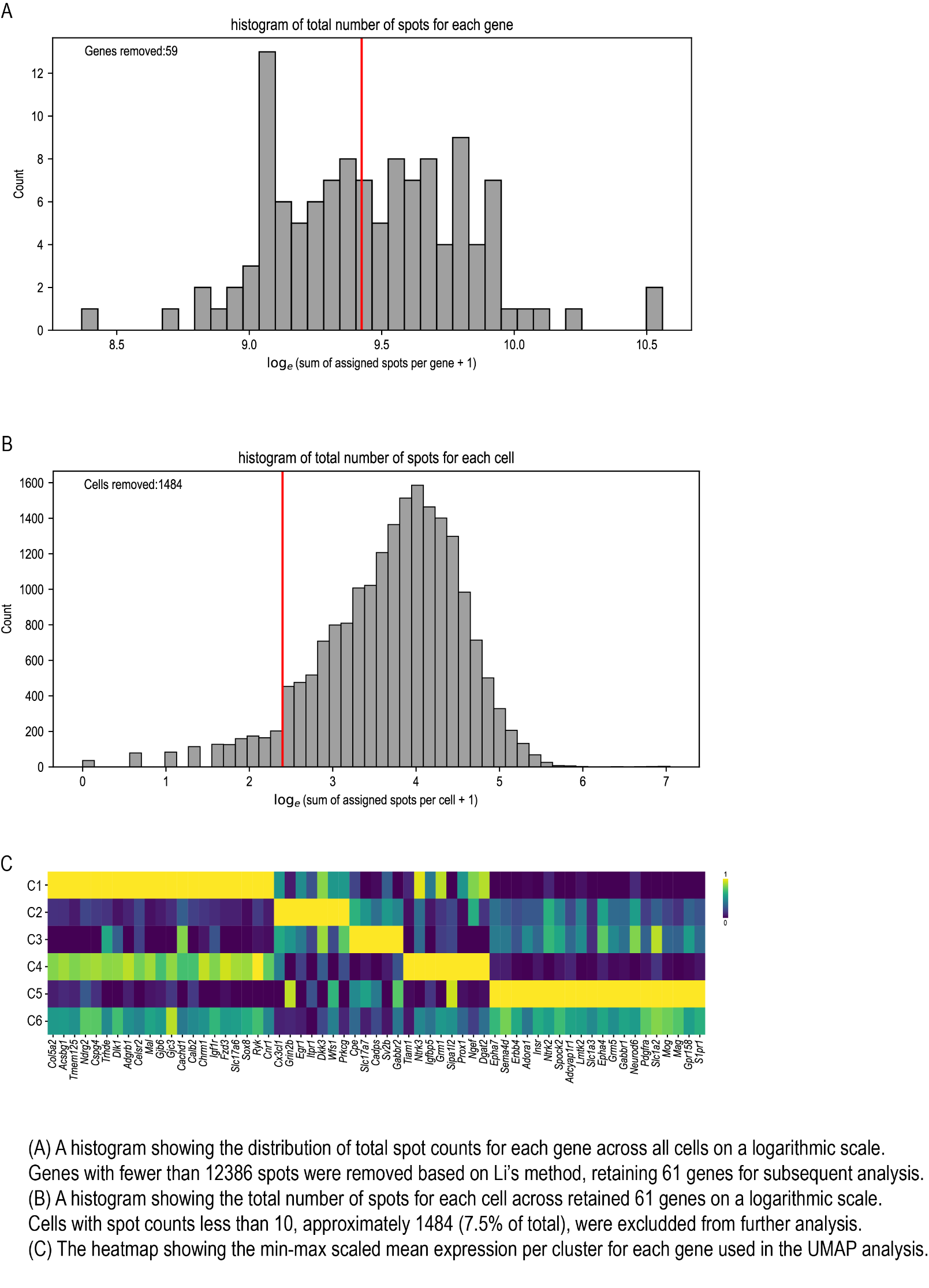
_

**fig. S38. Gene and cell filtering prior to UMAP analysis.**

(A) Histogram displaying the distribution of total spot counts for each gene across all cells on a logarithmic scale. Genes with fewer than 12,386 spots were removed based on Li’s method (*79*), retaining 61 genes for subsequent analysis.

(B) Histogram illustrating the total number of spots for each cell across the retained 61 genes on a logarithmic scale. Cells with spot counts less than 10, approximately 1,484 (7.5% of total), were excluded from further analysis.

(C) Heatmap presenting the min-max scaled mean expression per cluster for each gene used in the UMAP analysis.


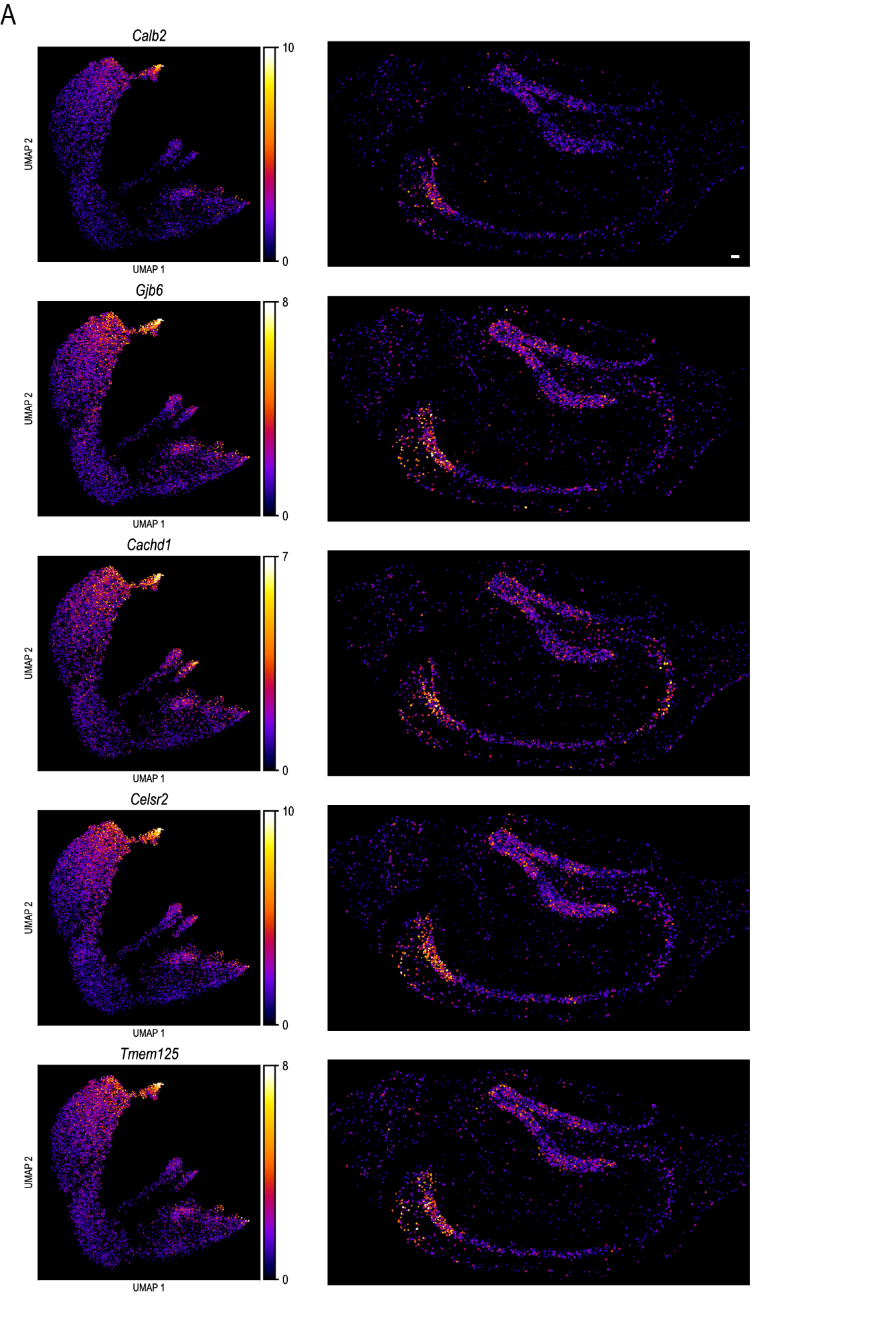


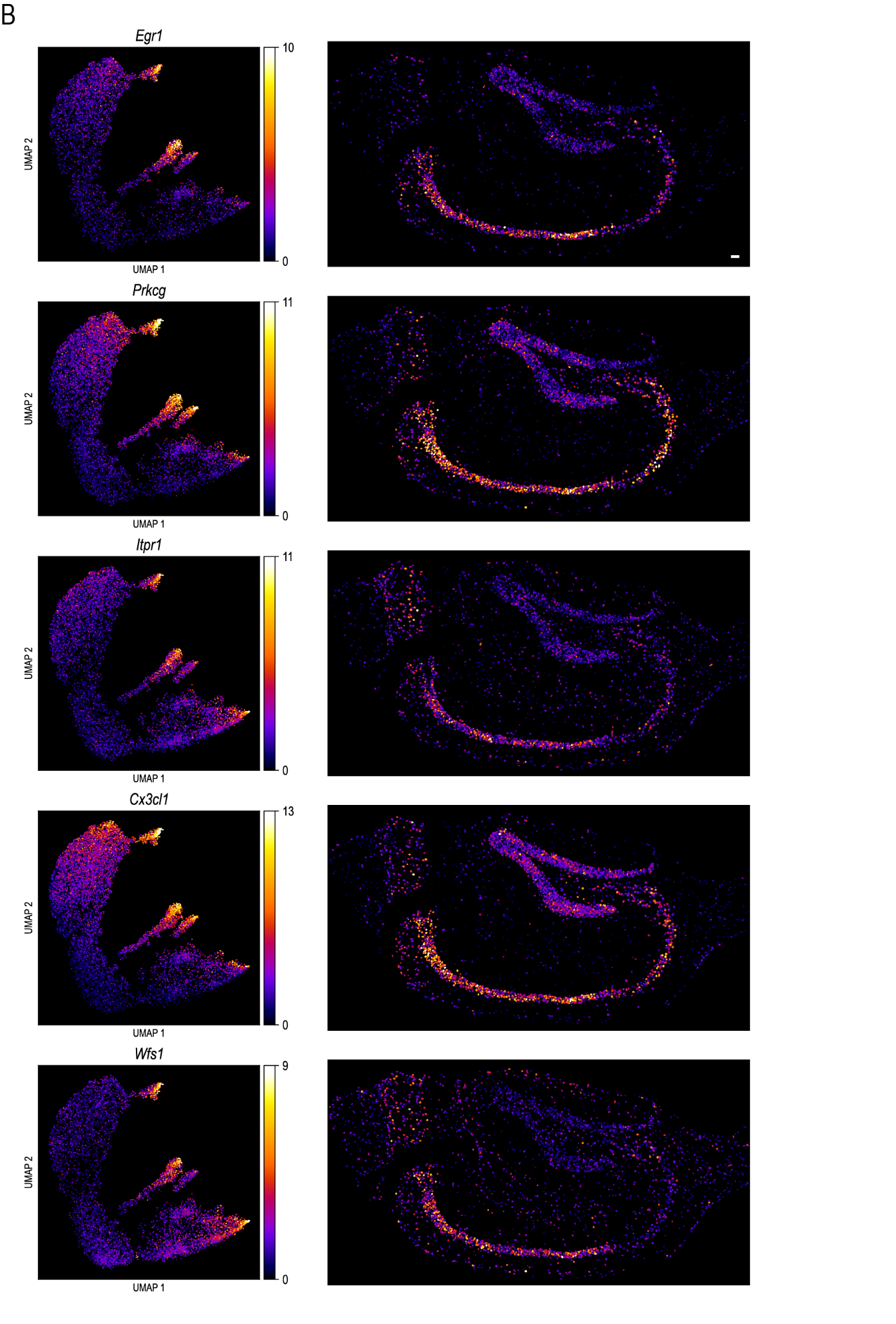

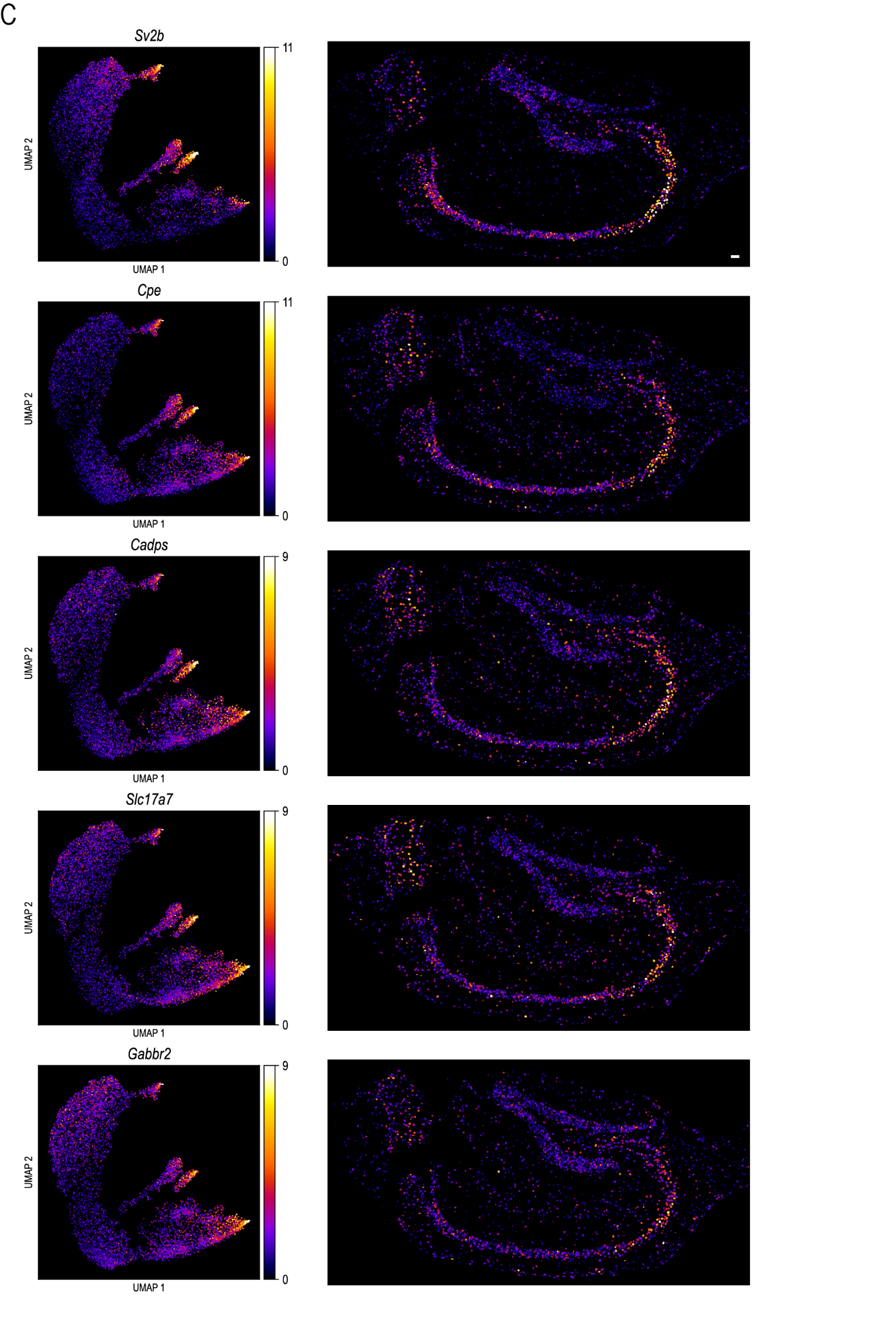


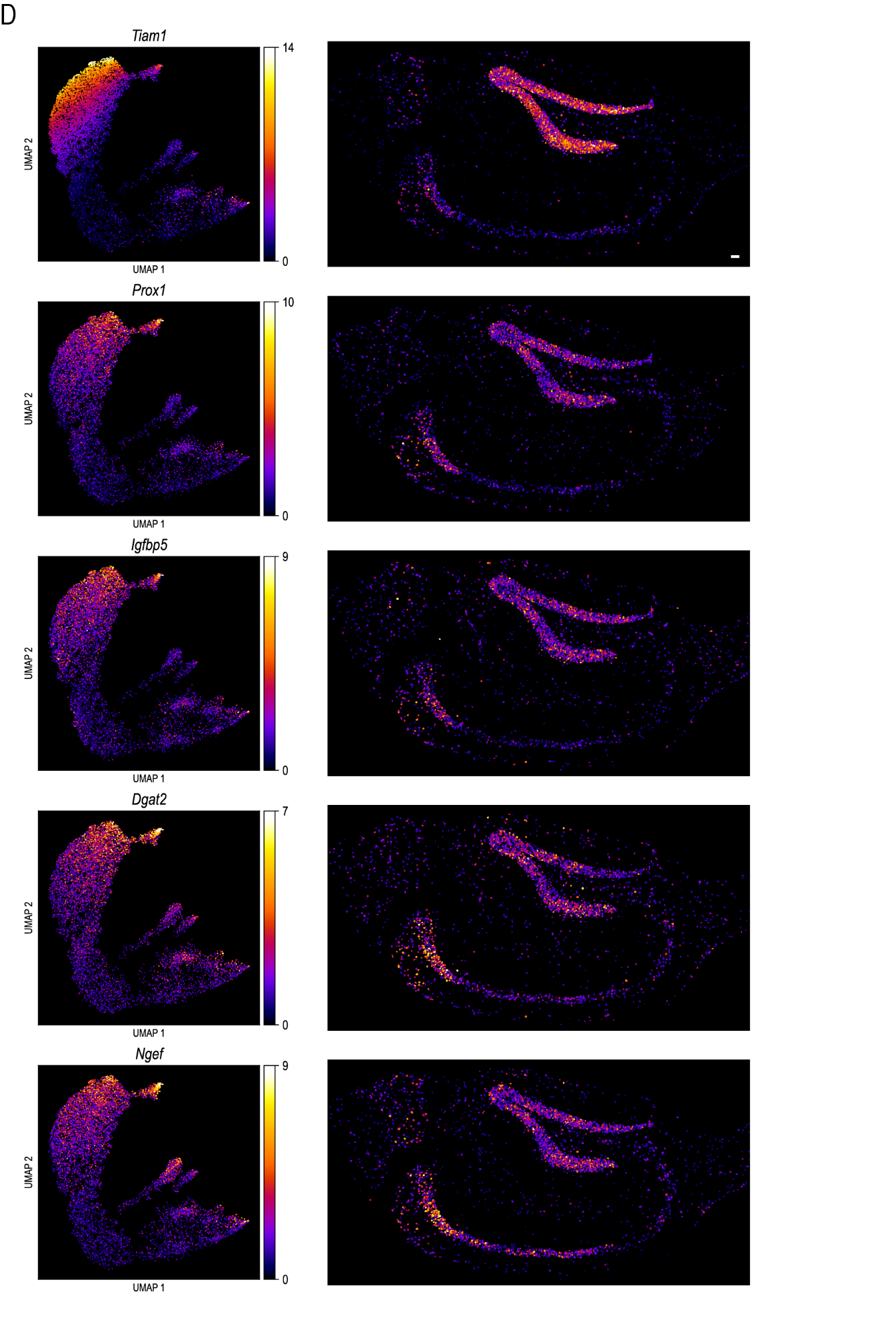

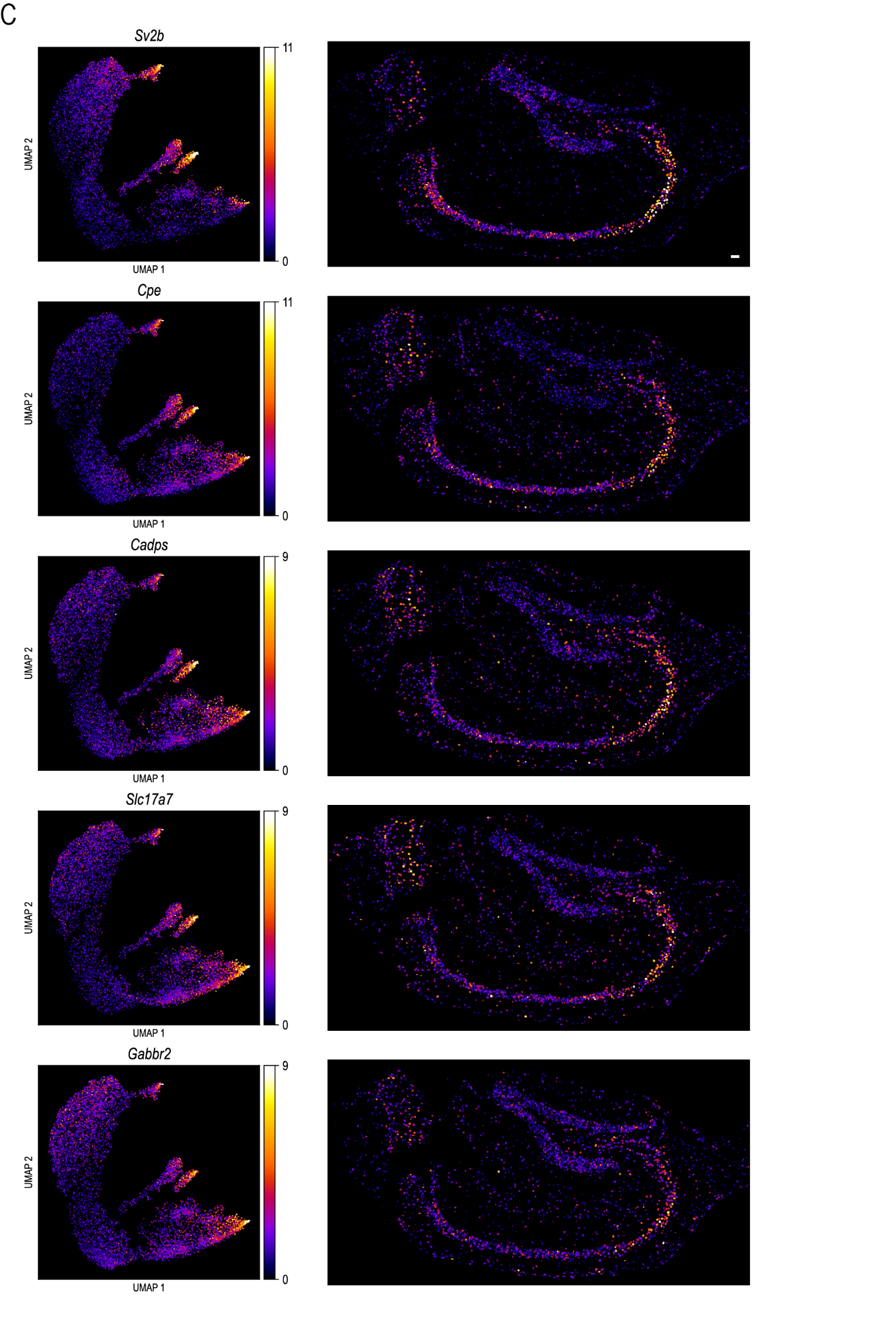


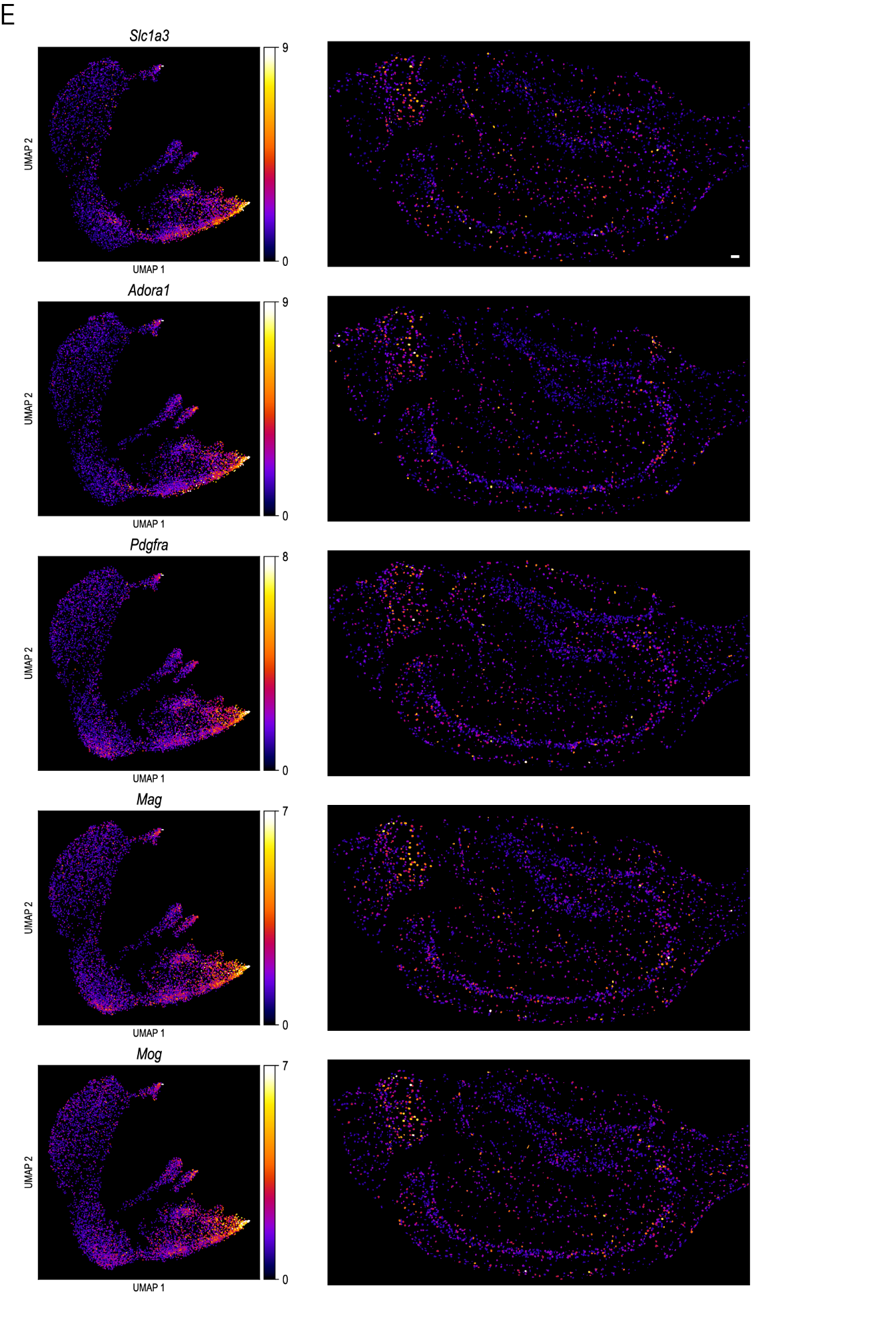


**fig. S39. UMAP imputation of genes for hippocampal clusters.**

**(A-E)** Gene expression levels for five selected genes for each of the five clusters are color-coded in the left panel, with panels A-E representing clusters 1-5, respectively, providing a visual representation of gene expression variations among clusters. The right panel offers selected *xy* views of the hippocampal slice, illustrating the spatial distribution of gene expression. Unified color maps adjacent to the UMAP panel aid in interpreting the gene expression levels. Scale bar: 50 µm.


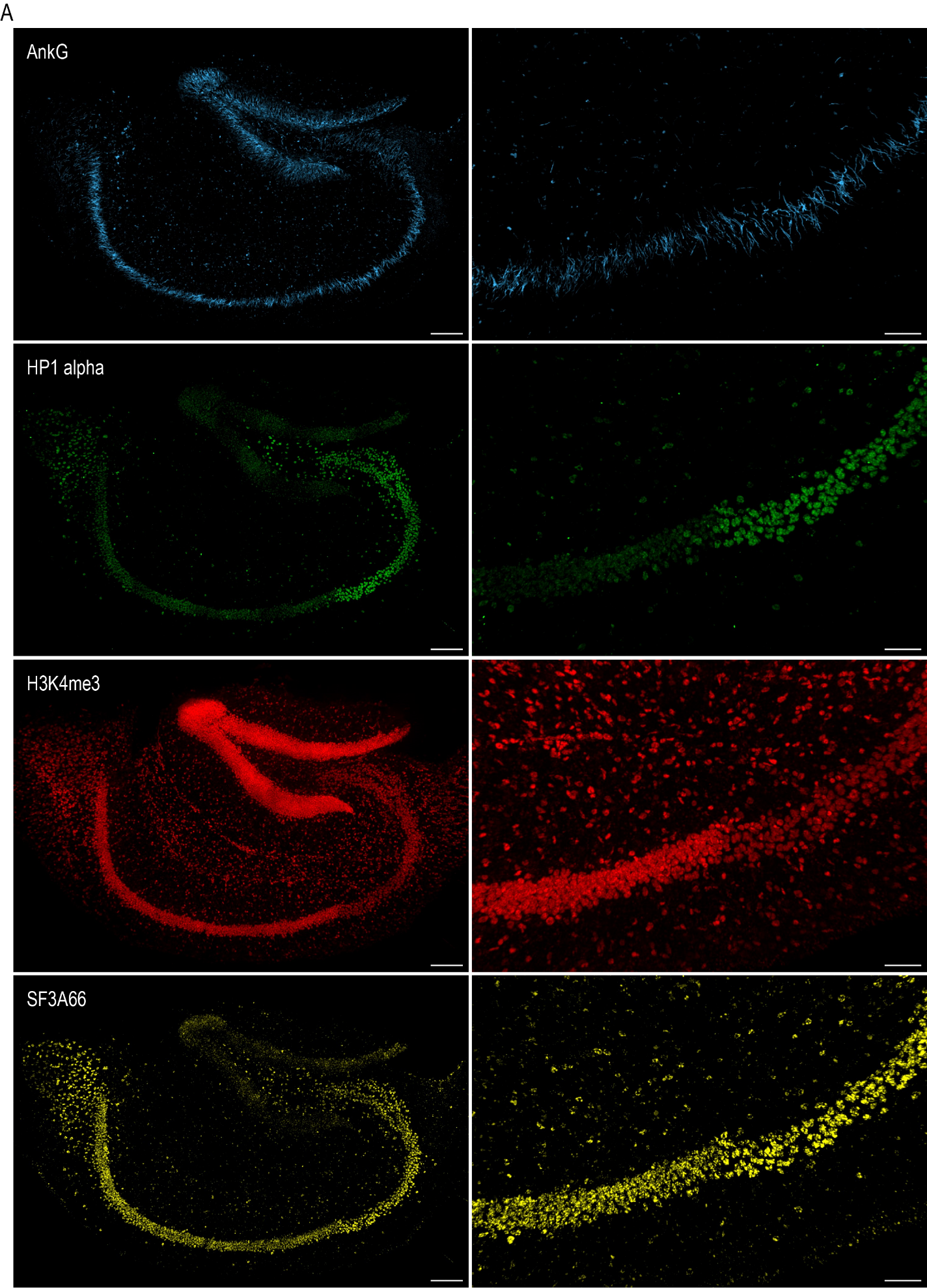


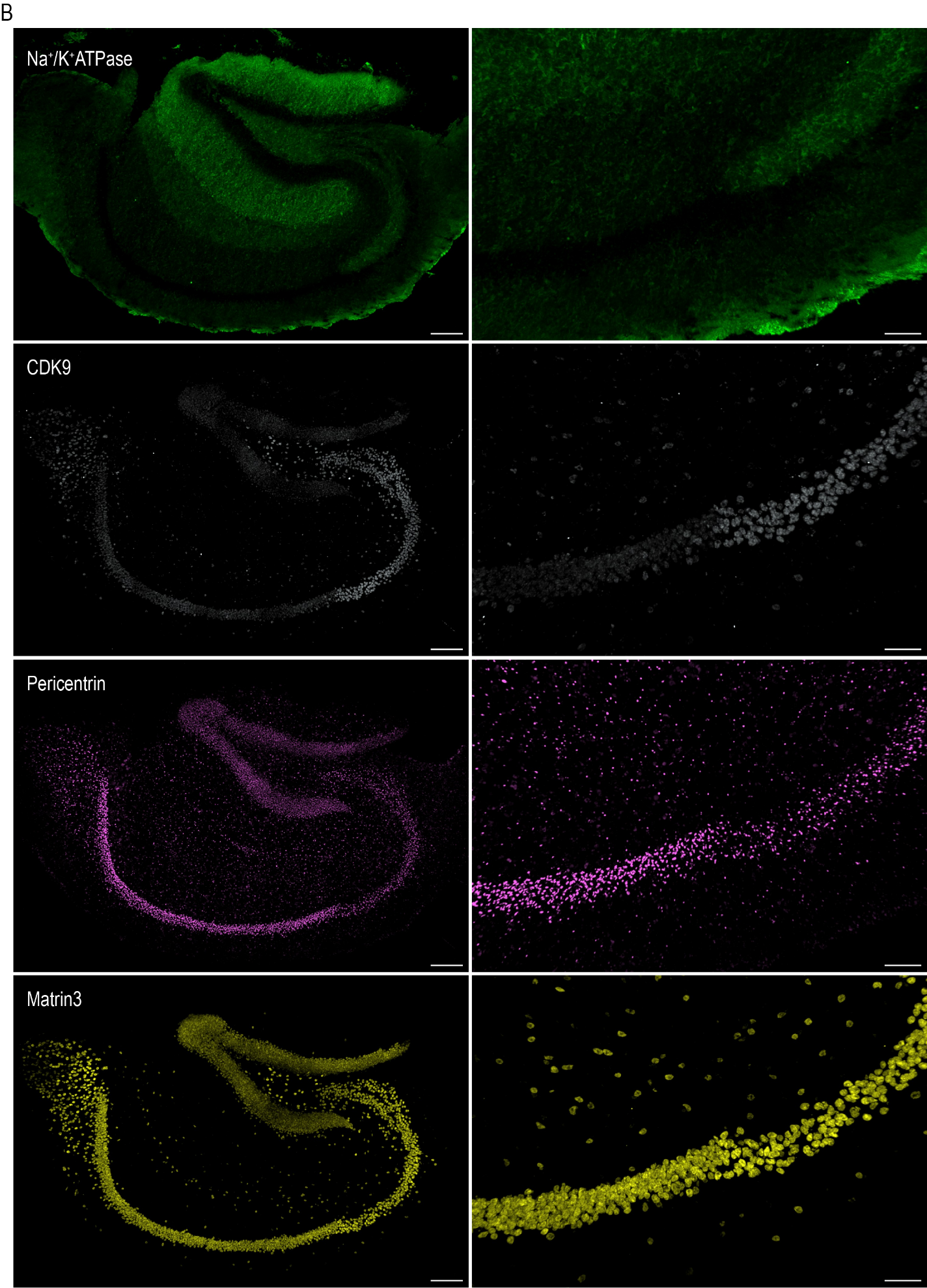


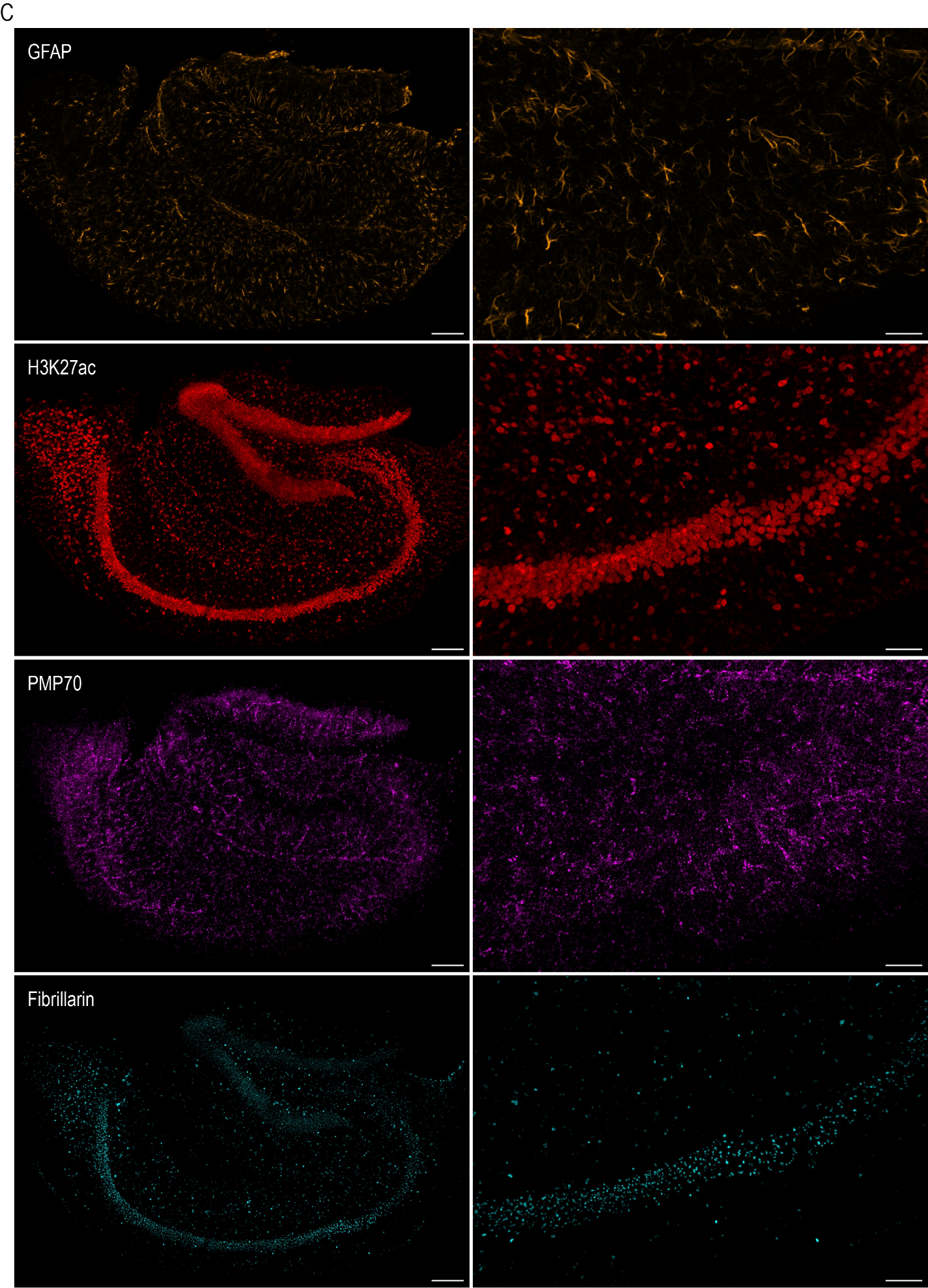


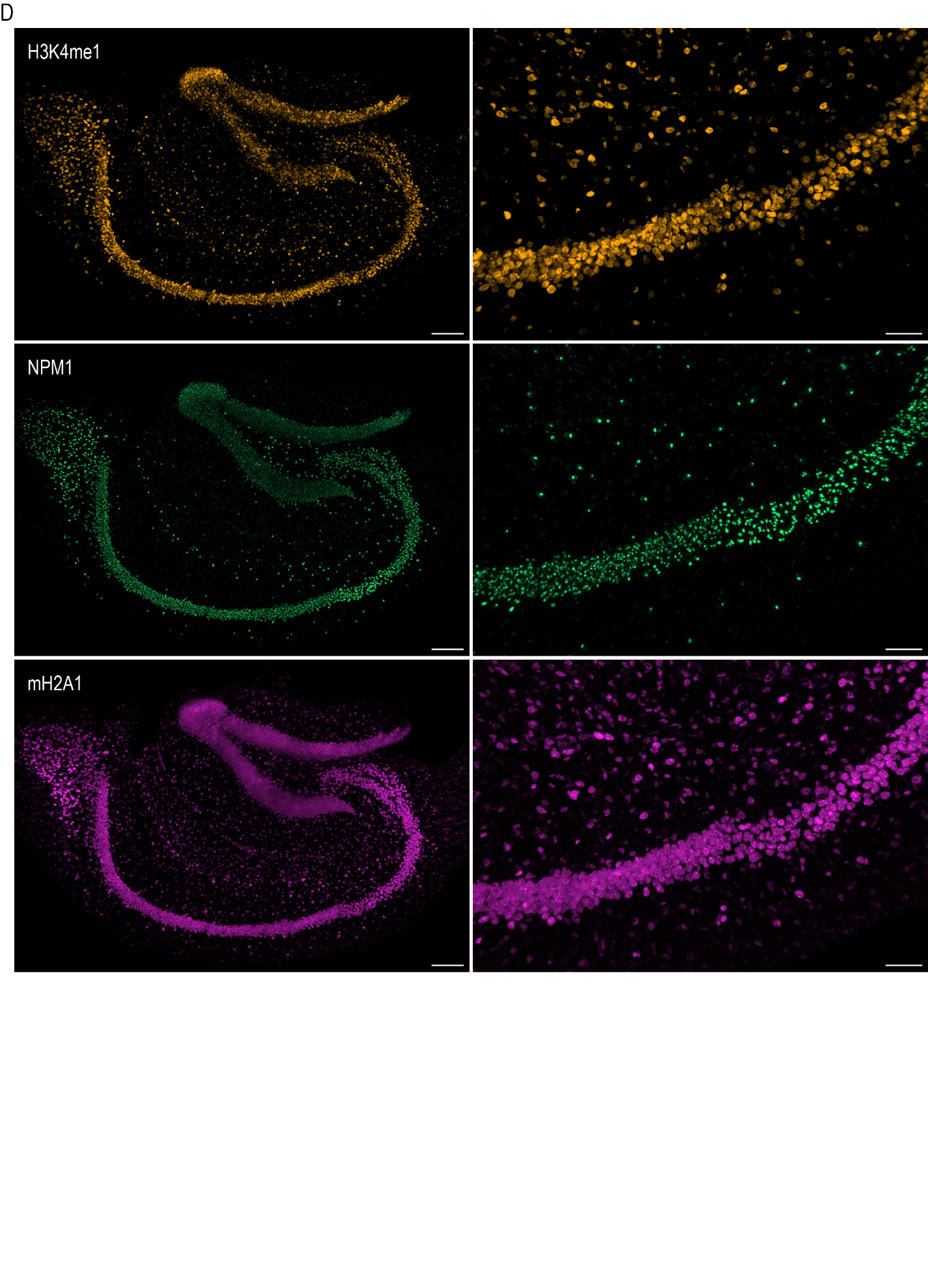


**Fig. S40. Protein cycleHCR in hippocampal slice repeat (15 antibodies).**

The panels A-D display stitched and registered images from the repeat of multiplex protein and RNA cycleHCR imaging in the hippocampal slice. The zoomed-in images are shown on the right panel.

(A) The axon initial segment marker AnkG, heterochromatin marker HP1 alpha, core promoter marker H3K4me, and nuclear speckle marker SF3A66.

(B) The membrane marker Na^+^/K^+^ ATPase, transcriptional elongation factor CDK9, the centrosome marker pericentrin and nuclear matrix marker Matrin3

(C) The glial marker GFAP, the euchromatin marker H3K27ac, the peroxisome marker PMP70 and the fibrillar center marker fibrillarin

(D) The euchromatin marker H3K4me1, the nucleolus marker nucleoplasmin (NPM1), and the histone variant mH2A1

Scale bars: 150 µm (left); 50 µm (right).


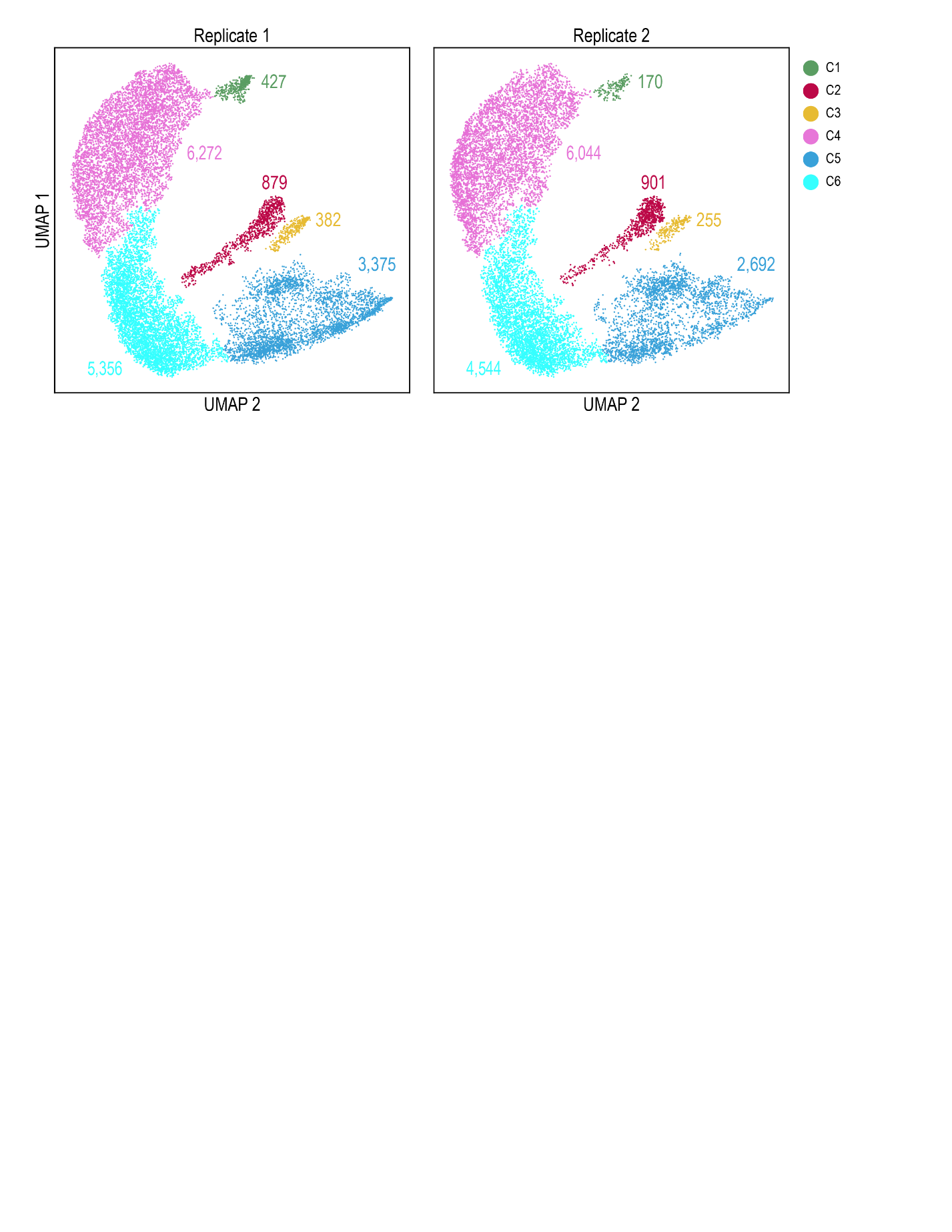


**fig. S41. UMAP analysis of cycleHCR in hippocampal slice replicates**

UMAP analysis of single-cell transcript counts in two replicates of hippocampal slices. The number of cells within each cluster is indicated in each panel.


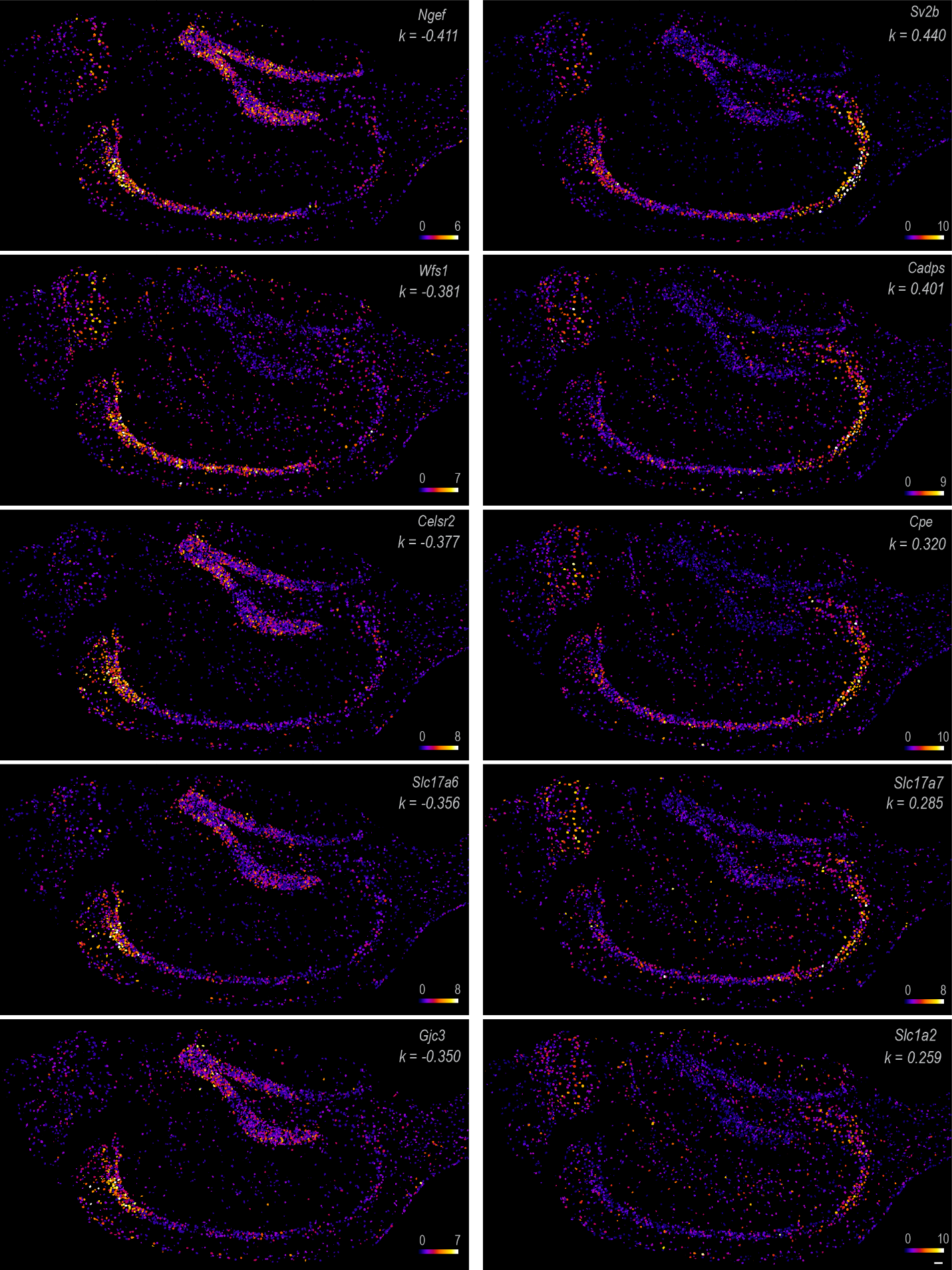


**fig. S42. Gene expression gradients along the C1-C2-C3 axis.**

On the left are gene expression maps for 5 genes with the steepest decreasing slopes along the C1-C2-C3 segments shown in Fig. 6F, while on the right are 5 genes with the steepest increasing slopes. Cells are color-coded according to spot count, as indicated by the color bar.

Scale bars: 50 µm.


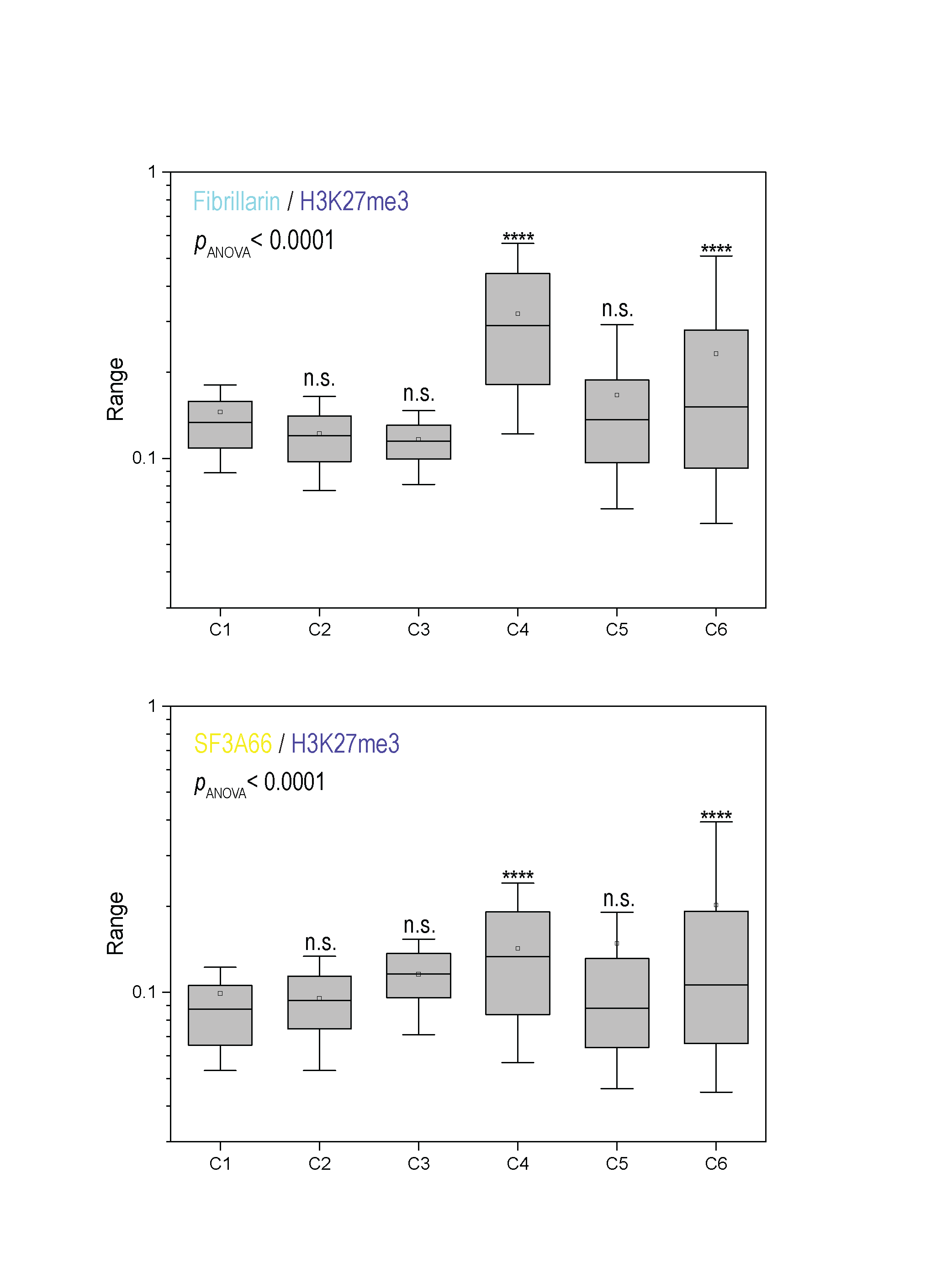


**fig. S43. Nuclear structural variations among UMAP clusters in hippocampus.**

Boxplots depicting nuclear antigen levels (fibrillarin – nucleolar fibrillar region; SF3A66 – nuclear speckles) normalized to inactive chromatin (H3K27me3) intensity for all clusters identified in the UMAP analysis of hippocampal cells. The lower and upper whiskers represent the 10th and 90th percentile values, respectively; the box represents the interquartile range (IQR) from the 25th to 75th percentile; the center line denotes the median; and the dotted line indicates the mean. The number of cells for each cluster is annotated in Fig. 6E. A one-way ANOVA test was first performed to assess whether significant differences were present among the 6 groups, followed by individual Bonferroni tests using C1 as the reference group. n.s., non-significant (*p* > 0.05); ****, *p* < 0.0001.

**List of Supplementary Tables:**

**Table S1**. L + R barcoding probe sequences for RNA mapping.

**Table S2**. Barcodes and probe sequences used in cycleHCR imaging of 254 genes in the E6.5-7.5 mouse embryo.

**Table S3**. Spot counts under stringent and relaxed localization conditions for whole-embryo transcriptomics imaging.

**Table S4**. Cell positions, transcript count per cell for 254 genes and UMAP assignment in the E6.5-7.5 mouse embryo.

**Table S5.** Gene expression slope for each cluster calculated for the proximal-distal, the anterior-posterior and the radial axes as defined by Fig. 4A.

**Table S6.** Gene co-expression correlation matrix for 9 clusters as shown in Fig. 4F.

**Table S7**. Protein cycleHCR antibodies and probe sequences.

**Table S8**. Barcodes and probe sequences used in cycleHCR imaging of 120 genes in hippocampal slices.

**Table S9**. Cell positions, transcript count per cell for 120 genes and UMAP assignment in mouse hippocampal slice.
